# Assessing cumulative impacts of human-induced pressures on reef and sandbank habitats and associated biotopes in the northeastern Baltic Sea

**DOI:** 10.1016/j.marpolbul.2022.114042

**Published:** 2022-10

**Authors:** Annaleena Vaher, Jonne Kotta, Robert Szava-Kovats, Ants Kaasik, Mihhail Fetissov, Robert Aps, Anneliis Kõivupuu

**Affiliations:** University of Tartu, Estonian Marine Institute, Tallinn, Harjumaa, Estonia

**Keywords:** Maritime spatial planning, Cumulative impact analysis, Sustainable development, Benthic habitats, Baltic Sea

## Abstract

Marine ecosystems are impacted by multiple individual and combined anthropogenic pressures. We used meta-analysis and data-driven PlanWise4Blue decision support tool to predict individual and combined impacts of wind park development, nutrient loading, and invasive species on vulnerable reef and sandbank habitats and associated species-specific biotopes in the northeastern Baltic Sea. Many impacts were not statistically significant due to large between-study variance in effect sizes. Wind park development is predicted to have less impact than nutrient loading and invasive species. Predicted impacts varied greatly among larger-scale habitats versus smaller-scale biotopes with impacts being generally stronger at small scale. Excessive nutrient loading damages algae-based biotopes, the presence of nonnative species has substantial negative impacts on larger-scale reef and sandbank habitats. The results showed that a 25 % reduction of nutrient loading improves all examined benthic habitats, whereas nonnative species, which cannot be removed from ecosystems, pose a significant threat to these habitats.

## Highlights

• The predicted human-induced impacts are weaker at large scales than small scales.
• Excessive eutrophication causes a permanent habitat loss on benthic environments more than any other studied human pressure.
• Substantial nutrient reduction will result in a significant increase in the areal coverage of valuable benthic habitats.
• Wind park development exhibited the lowest impacts on the studied benthic habitats.
• Cumulative impact assessments should be based on scientific information that is expanding and can be systematically searched.

## 1 Introduction

In a rapidly developing and changing world, it is crucial to find balance between the economic growth and the global environmental footprint (Robinson, 1993). Marine ecosystems are the largest of Earth's aquatic ecosystems and vital for human population - every second breath we take comes from the ocean. Natural values in marine ecosystems include various types of flora, fauna, and habitats – important resources for sustaining economic development that rely on minerals, fish, and pharmaceutical compounds, support services such as oxygen and clean water production, and temperature regulation (UNCTAD, 2016). Marine ecosystems are heavily impacted by human-induced pressures such as exploitation, eutrophication, pollution, and the introduction of nonnative species (Halpern et al., 2008; Burrows et al., 2011).

Maritime spatial planning (MSP) is a powerful instrument to put “ocean space” on sustainable development agendas. MSP can help nations achieve their goals of sustainable development, but only if the planning solutions are supported with a solid evidence-based understanding of how human-induced activities affect marine ecosystems. MSP makes this empirical knowledge available to diverse groups of marine stakeholders: scientists, politicians, fishermen, and entrepreneurs. As such, MSP is a process that enables stakeholders to achieve ecological, economic, and social objectives to ensure effective long-term use of marine resources and to mitigate multi-sectoral conflicts over the use of the sea space (Douvere and Ehler, 2010; Stelzenmüller et al., 2015.; EU, 2014; Aps et al., 2018).

Global demand for strong and clear communication between all marine stakeholders is rising. This demand leads to implement tools such as MSP to assemble and utilize evidence-based knowledge and to for long-term plans for sustainable management. However, assessment of pressure-specific human activities is challenging because human activities have simultaneously individual and combined impacts on the marine habitats. One way to synthesize complex scientific knowledge into comprehensible information, is to create Decision Support Tools (DSTs). These tools provide marine stakeholders with prediction models for examining individual and combined impacts of management proposals for sustainable development planning (Kotta et al., 2020).

The Baltic Sea represents an excellent testing ground for assessing DSTs as the sea is surrounded by nine industrialized countries and impacted by multiple anthropogenic pressures (HELCOM, 2016). The largest threat to the ecosystems in the Baltic Sea is eutrophication (HELCOM, 2018). Eutrophication triggers harmful algae blooms, which leads to oxygen deficiency in benthic communities, ultimately causing biodiversity loss and a decline in valuable habitats (HELCOM, 2017). Another major threat to the integrity of the Baltic Sea is caused by the presence of nonnative species (Ojaveer and Kotta, 2015). Introduced species can substantially change local biodiversity, modify the structure and functions of aquatic ecosystems, and alter ecosystem services (Bax et al., 2003; Katsanevakis et al., 2014; Ojaveer et al., 2015).

Wind energy represents one method through which to achieve climate neutrality. Developing renewable energy will minimize the environmental impact of the energy sector, strengthen energy security, and increase the economy's competitiveness (Hendrikson & Ko, 2021). The recently adopted Estonian MSP has proposed three offshore wind farm areas with a total surface area of 1783 km^2^. These farms will be developed in the near future to shift from fossil fuels to renewable energy production, thereby reducing greenhouse gas emissions (Hendrikson & Ko, 2021). At the same time, offshore wind farm foundations have the potential to serve as artificial habitats and havens for a variety of organisms (Degraer et al., 2020).

This study uses the PlanWise4Blue (PW4B) tool (Kotta et al., 2020) to predict the environmental consequences of feasible management scenarios on benthic habitats in Estonian waters. PW4B tool is the first data-driven DST on cumulative impact analysis that takes advantage of a plethora of scientific evidence to define the individual and/or combined impacts of human-induced pressures on ecosystems (Depellegrin et al., 2021). Most of the other developed DSTs rely on expert judgement to define responses of ecosystems to pressures caused by different human activity (Jones et al., 2018).

The scenarios presented in this study focus on different types of human pressures: nutrient loading (managed on land), wind park development (managed at sea), and nonnative species (represents largely unmanageable pressure when already present in a marine ecosystem). The nutrient loading scenarios included a business-as-usual projection (the current amount of nutrient input) and the HELCOM MAI target (nutrient input reduced by 25 %). Wind park scenarios included the areas of offshore wind farms as projected by the Estonian maritime spatial plan (Hendrikson & Ko, 2021). The nonnative species scenarios included the two most influential invasive species in the region: Ponto-Caspian round goby, *Neogobius melanostomus*, and North American mud crab, *Rhithropanopeus harrisii*. Both species arrived in the northeastern Baltic Sea in the early 2000s (Ojaveer, 2006; Kotta and Ojaveer, 2012) and have since significantly modified local coastal environments, the latter being associated with intensifying symptoms of eutrophication (Ojaveer et al., 2015; Kotta et al., 2016; Kotta et al., 2018).

When running these scenario analyses, we tested the following hypotheses: (1) due to different directions of impact across biotope forming species, the predicted human-induced impacts are weaker at large scales (i.e. reef and sandbank habitats) than small scales (biotopes associated to habitats), (2) as excessive eutrophication causes a permanent habitat loss, substantial nutrient reduction will result in a significant increase in the areal coverage of valuable benthic habitats, (3) the strong predation of nonnative species on biotope forming species will modulate the response of habitats to other pressures, (4) wind parks are expected to increase the areal coverage of reef habitats as the foundation of wind turbines acts as a stable artificial substrate for many reef-building organisms such as mussels (*Mytilus trossulus*) and macro-algae.

## 2 Methods

### 2.1 Description of study area

The Baltic Sea is a shallow brackish water body characterized by strong seasonality and highly variable environmental gradients including salinity, temperature, wave exposure, and bathymetry (Carstensen et al., 2014; Zettler et al., 2014). The benthic ecosystems in the Baltic Sea are extremely vulnerable because only a few species are endemic to brackish water conditions; most marine and freshwater species in the Baltic Sea inhabit the edge of their physiological limits (Sjöqvist et al., 2015; Lauringson and Kotta, 2016; Johannesson et al., 2011). Therefore, species redundancy is low, yet large parts of coastal areas are covered by lush benthic ecosystems (Schubert and Telesh, 2017).

**Fig. 1:**
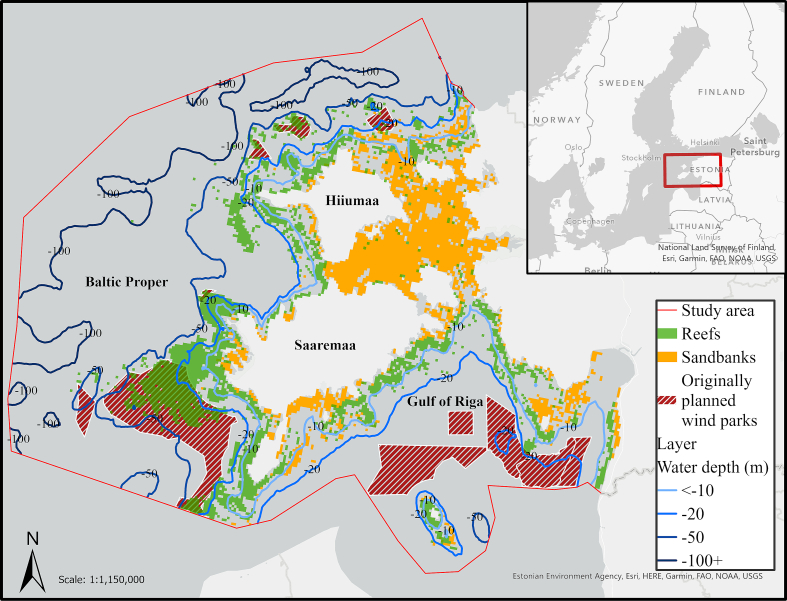
Study area. The red outline marks the Western Estonian waters where this study takes place. Green areas mark the location of reef habitat type and orange the sandbanks. Red dashed areas mark the planned wind park locations. Depth isobaths are shown with blue lines. (For interpretation of the references to color in this figure legend, the reader is referred to the web version of this article.)

The case study area is situated in the western Estonian waters of the northeastern Baltic Sea (Fig. 1). Salinity is constantly low, between 7 ppt in the offshore waters and nearly 0 ppt adjacent to river estuaries and within embayment areas. The average summer water temperatures are between 15 and 17 °C; however, in recent years, the average monthly sea surface temperatures have reached up to 25 °C (Siegel et al., 2006; HELCOM, 2021). However, near inner bay area, zero temperatures are typical in winter and ice cover may last for several months (Sooäär and Jaagus, 2007). The seafloor in the study area is primarily hard bottom near the coast and soft bottom offshore. In near-coastal areas the substrate is limestone or dolomite banks; the offshore substrate includes mixed sand, gravel, and boulders. The maximum depth in study area is 200 m (southwest corner of study area in the Baltic Proper, Fig. 1).

We differentiated the studied benthic ecosystems into reef and sandbank habitats, distinguished by bottom substrate and species composition. Multiple species-abundant benthic ecosystems, such as sandbanks and reef habitats, make the study area important for marine conservation. These habitats were further distinguished into habitat-associated biotopes by the presence of key species. The most common biotopes in the reef habitat are distinguished by rockweed (*Fucus vesiculosus*), red seaweed (*Furcellaria lumbricalis*), and the suspension-feeding bay mussel (*Mytilus trossulus*). The biotope-distinguishing species in the sandbank habitats are Charophytes, common eelgrass (*Zostera marina*), and other higher order plants (Martin et al., 2013, Fig. 2).

**Fig. 2:**
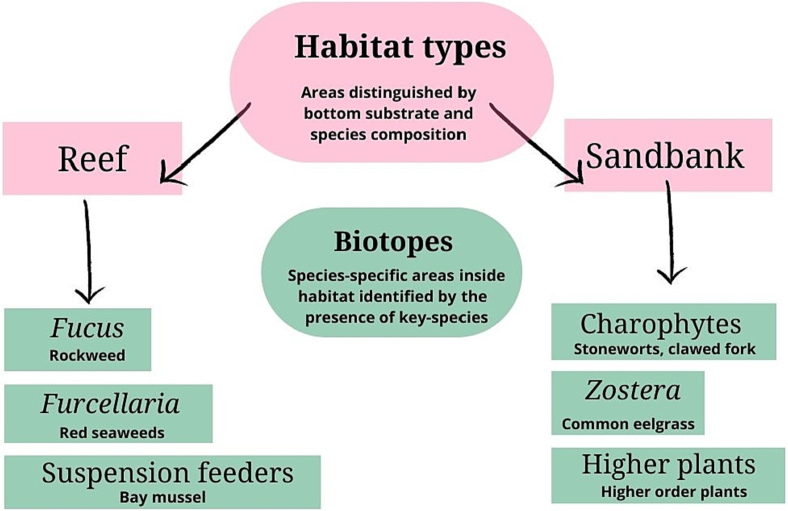
We divided benthic ecosystems into reef and sandbank habitat types according to bottom substrate and species composition. Furthermore, the habitat types were divided into species-specific biotopes: *Furcellaria*, *Fucus*, and suspension feeders are associated with reef habitats and *Zostera*, Charophytes, and higher plants are associated with sandbank habitats.

### 2.2 PlanWise4Blue

The PW4B is a user-friendly geoportal tool that combines novel spatial modelling products of environmental background (e.g. maps of benthic habitats) with spatial data related to the use of marine resources (Kotta et al., 2020). The PW4B tool is based on ecosystem indicators that can quantify the intensity of ecosystem services (in contrast to many earlier assessments based solely on the presence/absence of ecosystem services). PW4B allows examination of individual and cumulative impacts of human-induced pressures on benthic habitats. In this study, for example, we could predict both the individual impacts of non-native species and nutrient loading on selected habitats as well as the combined impact of these two pressures. Because habitats are impacted by multiple human-induced pressures simultaneously, it is important to study the impacts of combined human pressures. PW4B aims to identify various human-induced pressures and account for their cumulative impacts on the natural environment, while also considering regional differences of nature. The spatial resolution of the model is 1 km^2^, and the temporal timescale is 1 year (Kotta et al., 2020).

The PW4B tool includes the most updated maps of benthic habitats and biotopes (native spatial resolution of these map layers is 100 m (for modelling approaches see Aps et al., 2018 and Torn et al., 2020). In this study we used maps of coverage of habitats and associated biotopes that provided a seamless prediction in the entire study area. Importantly, an areal reduction of habitat indicates not only change in habitat extent but also suggests other types of habitat degradation as species coverage and biomass are highly correlated in benthic ecosystems (Vahtmäe et al., 2021). A decline in habitat quality (e.g. decline in biomass of habitat-forming species) is expected to be followed by losses in the areal extent of habitats. As an output, the PW4B tool predicts shifts (both increases and decreases) in the areal coverage of all benthic habitats and associated biotopes caused by any combination of pressures defined under scenario.

### 2.3 Study scenarios

We decided to focus on three human activities: nutrient input, nonnative species, and wind parks. These human activities represent examples of pressures that can be managed on land (nutrient loading) or at sea (wind park development) or that are largely unmanageable (nonnative species already present in a marine ecosystem cannot be effectively removed).

The studied scenarios involved single-pressure impacts (scenarios 1–4) and multi-pressure impacts (scenarios 5–7) to compare the individual and combined impacts of different pressures on benthic ecosystems:

Scenario 1: Current nutrient load
Scenario 2: Future nutrient reduction (HELCOM MAI target of 25 % nutrient reduction)
Scenario 3: The presence of nonnative species (round goby and mud crab)
Scenario 4: Projected wind parks (according to the Estonian maritime spatial plan)
Scenario 5: Current nutrient load + nonnative species
Scenario 6: Current nutrient load + nonnative species + wind parks
Scenario 7: Future nutrient reduction + nonnative species + wind parks

For each scenario, environmental impacts of the respective pressure(s) were assessed separately for all studied benthic habitats and associated biotopes (Fig. 2). The spatial impacts of these human pressures (current and projected) on benthic ecosystems were generated by the PW4B portal (Kotta et al., 2020). Details on the spatial modelling of benthic habitats are available in Aps et al. (2018).

To achieve a healthy environmental status for water quality, the Helsinki Commission (HELCOM) has proposed reducing nitrogen and phosphorus concentrations in the sea. This will be achieved by setting the Maximum Allowable Inputs (MAI) and Country Allocated Reduction Targets (CART) for each HELCOM country. In the Baltic Sea, the MAI targets require a reduction of nitrogen and phosphorus of 11.2 % and 27.5 %, respectively (HELCOM, 2017). In our study scenario pertaining to future nutrient reduction, we simplified the HELCOM nutrient reduction targets by using an average reduction of both nitrogen and phosphorus loading of 25 %. Importantly, as the open Baltic Sea area is well interconnected, the nutrient reduction scenario involved the entire Baltic Sea basin, not only Estonian waters.

The establishment of round goby and mud crab significantly intensifies a function of benthic predation in the shallow-water environments that previously lacked benthic predators, or their densities were low (Nurkse et al., 2016; Nõomaa et al., 2022). We included these two invasive species to our study as impacts of both species on benthic ecosystems are strong and generally irreversible (Kotta et al., 2018; Nõomaa et al., 2022).

The environmental impact analyses of wind energy developments were based given technological specifications of the Estonian maritime spatial planning, i.e. wind turbines are built on a concrete foundation with a texture suitable for the attachment of seaweeds and large invertebrates and filled with stones. The wind turbine foundation is expected to be <100 m in diameter. The height of the concrete stem cone is 10 m. The maximum height of the wind turbine tip is 300 m, and the maximum diameter of the rotor is 250 m. The spacing between the wind turbines was estimated to be between 4 and 7 turbine diameters, i.e. a minimum of 800 m. The cumulative impacts model does not consider environmental impacts during construction, but the environmental impact of gravity foundations is less than other existing techniques. All planned wind park sites are located in 20–30 m overlapping with the studied benthic habitats. In these areas the seafloor is well oxygenated due to intense wind-driven waves and currents.

When assessing the environmental impacts of the studied pressures, the current algorithm of PW4B assumes that the impact of wind parks and non-indigenous species does not propagate spatially >500 m, which is delineated by our analysis grain of 1 km^2^. Therefore, the algorithm calculates the impacts of any set of pressures on the studied habitats/biotopes within a particular grid cell, but not in the neighboring cells. This specification is largely supported by the published evidence (see the lists of research paper used to build the impact matrix of the PlanWise4Blue in Supplementary material 1. However, this does not preclude the algorithm to calculate regional impacts when pressures occur in multiple grid cells; the cumulative impacts are a sum of impacts in all impacted grid cells.

The pressure map of nutrient loading used in the PW4B was based on simulations of the existing hydrodynamic models (the COPERNICUS product open access data BALTICSEA_ANALYSIS_FORECAST_PHY_003_006 and BALTICSEA_ANALYSIS_FORECAST_BIO_003_007 available at http://marine.copernicus.eu/services-portfolio/access-to-products/). This approach assured that the plausible consequences of different nutrient loading scenarios in terms of the spatial patterns of nutrient concentrations in seawater are well reproduced.

### 2.4 Meta-analysis of current knowledge and predicting impact of human pressures on benthic environments

Predicting the impact of human pressures on benthic environments entailed 1) a meta-analysis of published or raw data that indicated individual and/or combined impacts of the studied human-induced pressures – either from experimental manipulations or ecosystem changes observed before and after an impact, followed by 2) incorporation of these impacts into spatial predictions within different benthic environments as a cumulative impact assessment (e.g., Kotta et al., 2020). A list of references and further details to raw data from published literature and databases are included in the Supplementary material 1.

In order to predict the plausible impacts of different human pressures on benthic environments, we complied the current knowledge from published literature and available datasets. To prepare for the meta-analysis, we selected scientific articles that fulfilled the following criteria:

1. The study was conducted in the Baltic Sea. In a few cases when data from the Baltic Sea was limited (e.g., wind park development), we gathered data from an area that has similar benthic environments to the Baltic Sea, such as the North Sea region.
2. The study had comparable quantitative data from impact sites (with human pressure present) vs reference sites (without human pressure present). Subsequently, impact vs reference comparison could be made both spatially (between the impact site and the control site) or temporally (at different times within a single site).

Once a study fulfilled the criteria, we extracted the quantitative data concerning the impacts of human-induced pressures on the studied benthic habitats. For this study specifically, we extracted data that included research on wind park development, nutrient loading, or round goby/mud crab as human-induced pressures. We also filtered out habitat types and biotopes that we selected to examine in this paper (described above in Methods).

When possible, we extracted mean values with measurement units, standard errors, standard deviations, and sample sizes from impact and reference sites directly from tables and from the article text. When the data were presented in graphs, we used ImageJ software to extract relevant comparisons (Schneider et al., 2012). The extracted quantitative data were then used to calculate respective effect sizes. Mathematical formulae to calculate effect sizes and their corresponding uncertainty follow Kotta et al. (2020). The compiled scientific evidence (experimental and survey data) was uploaded to the PW4B portal to predict the environmental impact of the studied pressures on benthic habitats. The tool uses the habitat and pressure-specific coefficient of cumulative impacts in each region of interest, which were then multiplied by the corresponding result of the studied habitat to ascertain the expected changes (Kotta et al., 2020).

The analyses of this study are based on the following numbers of extracted data seen in Table 1. In addition to the PW4B analyses, the one-way analysis of variance (ANOVA) was used to assess the statistical significance of the impacts of studied human pressures on benthic habitats and associated biotopes and to compare their mean impact values.

**Table 1:** The number of observations of the compiled scientific evidence (experimental and survey data) to predict the environmental impact of the studied pressures on benthic habitats and associated biotopes. Note that sandbank habitat had no available data at the biotope scale.

Habitat/biotope	Human-induced pressure	Number of observations (N)
Reef	Round goby	13
	Round goby and mud crab	3
	Mud crab	13
	Wind park	57
	Nutrient input	86
*Fucus*	Mud crab	1
	Wind park	1
	Nutrient input	10
*Furcellaria*	Wind park	1
	Nutrient input	6
*Mytilus*	Round goby	7
	Mud crab	3
	Round goby and mud crab	1
	Wind park	1
Sandbank	Round goby	58
	Round goby and mud crab	1
	Nutrient input	78
	Mud crab	14
	Wind park	96

## 3 Results

### 3.1 Current impact evidence from scientific literature and databases

#### 3.1.1 Reefs

**Fig. 3:**
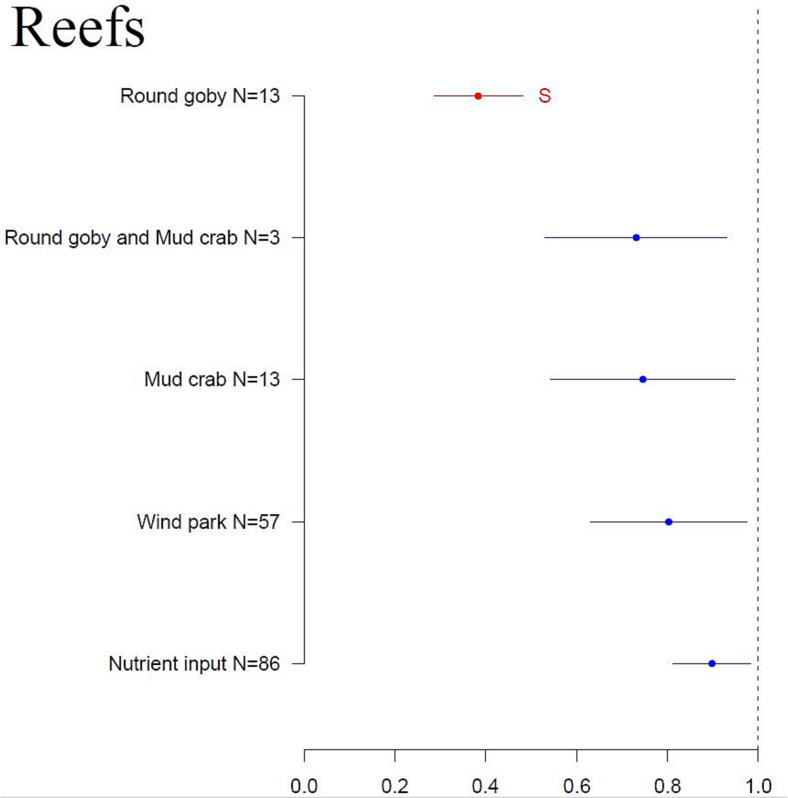
Results of the ANOVA analysis of the effect size (x-axis) of studied human pressures (y-axis) on the reef habitat. Effect size values < 1 indicate a habitat loss of reefs; values > 1 indicate an increase of reef habitat. Red indicates a significant change, blue shows a non-significant change. Primary data and the list of references are found in Supplementary material 1. (For interpretation of the references to color in this figure legend, the reader is referred to the web version of this article.)

The expected effect sizes of the human-induced pressures varied greatly among examined reef and sandbank habitats and associated biotopes (Fig. 3, Table 2). In general, the results indicate that human pressures are not expected to cause a significant change (*p* < 0.05) in the reefs, although the presence of round goby significantly (*p* < 0.001) reduced the areal extent (habitat coverage) of reefs. Other pressures also caused some habitat shrinkage. The lack of significant impacts stems from the large between-study variance in effect sizes. The effect size caused by round goby varied from 0.001 (i.e., almost complete destruction of habitat) to 0.999 (almost no impact), with the average value estimated at 0.384. When round goby and mud crab co-occur, their cumulative pressure is less damaging than the impact of round goby alone. Wind parks have no significant impact on reefs, but again, a large between-study variance in effect sizes was evident (from <0.001 to 5), with an average of 0.803. Similarly, the nutrient load had a broad range of effect sizes (from 0.029 to 5), with the mean value of 0.898, which is the highest impact value of the studied pressures. This represents the smallest negative impact among the pressures.

**Table 2:** One-way ANOVA analyses of the studied human pressures on the benthic habitats and associated biotopes. Significant effects are marked in bold.

Model	Type	Df	SS	MS	F-value	*P*-value
Reefs environment	Factor	4	3.097	0.774	0.825	0.511
	Residuals	167	156.714	0.938		
*Fucus* habitat	Factor	2	0.170	0.085	2.168	0.170
	Residuals	9	0.353	0.039		
*Furcellaria* habitat	Factor	1	0.814	0.814	38.992	**<0.001**
	Residuals	5	0.104	0.021		
*Mytilus* habitat	Factor	3	6.680	2.227	15.168	**<0.001**
	Residuals	8	1.174	0.147		
Sandbanks environment	Factor	4	55.62	13.904	6.723	**<0.001**
	Residuals	242	500.46	2.068		

**Fig. 4:**
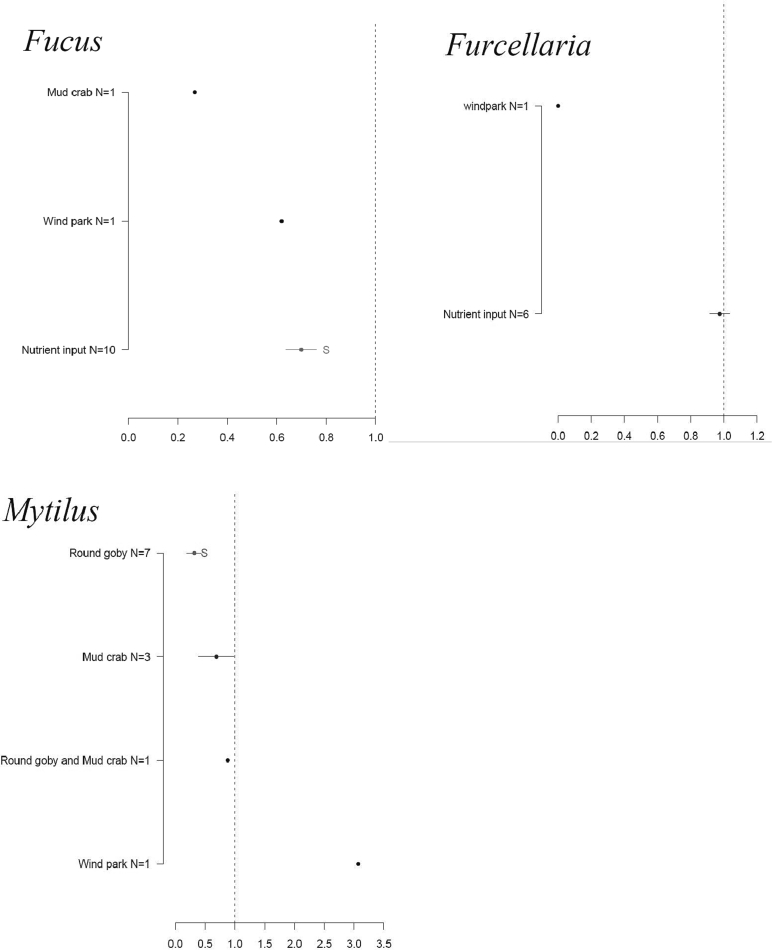
Results of the ANOVA analysis of the effect size (x-axis) of studied human-induced pressures (y-axis) on the *Fucus*, *Furcellaria*, and *Mytilus* biotopes. Effect size values < 1 indicate an areal loss; values > 1 indicate the increased area. Red indicates a significant change, blue shows a non-significant change, and black marks factors with only one data source available. Primary data and the list of references are found in Supplementary material 1. (For interpretation of the references to color in this figure legend, the reader is referred to the web version of this article.)

More significant impacts emerged in the species-specific biotopes (*Fucus vesiculosus*, *Furcellaria lumbricalis*, and *Mytilus trossulus*) associated with reefs, (Fig. 4, Table 2). However, a large within-group variance in effect sizes was evident for some combinations of human-induced pressures and biotopes, while other combinations completely lacked published data. The species-specific analysis showed that wind parks had a positive impact on mussel biotope and a negative impact on algae-based biotope. Round goby reduced the area of mussel and *Fucus* biotope; similar information on *Furcellaria* biotope is unavailable. Round goby had a strong negative impact (*p* = 0.001) on *Mytilus* biotope. Nutrient input caused a significant decline of the *Fucus* biotope (p = 0.001), but not for the *Furcellaria* biotope. The impact of nutrient input on mussels is unknown, indicating that more scientific research is needed.

#### 3.1.2 Sandbanks

**Fig. 5:**
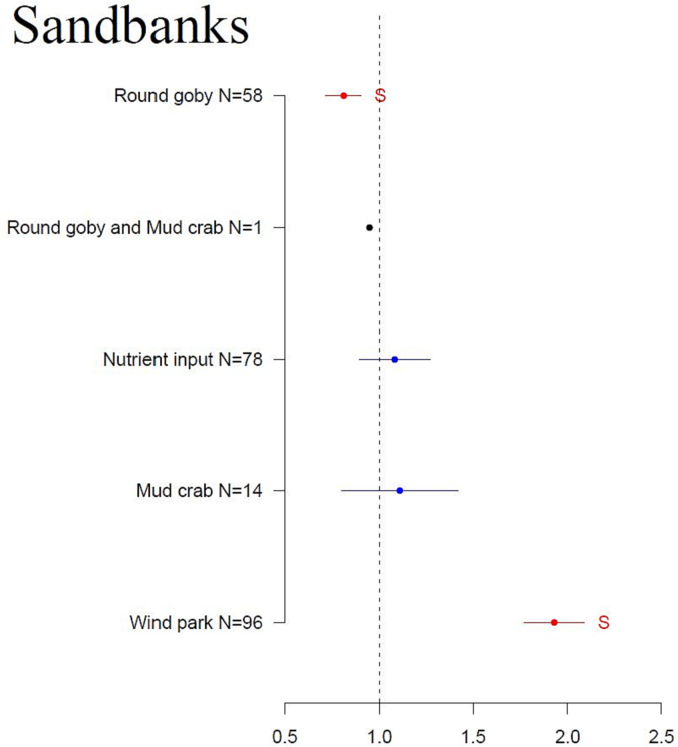
Results of the ANOVA analysis of the effect size (x-axis) of studied human pressures (y-axis) on the sandbank habitat. Effect size values < 1 indicate an areal loss of the habitat; values > 1 indicate an increase in sandbank area. Red indicates a significant change, blue shows a non-significant change, and black marks factors with only one data source available. Primary data and the list of references are found in Supplementary material 1. (For interpretation of the references to color in this figure legend, the reader is referred to the web version of this article.)

The studied human pressures had less impact on the sandbank habitat than the reef habitat (Fig. 5, Table 2). The presence of round goby had a strong (*p* = 0.04) negative impact on sandbanks, whereas wind parks had a strong positive impact (*p* < 0.001). Nutrient input and mud crab had a slightly positive impact on the sandbanks. There are insufficient data on the impact of the studied pressures on the species-specific sandbank habitats.

### 3.2 Scenario predictions

#### 3.2.1 Reef habitat and associated biotopes

The scenario-specific areal changes in all studied habitats/biotopes are presented in Supplementary material 2. All the tested scenarios predict some degree of habitat loss for reef habitat and its associated biotopes (except scenario 7, which predicts 13 % habitat gain for suspension feeders). The most severe negative impacts are caused by multiple current human pressures in the scenarios 5 and 6. Total habitat loss is greater for algae-based biotopes, indicating that *Fucus* and *Furcellaria* are more vulnerable to human impacts than reef habitat and suspension feeder biotopes. The least damaging impact scenarios on the studied benthic environments are future nutrient reduction (no habitat loss, scenario 2) and multiple human pressures (future nutrient reduction, wind parks, and nonnative species; scenario 7).

##### 3.2.1.1 Current nutrient load

The current nutrient load projects a 16 % total loss of reefs (Supplementary material 2, Fig. 1 (a); Table 3). More significant habitat losses are expected for both *Fucus* and *Furcellaria* biotopes, 62 % and 49 %, respectively (Supplementary material 2, Fig. 1 (b, c)). Suspension feeders are predicted to experience a small habitat gain (8 %), both in offshore waters and in the inner-coastal area between the islands and the mainland (Supplementary material 2, Fig. 1(d); Table 3).

**Table 3:** Predicted loss/gain of habitat cover per 1 km^2^ (%), changes in habitat cover (km^2^), and the predicted total loss/gain of the examined benthic ecosystems impacted by the tested scenarios (km^2^).

Benthic ecosystem	Predicted loss (−)/gain (+) of habitat per 1 km^2^ (%)		Changes in habitat areal cover (km^2^)					Predicted total loss/gain of habitat (%)
	Min	Max	Mean	The number of 1 km^2^ pixels including habitat	The number of 1 km^2^ pixels impacted by human activity	The total areal coverage of habitat	Predicted areal loss/gain of habitat	
1. Current nutrient load								
Reefs	−20	−20	−20	3180	2629	3180	−506	−16
*Fucus*	−90	−14	−34	1311	910	498	−309	−62
*Furcellaria*	−66	−16	−27	3359	2266	1263	−616	−49
Suspension feeders	+8	+20	+13.7	9728	1181	7780	+608	+8
Sandbanks	−17	−17	−17	2624	2179	2624	−353	−13
Charophytes	−85	−35	−62.4	1926	786	1390	−486	−35
*Zostera*	−81	−30	−45.7	305	305	162	−138	−85
Higher plants	−10	−3	−5.9	2697	1934	1453	−103	−7

2. Future nutrient reduction								
*Fucus*	−35	−14	−22.5	1311	10	498	−2	0
Sandbanks	−17	−17	−17	2624	24	2624	−4	0
Charophytes	−78	−38	−59.5	1926	30	1390	−18	−1
*Zostera*	−39	−34	−36.5	305	20	162	−1	0
Higher plants	−9	−5	−8.1	2697	37	1453	−3	0

3. Nonnative species								
Reefs	−64	−31	−63.7	3180	1330	3180	−973	−31
*Fucus*	−36	−2	−4.0	1311	1192	498	−65	−13
*Furcellaria*	−20	−2	−2.6	3359	2526	1263	−51	−4
Suspension feeders	−13	48	−3.2	9728	2259	7780	1024	+13
Sandbanks	−49	−6	−46.5	2624	2393	2624	−1240	−47
Charophytes	−31	−13	−22	1926	156	1390	−35	−3
*Zostera*	−5	−2	−3.1	305	305	162	−9	−6
Higher plants	−30	9	−7.1	2697	2047	1453	72	+5

4. Wind parks								
Suspension feeders	0	+1	+0.5	9728	784	7780	+1	0

5. Current nutrient load + nonnative species								
Reefs	−64	−20	−31.4	3180	3102	3180	−1079	−34
*Fucus*	−90	−2	−26.1	1311	1285	498	−343	−69
*Furcellaria*	−2	−66	−20.8	3359	3154	1263	−639	−51
Suspension feeders	−40	+48	−5.0	9728	+4665	7780	+891	+11
Sandbanks	−49	−6	−38.5	2624	2553	2624	−1107	−42
Charophytes	−85	−13	−57.9	1926	887	1390	−509	−37
Zostera	−81	−30	−45.7	305	305	162	−139	−86
Higher plants	−30	9	−4.7	2697	1593	1453	−103	−7

6. Current nutrient load + nonnative species + wind parks								
Reefs	−64	−20	−31.3	3180	3089	3180	−1079	−34
*Fucus*	−90	−2	−26.1	1311	1285	498	−343	−69
*Furcellaria*	−2	−66	−20.8	3359	3154	1263	−639	−51
Suspension feeders	−40	+48	−2.7	9728	+4465	7780	+883	+11
Sandbanks	−49	−6	−38.5	2624	2553	2624	−1107	−42
Charophytes	−85	−13	−57.9	1926	887	1390	−509	−37
Zostera	−81	−30	−45.7	305	305	162	−139	−86
Higher plants	−30	9	−6.8	2697	1593	1453	−103	−7

7. Future nutrient reduction + nonnative species + wind parks								
Reefs	−64	−31	−63.7	3180	1319	3180	−973	−31
*Fucus*	−36	−2	−5.0	1311	1192	498	−67	−13
*Furcellaria*	−20	−2	−2.6	3359	2526	1263	−51	−4
Suspension feeders	−40	−48	+22.7	9728	2898	7780	+996	+13
Sandbanks	−49	−6	−46.4	2624	2393	2624	−1236	−47
Charophytes	−78	−13	−28.8	1926	186	1390	−52	−4
Zostera	−39	−2	−3.3	305	305	162	−9	−6
Higher plants	−30	9	+7.1	2697	+1973	1453	+66	+5

##### 3.2.1.2 Future nutrient reduction (HELCOM MAI target)

The scenario of the MAI target of 25 % reduction in nutrient input predicts that most areal cover in the reef habitat and associated biotopes are not impacted by nutrient load and will not experience any substantial habitat change. The only location where habitat loss is projected is near Haapsalu Bay on the inner-coastal shallow waters adjacent to the populated town of Haapsalu (Supplementary material 2, Fig. 2). Only 2 km^2^ of the total *Fucus* biotope is lost, a loss that does not affect the total *Fucus* areal cover.

##### 3.2.1.3 The presence of nonnative species (round goby and mud crab)

Reefs are predicted to lose 31 % of its habitat cover due to the presence of round goby and mud crab (Supplementary material 2; Fig. 3 (a), Table 3). The number of 1 km^2^ sites impacted by alien species is great for *Fucus* (1192 km^2^) and *Furcellaria* (2526 km^2^) biotopes; however, the average impact percentage is low: −4 % and −3 %, respectively (Table 3). Therefore, this pressure does not cause a significant total habitat loss for either: 13 % in *Fucus* and 4 % in *Furcellaria* (Supplementary material 2, Fig. 3 (b, c); Table 3). Suspension feeders biotope is projected to increase by 13 % (Supplementary material 2, Fig. 3 (d); Table 3).

##### 3.2.1.4 Projected wind parks (according to the Estonian maritime spatial plan)

The predicted impact of wind parks is orders of magnitude less than other pressures because the foundations of wind turbines cover only 1 % of the seafloor. Only a total of 1 km^2^ of new suspension feeder biotope is predicted to be created; however, this does not affect the total habitat cover (Table 3). The areas that overlap with projected wind park sites and have a naturally higher abundance of *Mytilus* experience this slight impact (Supplementary material 2, Fig. 4). The impact on reefs and algae-based associated biotopes is negligible, <0.1 %.

##### 3.2.1.5 Current nutrient load + nonnative species

This scenario predicts a 34 % loss of reef habitat due to the combination of current nutrient load and nonnative species (Supplementary material 2, Fig. 5 (a); Table 3). *Fucus* and *Furcellaria* biotopes are both susceptible to these combined pressures, indicating that more than half of both biotopes will be lost: 69 % and 51 %, respectively (Supplementary material 2, Fig. 5 (b, c); Table 3). The suspension feeder biotope is predicted to increase by 11 % (Supplementary material 2, Fig. 5 (d); Table 3).

##### 3.2.1.6 Current nutrient load + nonnative species + wind parks

Since the projected wind park sites do not significantly impact reefs or associated biotopes, these results are almost identical to scenario 5. The mean percentages of habitat loss per 1 km^2^ in reef habitats and suspension feeder biotopes are slightly less than in scenario 5 (Supplementary material 2, Fig. 6 (a–d); Table 3).

##### 3.2.1.7 Future nutrient reduction + nonnative species + wind parks

**Fig. 6:**
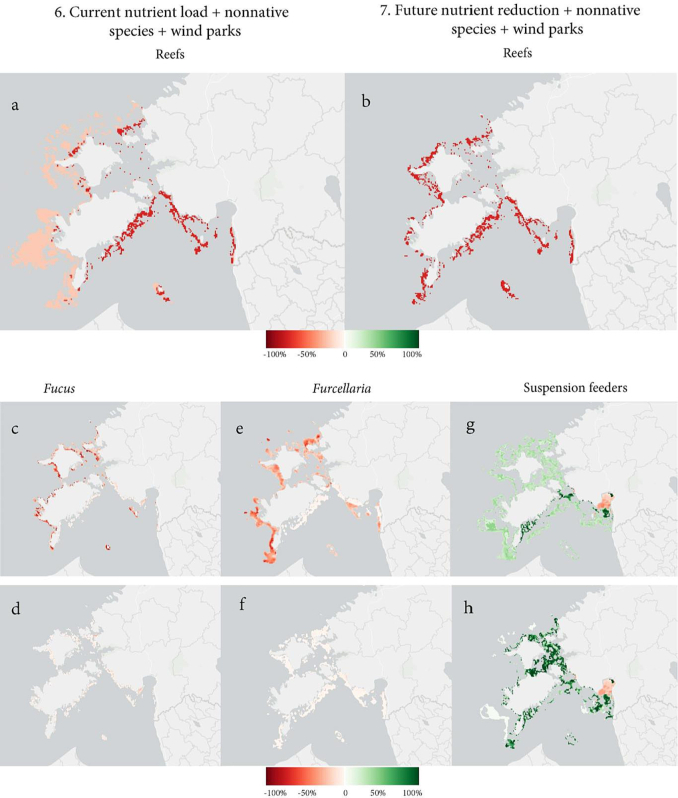
Habitat change comparing the combinations of current nutrient load + nonnative species + wind park development versus 25 % nutrient reduction + nonnative species + wind park development scenarios. Maps (a, b) show the difference in larger-scale reef habitat. Differences in habitat change in associated biotopes are (c, d) in *Fucus*, (e, f) in *Furcellaria*, and (g, h) in suspension feeders (percentage change in km^2^ in a 1 km^2^ cell).

Among the combined human pressures, the reduction of nutrient input by 25 % is the scenario with the greatest positive impact on the reef and associated biotopes; yet still projecting a 31 % decline in reefs (Supplementary material 2, Fig. 7 (a); Table 3). The habitat loss caused by round goby and mud crab remains. The results are also substantial for the associated habitat types, especially when compared to the areas impacted by scenario number 6. In total, *Fucus* is predicted to experience an average of 13 % habitat loss, *Furcellaria* only 4 % loss, and suspension feeder is predicted to increase in inshore waters; the total habitat gained is 13 % (Fig. 6).

#### 3.2.2 Sandbank habitat and associated biotopes

Overall, the tested scenarios predict habitat loss for the sandbank habitat and associated biotopes, the exception being the higher plants biotope, in which scenarios 3 and 7 predict habitat gain. Like reefs, the most substantial negative impacts are caused by combined current human pressures in scenarios 5 and 6, and associated biotopes are more sensitive than the sandbank habitat as a whole. The greatest habitat degradation is experienced in the *Zostera* biotope (up to 86 %, however; this biotope is especially sensitive to excessive nutrient input as habitat loss is significantly less when the nutrient load is reduced (Table 3). Therefore, the least damaging impact scenario of all the studied benthic environments with no or minor habitat loss is future nutrient reduction (scenario 2) and the future nutrient reduction combined with other pressures (scenario 7).

##### 3.2.2.1 Current nutrient load

The current nutrient load scenario projects a 13 % loss of the sandbank habitat (Supplementary material 2, Fig. 8 (a); Table 3). The results vary more in associated habitats: higher plants are projected to experience a minor habitat loss (7 %); (Supplementary material 2, Fig. 8 (d). More substantial loss is expected in the Charophytes biotope (35 %); (Supplementary material 2, Fig. 8 (b); Table 3). *Zostera* habitat is extremely sensitive to the current nutrient inflow, experiencing an 85 % habitat decline (Supplementary material 2, Fig.8 (c); Table 3).

##### 3.2.2.2 Future nutrient reduction (HELCOM MAI target)

The MAI target of 25 % reduction in nutrient input scenario predicts slight losses in areal cover: 0.7 km^2^ in Zostera, 3 km^2^ in higher plants, 4 km^2^ in sandbanks, and 18 km^2^ in Charophytes (Supplementary material 2, Fig. 9; Table 3). However, these areal losses are too low to impact the associated biotopes significantly (1 % total loss of Charophytes; 0 % for the other biotopes). This scenario shows that nutrient reduction is vital to protect *Zostera* biotope as there is no habitat loss predicted with this scenario.

##### 3.2.2.3 The presence of nonnative species (round goby and mud crab)

The sandbank habitat is predicted to lose almost half of its habitat cover (47 %) due to round goby and mud crab (Supplementary material 2, Fig. 10 (a); Table 3). The associated biotopes are not strongly impacted: Charophytes are predicted to lose a total of 3 % and Zostera 6 % of its habitat (Supplementary material 2, Fig. 10 (b, c); Table 3). The habitat cover for higher plants increases by 5 % (Supplementary material 2, Fig. 10 (d); Table 3).

##### 3.2.2.4 Projected wind parks (according to the Estonian maritime spatial plan)

The sandbank habitat overlaps with projected wind park sites in 7 km^2^. Habitat coverage is already at its maximum 100 %; no habitat gain is possible in this area.

##### 3.2.2.5 Current nutrient load + nonnative species

The combination of current nutrient load and nonnative species has a severe negative impact on all the studied benthic environments. The results indicate 42 % habitat for sandbanks (Supplementary material 2, Fig. 11 (a); Table 3). As in scenario 1, the results vary within associated biotopes: higher plants are projected to experience a 7 % loss, whereas 37 % loss is projected for the Charophytes and 86 % decline for the *Zostera* (Supplementary material 2, Fig. 11 (b–d); Table 3).

##### 3.2.2.6 Current nutrient load + nonnative species + wind parks

Because the projected wind park sites have no significant impact on the sandbank or associated biotopes, these results are almost identical to scenario 5. The mean percentage of habitat loss per 1 km^2^ in higher plants biotope is slightly greater than scenario 5 (Supplementary material 2, Fig. 12 (d); Table 3).

##### 3.2.2.7 Future nutrient reduction + nonnative species + wind parks

**Fig. 7:**
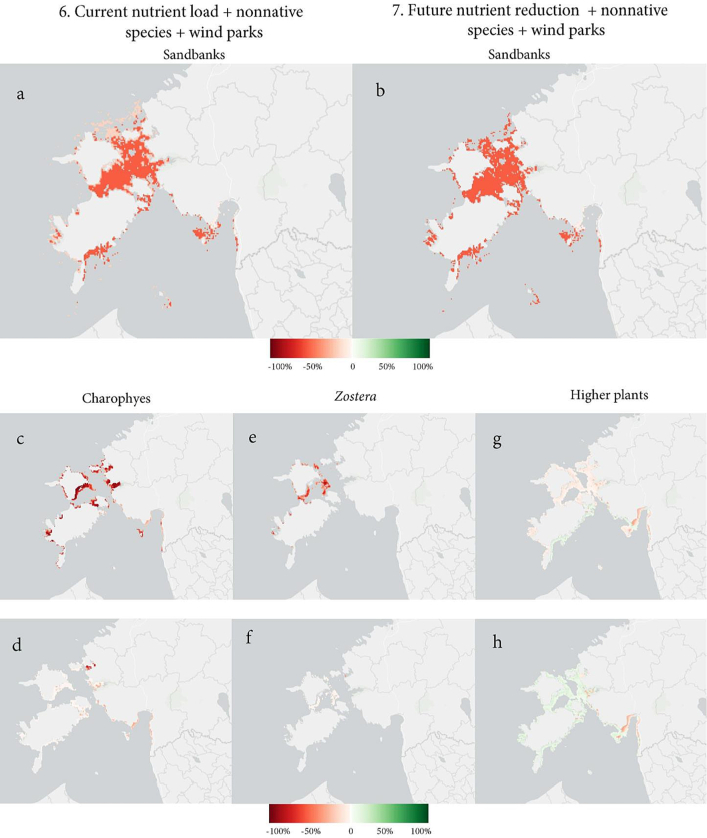
Habitat change comparing the combinations of current nutrient load + nonnative species + wind park development versus 25 % nutrient reduction + nonnative species + wind park development scenarios. Maps (a, b) show the difference in a larger-scale sandbank habitat. Differences in habitat change in associated biotopes are (c, d) in Charophytes, (e, f) in *Zostera*, and (g, h) in higher plants (percentage change in km^2^ in a 1 km^2^ cell).

Reducing nutrient input by 25 % indicates a difference between the sandbank and associated biotopes: whereas a 47 % habitat loss is predicted for sandbanks (Supplementary material 2, Fig. 13 (a)); Table 3), only a 4 % and 6 % loss is predicted for the Charophytes and *Zostera* biotopes, respectively (Supplementary material 2, Fig. 13 (b, c); Table 3). The total habitat for higher plants will increase by 5 % (Supplementary material 2, Fig. 13 (d); Table 3). Therefore, sandbanks are more sensitive to the presence of alien species, whereas smaller associated biotopes are most impacted by excessive nutrient input. A comparison of the results between scenarios 6 and 7 shows the positive impact of a 25 % nutrient reduction (Fig. 7).

#### 3.2.3 Variability of environmental responses to pressures

To add more insight on the variability of environmental responses to the studied pressures, the PW4B portal generates impact maps that use mean effects together with their 95 % confidence intervals to calculate the expected maximum, mean, and minimum predicted habitat cover for each studied scenario.

**Fig. 8:**
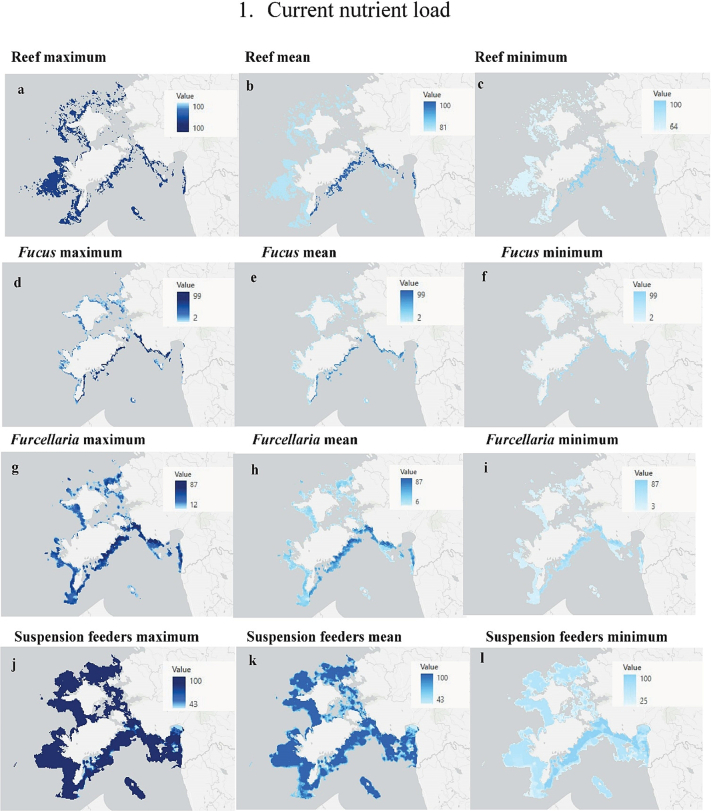
The predicted maximum, mean, and minimum areal cover of Reef habitat (a–c), *Fucus* biotope (d–f), *Furcellaria* biotope (g–i), Suspension feeders biotope (j–l) in result of the impact of the Current nutrient load scenario (Scenario 1). Unit 100 denotes 100 % coverage (i.e. 1 km^2^ cover of habitat/biotope) in a 1 km^2^ grid cell.

Here, we exemplify this with the Current nutrient load scenario (Scenario 1), reef habitat and associated biotopes (Fig. 8). As seen in the Subsection 3.1, the human-induced pressures result in effects on examined reef and associated biotopes which have large natural variability due to a large variability in effect sizes among primary studies (Fig. 3, Fig. 4). Consequently, predicted habitat cover per 1 km^2^ in reef habitat and associated biotopes also vary to a large extent. Nevertheless, meta-analytic technique, which was used to summarize all evidence, was able to reduce high natural variability of primary studies and provide meaningful and robust assessment. For the maps of other scenarios see the PW4B portal.

## 4 Discussion

We examined in this study the impacts of individual and multiple human activities (nutrient input, invasive species, and wind parks) on various benthic habitats and associated biotopes in the Baltic Sea region. In general, an excessive nutrient load is expected to damage benthic environments more than any other human pressures. This effect was observed even without the presence of other pressures. Elevated nutrient loading only favored suspension feeding mussels. Mussels inhabiting the study area are filter feeders, and elevated nutrient loading is expected to improve their food availability. Resource gradients have an important role in shaping the biomass distribution of mussels in the study area (Kotta et al., 2015).

This study analyzed benthic habitats separately and did not integrate different habitats into a single entity. The rationale of this decision is that the studied habitats do not overlap spatially in our study area and the response to different pressures is expected to be habitat specific. Had the analysis not been habitat-specific or biotope-specific, the analysis would likely have erroneously demonstrated lower cumulative impacts than observed. This is largely because data aggregation in ecological meta-analysis typically results in the underestimation of impacts, especially when averaged effect sizes amalgamate many opposing ecological processes (Thomsen, 2020). This results in situations where the reported aggregated weak impacts (not supported by the primary literature) suggest that marine ecosystems are little affected by the studied human pressures, which in turn would understate the need for management of these human pressures. As a result, the more detailed habitat distinction we used generates more accurate assessments of the potential impacts of human activities. This habitat distinction also allows planners and environmental managers to seek more appropriate action to overcome environmental challenges as the efficiency of different measures is highly habitat- and biotope-dependent.

The advantage of this spatial arrangement is supported by our empirical evidence. The impacts of human pressures in this study were often greater at biotope scale than at habitat scale. For example, in scenario 1 (current nutrient loading) reefs and sandbanks were characterized by lower negative impact (16 % and 13 % loss, respectively) than their associated biotopes (85 % in *Zostera* habitat, 62 % in *Fucus*, 49 % in *Furcellaria*, and 35 % in Charophytes habitats). However, higher plants are projected to experience a 7 % loss and suspension feeders an 8 % gain, indicating that these biotopes are less sensitive to nutrient load than any other studied biotopes. Consequently, it is advantageous to assess the impacts of human-induced pressures at a smaller-scale biotope level rather than at habitat level to ensure more accurate and effective marine conservation assessment. Otherwise, we may overlook important impacts caused by human-induced pressures that lead to underestimating species response. In our study, many impact values were not statistically significant due to large between-study variance in effect sizes. For instance, the impacts of wind parks on different reef biotopes varied widely with wind parks increasing *Mytilus* biotopes more than three-fold, while decreasing algae cover in *Fucus* and *Furcellaria* biotopes (Fig. 3). Similar differences in responses were also reflected in terms of spatial loss/gain in the prediction maps (Supplementary material 2).

Wind park development exhibited the lowest impacts on the studied benthic habitats with sandbank being the only habitat with a statistically significant effect (*p* < 0.001, mean impact value = 2). This potentially reflects a spillover effect from reefs as wind park foundations create new substrates in the sandbank habitat, increasing the areal cover in a soft bottom habitat where mussels can attach. However, this did not correspond with the results seen in the predicted changes in habitat cover since projected wind parks provide little new habitat in areas fully covered by sandbanks. The predicted maps showed that wind parks predict only a small positive impact on suspension feeders, creating 1 km^2^ of new habitat. Therefore, wind parks are expected to increase the areal coverage of reef and sandbank habitats by a minimal amount, as the foundations of wind turbines create a stable artificial substrate for *Mytilus trossulus*. Nevertheless, as a result of elevated densities of filter-feeding mussels, wind parks are expected to mitigate adverse impacts of eutrophication since filter feeders remove nutrients from water (Kotta et al., 2009). This is, however, not the case for macro-algae biotopes, as the existing evidence show a mean effect size value < 1, meaning that wind parks will most likely cause areal loss for macro-algae.

Our analyses indicate that a business-as-usual scenario will cause permanent losses in benthic habitats and associated biotopes due to the combined adverse impacts of excessive nutrient input and nonnative species in both soft and hard bottoms. A combination of current nutrient load and the presence of nonnative species predicts a total loss of 86 % of eelgrass (*Zostera*) biotope, 69 % of *Fucus*, 51 % of *Furcellaria*, and 37 % of Charophytes biotope. Both reefs and sandbanks are considered hotspots for biodiversity in the Baltic Sea that require strict conservation measures as the modification or loss of habitats can pose a serious threat to marine ecosystems. Around 90 % of both habitats (3180 km^2^ of reefs and 2624 km^2^ of sandbanks) are located in our study area, making it a valuable and important location for research on benthic habitats and associated biotopes in Estonian waters. The number of habitat-forming species in the Baltic Sea is relatively low; therefore, few alternative species are available to replace the function of species that might disappear due to the habitat decline.

Nutrient load is a manageable pressure that can and should be reduced. The results showed that reducing nutrient load by 25 % together with the presence of nonnative species and projected wind parks is a significant improvement for the marine environment: a total loss of 13 % in *Fucus*, 6 % in *Zostera* biotopes, 4 % in *Furcellaria*, and Charophytes biotopes, 5 % gain in higher plants, and 13 % gain in mussel biotopes. Based on our research, it is therefore highly encouraged for Estonia to follow the HELCOM MAI targets to conserve valuable marine environments.

Uncertainty is inherent in any modelling approach as in the cumulative impact analyses. A cumulative impact assessment includes several sources and causes of uncertainty, and these should be clearly communicated to practitioners to facilitate the correct interpretation of the analysis results (Gissi et al., 2017). Uncertainties due to modelling of benthic habitats and biotopes have been discussed in Aps et al. (2018) and Torn et al. (2020). Acknowledging the relatively data rich situation of the Estonian coastal sea, the habitat and associated biotope maps explain about 85–95 % of natural variability and thereby this source of uncertainty is of little consequence in our case study.

Another important source of uncertainty in cumulative impact assessments relates to sensitivity scores. Sensitivity scores define responses of a habitat (or associated biotope) to any combinations of pressures caused by different human activity. Previous studies have mostly constructed sensitivity scores based on sensitivity analyses from expert judgement (Jones et al., 2018) because empirical evidence is difficult to compile as information is distributed among various databases and literature source. Although expert driven scores provide seemingly smaller uncertainty bounds and thereby lead to more constrained assessment results (Gissi et al., 2017; Jones et al., 2018), these scores are mostly not validated using the “correct” sensitivity score for each habitat/biotope to different pressures in real-life scenarios.

In last decades as the pace of scientific research increases so does the accumulated experimental and observational evidence on the impacts of different human activities on different habitats and biotopes. This suggests that cumulative impact assessments should be based on a plethora of scientific information that is expanding, can be systematically searched and compiled into data-driven sensitivity scores. A data-driven approach is transparent, verifiable and can resolve complex interactions between multiple pressures that are currently overlooked by experts (Piggott et al., 2015).

Our approach used the existing published evidence that indicated individual and/or combined impacts of the studied human-induced pressures, followed by incorporation of these impacts into spatial predictions within different benthic habitats as a cumulative impact assessment (e.g., Kotta et al., 2020). The model predicted mean impacts together with estimates of their 95 % confidence intervals. Our analysis clearly demonstrated a large variability in effect sizes among primary studies; nevertheless, meta-analytic technique, which was used to summarize all evidence, was able to accommodate high natural variability and provide meaningful and robust sensitivity scores. Large uncertainty tends to arise when there are few studies and these studies give dissimilar results. This situation provides insight into the needs for additional empirical studies to target gaps in knowledge. In addition, a large natural variability in response to human activity is expected along important environmental gradients. This gap in knowledge is more difficult to overcome as it demands too many additional empirical studies over a large number of important environmental gradients. Taking this into consideration, the presentation of uncertainties carries two important messages: (1) when a practitioner is about to make a cumulative impact assessment, greater weight should be given to those results with smaller margins of error; (2) for combinations of human activities where the margins of error are very large, specific studies (preferably in the same area for which planning is being done) are needed to mitigate the risks of large uncertainty. Unlike data-driven analysis, expert judgement is heavily dependent on the expert's background knowledge. In most cases, expert opinion cannot reproduce all available scientific literature on human impacts, nor can an expert quantify the uncertainty of opinion as well as in a meta-analytic framework. Therefore, a data-based cumulative impact estimate subject to high variability (mostly due to large natural variability in response to human activity) remains superior to expert judgement, for which a true quantitative margin of error is unattainable.

Evidence-based data are key for developing effective decision support tools. Although all publicly available evidence was included in the PW4B tool, the database still lacks some human-pressure and species-specific information. Thus, additional basic ecological studies should be initialized to gather more data on these valuable habitats and species. In cases where the habitat or biotope-specific information was absent, we incorporated expert knowledge in the PW4B tool. Although impact coefficients of some combinations of pressures still rely on expert judgement rather than empirical data, the PW4B tool will incorporate more objective input in the future as new data become available (Kotta et al., 2020). Not enough data are available to see how human impacts affect specific biotopes in the sandbank habitat, such as *Zostera*, infauna, higher plants, and Charophytes biotopes.

MSP is expected to apply an ecosystem-based approach to ensure that the collective pressure of human activities is kept within acceptable limits in the marine realm. Cumulative impact assessment is increasingly being used to evaluate the impact of multiple pressures on marine ecosystems in MSP (e.g. Gissi et al., 2017; Jones et al., 2018) and here DSTs are considered a valuable instrument to reduce the inherent complexities of ecosystem for environmental managers and conservation practitioners with the aim to decrease vulnerability and increase resilience in natural systems (Depellegrin et al., 2021). Some of the existing DSTs include elements of cumulative impacts assessment to support ecosystem-based management from national to macro-regional scales and thereby effectively facilitating communication at the science-policy interface (Depellegrin et al., 2021). These tools mostly apply a widely accepted approach of cumulative impact assessment (Halpern et al., 2008) without fully explaining or exploring limitations of assumption behind the analysis. For example, habitats are assessed either existing or absent in a pixel rather than using the probability of occurrence or cover of habitats (Halpern and Fujita, 2013). The tools also assume individual and linear response of ecosystems to stressors, whereas most marine areas are impacted by multiple concurrent stressors, which rarely act in isolation but instead produce interactive impacts on multiple nature values (e.g. Stockbridge et al., 2020). Finally, these tools rarely use empirical data to define response functions, but instead rely on expert judgement. Most of these shortcomings were necessary owing to data limitations, but with the impressive emergence of new data and knowledge in recent decades, a commonly agreed approach of cumulative impact assessment should be revised.

PW4B is the first data-driven DST on cumulative impact analysis to take advantage of the accumulated knowledge of scientific evidence on the individual and/or combined impacts of human-induced pressures on habitats and associated biotopes. As such, it helps to bridge the gap between science and environmental managers to find the most environmentally-friendly planning solutions. PW4B is a helpful tool in MSP to run complex analyses, but it also provides output in simple formats that are accessible for diverse stakeholders. PW4B is convenient for policymakers to explore plausible future management scenarios of impacts of individual or combined human pressures. To illustrate this, we included the projected offshore wind park sites in our research, as it is currently a nonexistent pressure with unknown long-term consequences on marine ecosystems. DSTs are useful for the stakeholders to fill this knowledge gap by estimating the potential impacts of selected pressure combinations on specific locations. In terms of wind park development, PW4B includes the most current and thorough knowledge on the patterns of habitats (predictive maps) as well as an algorithm on the plausible consequence of wind parks on the biota (extracted numerically from the literature and databases). Moreover, we can examine both individual and combined impacts of wind parks in Estonian waters and eventually predict the most suitable sites for sustainable marine development.

## CRediT authorship contribution statement

**Annaleena Vaher:** Conceptualization, Methodology, Formal analysis, Investigation, Writing – original draft, Writing – review & editing, Visualization, Resources. **Jonne Kotta:** Conceptualization, Methodology, Software, Formal analysis, Investigation, Writing – original draft, Writing – review & editing, Visualization, Validation, Data curation, Supervision, Project administration, Funding acquisition. **Robert Szava-Kovats:** Writing – review & editing, Validation, Resources. **Ants Kaasik:** Software, Data curation. **Mihhail Fetissov:** Software, Data curation. **Robert Aps:** Methodology, Supervision. **Anneliis Kõivupuu:** Methodology, Data curation, Supervision.

## Declaration of competing interest

The authors declare that they have no known competing financial interests or personal relationships that could have appeared to influence the work reported in this paper.

## Data Availability

Data will be made available on request.

## Appendix A Supplementary data

Supplementary material 1: Metadata table Image 1

Supplementary material 2: Prediction maps Image 2
