# Supplementary material for: Assessing cumulative impacts of human-induced pressures on reef and sandbank habitats and associated biotopes in the northeastern Baltic Sea
Source: Mar Pollut Bull. 2022 Oct;183:114042. doi: 10.1016/j.marpolbul.2022.114042 (PMC9551195; doi:10.1016/j.marpolbul.2022.114042)
Supplement: Supplementary file 1 — Supplementary material 1: Metadata table [file mmc1.pdf]

Supplementary material 1

Reference	Human activity	Habitat name	Biotope name	Effect size
Nurkse, K., Kotta, J., Rätsep, M., Kotta, I., Kreitsberg, R., 2018. Experimental evaluation of the effects of the novel predators, round goby and mud crab on benthic invertebrates in the Gulf of Riga, Baltic Sea. J. Mar. Biol. Assoc. U. K. 98, 25–31.	Round goby	Reefs		0.961
Nurkse, K., Kotta, J., Rätsep, M., Kotta, I., Kreitsberg, R., 2018. Experimental evaluation of the effects of the novel predators, round goby and mud crab on benthic invertebrates in the Gulf of Riga, Baltic Sea. J. Mar. Biol. Assoc. U. K. 98, 25–31.	Round goby	Reefs		0.391
Estonian Marine Institute database	Round goby	Reefs		0.393
European Invasive Species impact database, No 64; Nurkse, K., Kotta, J., Orav-Kotta, H., Ojaveer, H. (2016) A successful non-native predator, round goby, in the Baltic Sea: generalist feeding strategy, diverse diet and high prey consumption. Hydrobiologia 777, 271–281.	Round goby	Reefs		0.013
European Invasive Species impact database, No 65; Nurkse, K., Kotta, J., Orav-Kotta, H., Ojaveer, H. (2016) A successful non-native predator, round goby, in the Baltic Sea: generalist feeding strategy, diverse diet and high prey consumption. Hydrobiologia 777, 271–281.	Round goby	Reefs		0.013
European Invasive Species impact database, No 66; Nurkse, K., Kotta, J., Orav-Kotta, H., Ojaveer, H. (2016) A successful non-native predator, round goby, in the Baltic Sea: generalist feeding strategy, diverse diet and high prey consumption. Hydrobiologia 777, 271–281.	Round goby	Reefs		0.752
Nurkse, K., Kotta, J., Orav-Kotta, H., Ojaveer, H. (2016) A successful non-native predator, round goby, in the Baltic Sea: generalist feeding strategy, diverse diet and high prey consumption. Hydrobiologia 777, 271–281.	Round goby	Reefs		0.341
Nurkse, K., Kotta, J., Orav-Kotta, H., Ojaveer, H. (2016) A successful non-native predator, round goby, in the Baltic Sea: generalist feeding strategy, diverse diet and high prey consumption. Hydrobiologia 777, 271–281.	Round goby	Reefs		0.223
European Invasive Species impact database, No 68; Skabeikis, A., Morkūnė, R., Bacevičius, E., Lesutienė, J., Morkūnas, J., Poškienė, A., & Šiaulys, A. (2019). Effect of round goby (Neogobius melanostomus) invasion on blue mussel (Mytilus edulis trossulus) population and winter diet of the long-tailed duck (Clangula hyemalis). Biological Invasions, 21(3), 911-923.	Round goby	Reefs		0.000
European Invasive Species impact database, No 69; Skabeikis, A., Morkūnė, R., Bacevičius, E., Lesutienė, J., Morkūnas, J., Poškienė, A., & Šiaulys, A. (2019). Effect of round goby (Neogobius melanostomus) invasion on blue mussel (Mytilus edulis trossulus) population and winter diet of the long-tailed duck (Clangula hyemalis). Biological Invasions, 21(3), 911-923.	Round goby	Reefs		0.013
European Invasive Species impact database, No 70; Skabeikis, A., Morkūnė, R., Bacevičius, E., Lesutienė, J., Morkūnas, J., Poškienė, A., & Šiaulys, A. (2019). Effect of round goby (Neogobius melanostomus) invasion on blue mussel (Mytilus edulis trossulus) population and winter diet of the long-tailed duck (Clangula hyemalis). Biological Invasions, 21(3), 911-923.	Round goby	Reefs		0.286
Nurkse, K., Kotta, J., Rätsep, M., Kotta, I., Kreitsberg, R., 2018. Experimental evaluation of the effects of the novel predators, round goby and mud crab on benthic invertebrates in the Gulf of Riga, Baltic Sea. J. Mar. Biol. Assoc. U. K. 98, 25–31.	Round goby	Reefs		0.999
Nurkse, K., Kotta, J., Rätsep, M., Kotta, I., Kreitsberg, R., 2018. Experimental evaluation of the effects of the novel predators, round goby and mud crab on benthic invertebrates in the Gulf of Riga, Baltic Sea. J. Mar. Biol. Assoc. U. K. 98, 25–31.	Rround goby and Mud crab	Reefs		0.881
Nurkse, K., Kotta, J., Rätsep, M., Kotta, I., Kreitsberg, R., 2018. Experimental evaluation of the effects of the novel predators, round goby and mud crab on benthic invertebrates in the Gulf of Riga, Baltic Sea. J. Mar. Biol. Assoc. U. K. 98, 25–31.	Rround goby and Mud crab	Reefs		0.337
Nurkse, K., Kotta, J., Rätsep, M., Kotta, I., Kreitsberg, R., 2018. Experimental evaluation of the effects of the novel predators, round goby and mud crab on benthic invertebrates in the Gulf of Riga, Baltic Sea. J. Mar. Biol. Assoc. U. K. 98, 25–31.	Rround goby and Mud crab	Reefs		0.975
Nurkse, K., Kotta, J., Rätsep, M., Kotta, I., Kreitsberg, R., 2018. Experimental evaluation of the effects of the novel predators, round goby and mud crab on benthic invertebrates in the Gulf of Riga, Baltic Sea. J. Mar. Biol. Assoc. U. K. 98, 25–31.	Mud crab	Reefs		0.980
Nurkse, K., Kotta, J., Rätsep, M., Kotta, I., Kreitsberg, R., 2018. Experimental evaluation of the effects of the novel predators, round goby and mud crab on benthic invertebrates in the Gulf of Riga, Baltic Sea. J. Mar. Biol. Assoc. U. K. 98, 25–31.	Mud crab	Reefs		0.788
Forsström, T., Fowler, A. E., Manninen, I., & Vesakoski, O. (2015). An introduced species meets the local fauna: predatory behavior of the crab Rhithropanopeus harrisii in the Northern Baltic Sea. Biological invasions, 17(9), 2729-2741.	Mud crab	Reefs		0.081
Forsström, T., Fowler, A. E., Manninen, I., & Vesakoski, O. (2015). An introduced species meets the local fauna: predatory behavior of the crab Rhithropanopeus harrisii in the Northern Baltic Sea. Biological invasions, 17(9), 2729-2741.	Mud crab	Reefs		0.124

Forsström, T., Fowler, A. E., Manninen, I., & Vesakoski, O. (2015). An introduced species meets the local fauna: predatory behavior of the crab Rhithropanopeus harrisii in the Northern Baltic Sea. Biological invasions, 17(9), 2729-2741.	Mud crab	Reefs		0.481
Jormalainen, V., Gagnon, K., Sjöroos, J., & Rothäusler, E. (2016). The invasive mud crab enforces a major shift in a rocky littoral invertebrate community of the Baltic Sea. Biological invasions, 18(5), 1409-1419.	Mud crab	Reefs		0.012
Jormalainen, V., Gagnon, K., Sjöroos, J., & Rothäusler, E. (2016). The invasive mud crab enforces a major shift in a rocky littoral invertebrate community of the Baltic Sea. Biological invasions, 18(5), 1409-1419.	Mud crab	Reefs		0.960
Jormalainen, V., Gagnon, K., Sjöroos, J., & Rothäusler, E. (2016). The invasive mud crab enforces a major shift in a rocky littoral invertebrate community of the Baltic Sea. Biological invasions, 18(5), 1409-1419.	Mud crab	Reefs		2.836
Jormalainen, V., Gagnon, K., Sjöroos, J., & Rothäusler, E. (2016). The invasive mud crab enforces a major shift in a rocky littoral invertebrate community of the Baltic Sea. Biological invasions, 18(5), 1409-1419.	Mud crab	Reefs		0.323
Jormalainen, V., Gagnon, K., Sjöroos, J., & Rothäusler, E. (2016). The invasive mud crab enforces a major shift in a rocky littoral invertebrate community of the Baltic Sea. Biological invasions, 18(5), 1409-1419.	Mud crab	Reefs		1.000
Jormalainen, V., Gagnon, K., Sjöroos, J., & Rothäusler, E. (2016). The invasive mud crab enforces a major shift in a rocky littoral invertebrate community of the Baltic Sea. Biological invasions, 18(5), 1409-1419.	Mud crab	Reefs		0.891
Jormalainen, V., Gagnon, K., Sjöroos, J., & Rothäusler, E. (2016). The invasive mud crab enforces a major shift in a rocky littoral invertebrate community of the Baltic Sea. Biological invasions, 18(5), 1409-1419.	Mud crab	Reefs		0.268
Nurkse, K., Kotta, J., Rätsep, M., Kotta, I., Kreitsberg, R., 2018. Experimental evaluation of the effects of the novel predators, round goby and mud crab on benthic invertebrates in the Gulf of Riga, Baltic Sea. J. Mar. Biol. Assoc. U. K. 98, 25–31.	Mud crab	Reefs		0.951
Wilhelmsson, D., & Malm, T. (2008). Fouling assemblages on offshore wind power plants and adjacent substrata. Estuarine, Coastal and Shelf Science, 79(3), 459-466.	Windpark	Reefs		39.216
Wilhelmsson, D., & Malm, T. (2008). Fouling assemblages on offshore wind power plants and adjacent substrata. Estuarine, Coastal and Shelf Science, 79(3), 459-466.	Windpark	Reefs		0.965
Wilhelmsson, D., & Malm, T. (2008). Fouling assemblages on offshore wind power plants and adjacent substrata. Estuarine, Coastal and Shelf Science, 79(3), 459-466.	Windpark	Reefs		1.298
Wilhelmsson, D., & Malm, T. (2008). Fouling assemblages on offshore wind power plants and adjacent substrata. Estuarine, Coastal and Shelf Science, 79(3), 459-466.	Windpark	Reefs		16.984
Wilhelmsson, D., & Malm, T. (2008). Fouling assemblages on offshore wind power plants and adjacent substrata. Estuarine, Coastal and Shelf Science, 79(3), 459-466.	Windpark	Reefs		10.819
Wilhelmsson, D., & Malm, T. (2008). Fouling assemblages on offshore wind power plants and adjacent substrata. Estuarine, Coastal and Shelf Science, 79(3), 459-466.	Windpark	Reefs		1.991
Wilhelmsson, D., & Malm, T. (2008). Fouling assemblages on offshore wind power plants and adjacent substrata. Estuarine, Coastal and Shelf Science, 79(3), 459-466.	Windpark	Reefs		4.802
Wilhelmsson, D., & Malm, T. (2008). Fouling assemblages on offshore wind power plants and adjacent substrata. Estuarine, Coastal and Shelf Science, 79(3), 459-466.	Windpark	Reefs		0.013
Wilhelmsson, D., & Malm, T. (2008). Fouling assemblages on offshore wind power plants and adjacent substrata. Estuarine, Coastal and Shelf Science, 79(3), 459-466.	Windpark	Reefs		1.389
Wilhelmsson, D., & Malm, T. (2008). Fouling assemblages on offshore wind power plants and adjacent substrata. Estuarine, Coastal and Shelf Science, 79(3), 459-466.	Windpark	Reefs		1.312

Wilhelmsson, D., & Malm, T. (2008). Fouling assemblages on offshore wind power plants and adjacent substrata. Estuarine, Coastal and Shelf Science, 79(3), 459-466.	Windpark	Reefs		1.105
Wilhelmsson, D., & Malm, T. (2008). Fouling assemblages on offshore wind power plants and adjacent substrata. Estuarine, Coastal and Shelf Science, 79(3), 459-466.	Windpark	Reefs		0.218
Wilhelmsson, D., & Malm, T. (2008). Fouling assemblages on offshore wind power plants and adjacent substrata. Estuarine, Coastal and Shelf Science, 79(3), 459-466.	Windpark	Reefs		1.245
Wilhelmsson, D., & Malm, T. (2008). Fouling assemblages on offshore wind power plants and adjacent substrata. Estuarine, Coastal and Shelf Science, 79(3), 459-466.	Windpark	Reefs		0.259
Wilhelmsson, D., & Malm, T. (2008). Fouling assemblages on offshore wind power plants and adjacent substrata. Estuarine, Coastal and Shelf Science, 79(3), 459-466.	Windpark	Reefs		0.729
Wilhelmsson, D., & Malm, T. (2008). Fouling assemblages on offshore wind power plants and adjacent substrata. Estuarine, Coastal and Shelf Science, 79(3), 459-466.	Windpark	Reefs		0.205
Wilhelmsson, D., & Malm, T. (2008). Fouling assemblages on offshore wind power plants and adjacent substrata. Estuarine, Coastal and Shelf Science, 79(3), 459-466.	Windpark	Reefs		0.096
Wilhelmsson, D., & Malm, T. (2008). Fouling assemblages on offshore wind power plants and adjacent substrata. Estuarine, Coastal and Shelf Science, 79(3), 459-466.	Windpark	Reefs		0.008
Wilhelmsson, D., & Malm, T. (2008). Fouling assemblages on offshore wind power plants and adjacent substrata. Estuarine, Coastal and Shelf Science, 79(3), 459-466.	Windpark	Reefs		0.184
Wilhelmsson, D., & Malm, T. (2008). Fouling assemblages on offshore wind power plants and adjacent substrata. Estuarine, Coastal and Shelf Science, 79(3), 459-466.	Windpark	Reefs		3.071
Wilhelmsson, D., & Malm, T. (2008). Fouling assemblages on offshore wind power plants and adjacent substrata. Estuarine, Coastal and Shelf Science, 79(3), 459-466.	Windpark	Reefs		0.023
Wilhelmsson, D., & Malm, T. (2008). Fouling assemblages on offshore wind power plants and adjacent substrata. Estuarine, Coastal and Shelf Science, 79(3), 459-466.	Windpark	Reefs		0.922
Wilhelmsson, D., & Malm, T. (2008). Fouling assemblages on offshore wind power plants and adjacent substrata. Estuarine, Coastal and Shelf Science, 79(3), 459-466.	Windpark	Reefs		0.139
Wilhelmsson, D., & Malm, T. (2008). Fouling assemblages on offshore wind power plants and adjacent substrata. Estuarine, Coastal and Shelf Science, 79(3), 459-466.	Windpark	Reefs		0.975
Wilhelmsson, D., & Malm, T. (2008). Fouling assemblages on offshore wind power plants and adjacent substrata. Estuarine, Coastal and Shelf Science, 79(3), 459-466.	Windpark	Reefs		0.082
Wilhelmsson, D., & Malm, T. (2008). Fouling assemblages on offshore wind power plants and adjacent substrata. Estuarine, Coastal and Shelf Science, 79(3), 459-466.	Windpark	Reefs		1.017
Wilhelmsson, D., & Malm, T. (2008). Fouling assemblages on offshore wind power plants and adjacent substrata. Estuarine, Coastal and Shelf Science, 79(3), 459-466.	Windpark	Reefs		0.049
Wilhelmsson, D., & Malm, T. (2008). Fouling assemblages on offshore wind power plants and adjacent substrata. Estuarine, Coastal and Shelf Science, 79(3), 459-466.	Windpark	Reefs		1.160
Wilhelmsson, D., & Malm, T. (2008). Fouling assemblages on offshore wind power plants and adjacent substrata. Estuarine, Coastal and Shelf Science, 79(3), 459-466.	Windpark	Reefs		0.620

Wilhelmsson, D., & Malm, T. (2008). Fouling assemblages on offshore wind power plants and adjacent substrata. Estuarine, Coastal and Shelf Science, 79(3), 459-466.	Windpark	Reefs		0.000
Wilhelmsson, D., & Malm, T. (2008). Fouling assemblages on offshore wind power plants and adjacent substrata. Estuarine, Coastal and Shelf Science, 79(3), 459-466.	Windpark	Reefs		0.006
Wilhelmsson, D., & Malm, T. (2008). Fouling assemblages on offshore wind power plants and adjacent substrata. Estuarine, Coastal and Shelf Science, 79(3), 459-466.	Windpark	Reefs		0.001
Wilhelmsson, D., & Malm, T. (2008). Fouling assemblages on offshore wind power plants and adjacent substrata. Estuarine, Coastal and Shelf Science, 79(3), 459-466.	Windpark	Reefs		0.038
Wilhelmsson, D., & Malm, T. (2008). Fouling assemblages on offshore wind power plants and adjacent substrata. Estuarine, Coastal and Shelf Science, 79(3), 459-466.	Windpark	Reefs		0.024
Wilhelmsson, D., & Malm, T. (2008). Fouling assemblages on offshore wind power plants and adjacent substrata. Estuarine, Coastal and Shelf Science, 79(3), 459-466.	Windpark	Reefs		1.294
Wilhelmsson, D., & Malm, T. (2008). Fouling assemblages on offshore wind power plants and adjacent substrata. Estuarine, Coastal and Shelf Science, 79(3), 459-466.	Windpark	Reefs		0.244
Andersson et al, Epibenthic colonization of concrete and steel pilings in a cold-temperate embayment: a Weld experiment, 2009	Windpark	Reefs		*0.119*
Andersson et al, Epibenthic colonization of concrete and steel pilings in a cold-temperate embayment: a Weld experiment, 2009	Windpark	Reefs		0.058
Andersson et al, Epibenthic colonization of concrete and steel pilings in a cold-temperate embayment: a Weld experiment, 2009	Windpark	Reefs		0.241
Andersson et al, Epibenthic colonization of concrete and steel pilings in a cold-temperate embayment: a Weld experiment, 2009	Windpark	Reefs		0.115
Andersson et al, Epibenthic colonization of concrete and steel pilings in a cold-temperate embayment: a Weld experiment, 2009	Windpark	Reefs		*0.136*
Andersson et al, Epibenthic colonization of concrete and steel pilings in a cold-temperate embayment: a Weld experiment, 2009	Windpark	Reefs		1.000
Andersson et al, Epibenthic colonization of concrete and steel pilings in a cold-temperate embayment: a Weld experiment, 2009	Windpark	Reefs		0.278
Andersson et al, Epibenthic colonization of concrete and steel pilings in a cold-temperate embayment: a Weld experiment, 2009	Windpark	Reefs		*0.042*
Andersson et al, Epibenthic colonization of concrete and steel pilings in a cold-temperate embayment: a Weld experiment, 2009	Windpark	Reefs		1.000
Andersson et al, Epibenthic colonization of concrete and steel pilings in a cold-temperate embayment: a Weld experiment, 2009	Windpark	Reefs		0.084
Andersson et al, Epibenthic colonization of concrete and steel pilings in a cold-temperate embayment: a Weld experiment, 2009	Windpark	Reefs		*0.030*
Andersson et al, Epibenthic colonization of concrete and steel pilings in a cold-temperate embayment: a Weld experiment, 2009	Windpark	Reefs		1.000

Andersson et al, Epibenthic colonization of concrete and steel pilings in a cold-temperate embayment: a Weld experiment, 2009	Windpark	Reefs		0.060
Andersson et al, Epibenthic colonization of concrete and steel pilings in a cold-temperate embayment: a Weld experiment, 2009	Windpark	Reefs		*0.048*
Andersson et al, Epibenthic colonization of concrete and steel pilings in a cold-temperate embayment: a Weld experiment, 2009	Windpark	Reefs		0.020
Andersson et al, Epibenthic colonization of concrete and steel pilings in a cold-temperate embayment: a Weld experiment, 2009	Windpark	Reefs		0.096
Andersson et al, Epibenthic colonization of concrete and steel pilings in a cold-temperate embayment: a Weld experiment, 2009	Windpark	Reefs		*0.020*
Andersson et al, Epibenthic colonization of concrete and steel pilings in a cold-temperate embayment: a Weld experiment, 2009	Windpark	Reefs		0.205
Andersson et al, Epibenthic colonization of concrete and steel pilings in a cold-temperate embayment: a Weld experiment, 2009	Windpark	Reefs		0.193
Andersson et al, Epibenthic colonization of concrete and steel pilings in a cold-temperate embayment: a Weld experiment, 2009	Windpark	Reefs		0.429
Andersson et al, Epibenthic colonization of concrete and steel pilings in a cold-temperate embayment: a Weld experiment, 2009	Windpark	Reefs		*0.096*
Kautsky, H. 1992. The Impact of Pulp-Mill Effluents on Phytobenthic Communities in the Baltic Sea. Ambio, 21 (4): 308-313.	Nutrient input	Reefs		0.037
Kautsky, H. 1992. The Impact of Pulp-Mill Effluents on Phytobenthic Communities in the Baltic Sea. Ambio, 21 (4): 308-313.	Nutrient input	Reefs		0.028
Kautsky, H. 1992. The Impact of Pulp-Mill Effluents on Phytobenthic Communities in the Baltic Sea. Ambio, 21 (4): 308-313.	Nutrient input	Reefs		0.100
Kraufelin, P.; Moy, F.E.; Christie, H.; Bokn, T.L. 2006. Nutrient Addition to Experimental Rocky Shore Communities Revisited: Delayed Responses, Rapid Recovery. Ecosystem, 9: 1076-1093.	Nutrient input	Reefs		0.209
Kraufelin, P.; Moy, F.E.; Christie, H.; Bokn, T.L. 2006. Nutrient Addition to Experimental Rocky Shore Communities Revisited: Delayed Responses, Rapid Recovery. Ecosystem, 9: 1076-1093.	Nutrient input	Reefs		0.519
Kraufelin, P.; Moy, F.E.; Christie, H.; Bokn, T.L. 2006. Nutrient Addition to Experimental Rocky Shore Communities Revisited: Delayed Responses, Rapid Recovery. Ecosystem, 9: 1076-1093.	Nutrient input	Reefs		1.182
Kraufelin, P.; Moy, F.E.; Christie, H.; Bokn, T.L. 2006. Nutrient Addition to Experimental Rocky Shore Communities Revisited: Delayed Responses, Rapid Recovery. Ecosystem, 9: 1076-1093.	Nutrient input	Reefs		1.735
Kraufelin, P.; Moy, F.E.; Christie, H.; Bokn, T.L. 2006. Nutrient Addition to Experimental Rocky Shore Communities Revisited: Delayed Responses, Rapid Recovery. Ecosystem, 9: 1076-1093.	Nutrient input	Reefs		0.167
Kraufelin, P.; Moy, F.E.; Christie, H.; Bokn, T.L. 2006. Nutrient Addition to Experimental Rocky Shore Communities Revisited: Delayed Responses, Rapid Recovery. Ecosystem, 9: 1076-1093.	Nutrient input	Reefs		0.237
Kraufelin, P.; Moy, F.E.; Christie, H.; Bokn, T.L. 2006. Nutrient Addition to Experimental Rocky Shore Communities Revisited: Delayed Responses, Rapid Recovery. Ecosystem, 9: 1076-1093.	Nutrient input	Reefs		0.346

Kraufelin, P.; Moy, F.E.; Christie, H.; Bokn, T.L. 2006. Nutrient Addition to Experimental Rocky Shore Communities Revisited: Delayed Responses, Rapid Recovery. Ecosystem, 9: 1076-1093.	Nutrient input	Reefs		0.688
Kraufelin, P.; Moy, F.E.; Christie, H.; Bokn, T.L. 2006. Nutrient Addition to Experimental Rocky Shore Communities Revisited: Delayed Responses, Rapid Recovery. Ecosystem, 9: 1076-1093.	Nutrient input	Reefs		1.127
Kraufelin, P.; Moy, F.E.; Christie, H.; Bokn, T.L. 2006. Nutrient Addition to Experimental Rocky Shore Communities Revisited: Delayed Responses, Rapid Recovery. Ecosystem, 9: 1076-1093.	Nutrient input	Reefs		2.027
Kraufelin, P.; Moy, F.E.; Christie, H.; Bokn, T.L. 2006. Nutrient Addition to Experimental Rocky Shore Communities Revisited: Delayed Responses, Rapid Recovery. Ecosystem, 9: 1076-1093.	Nutrient input	Reefs		0.217
Kraufelin, P.; Moy, F.E.; Christie, H.; Bokn, T.L. 2006. Nutrient Addition to Experimental Rocky Shore Communities Revisited: Delayed Responses, Rapid Recovery. Ecosystem, 9: 1076-1093.	Nutrient input	Reefs		0.296
Kraufelin, P.; Moy, F.E.; Christie, H.; Bokn, T.L. 2006. Nutrient Addition to Experimental Rocky Shore Communities Revisited: Delayed Responses, Rapid Recovery. Ecosystem, 9: 1076-1093.	Nutrient input	Reefs		0.360
Kraufelin, P.; Moy, F.E.; Christie, H.; Bokn, T.L. 2006. Nutrient Addition to Experimental Rocky Shore Communities Revisited: Delayed Responses, Rapid Recovery. Ecosystem, 9: 1076-1093.	Nutrient input	Reefs		0.662
Kraufelin, P.; Moy, F.E.; Christie, H.; Bokn, T.L. 2006. Nutrient Addition to Experimental Rocky Shore Communities Revisited: Delayed Responses, Rapid Recovery. Ecosystem, 9: 1076-1093.	Nutrient input	Reefs		1.188
Kraufelin, P.; Moy, F.E.; Christie, H.; Bokn, T.L. 2006. Nutrient Addition to Experimental Rocky Shore Communities Revisited: Delayed Responses, Rapid Recovery. Ecosystem, 9: 1076-1093.	Nutrient input	Reefs		1.297
Kraufelin, P.; Moy, F.E.; Christie, H.; Bokn, T.L. 2006. Nutrient Addition to Experimental Rocky Shore Communities Revisited: Delayed Responses, Rapid Recovery. Ecosystem, 9: 1076-1093.	Nutrient input	Reefs		0.667
Kraufelin, P.; Moy, F.E.; Christie, H.; Bokn, T.L. 2006. Nutrient Addition to Experimental Rocky Shore Communities Revisited: Delayed Responses, Rapid Recovery. Ecosystem, 9: 1076-1093.	Nutrient input	Reefs		0.044
Kraufelin, P.; Moy, F.E.; Christie, H.; Bokn, T.L. 2006. Nutrient Addition to Experimental Rocky Shore Communities Revisited: Delayed Responses, Rapid Recovery. Ecosystem, 9: 1076-1093.	Nutrient input	Reefs		0.290
Kraufelin, P.; Moy, F.E.; Christie, H.; Bokn, T.L. 2006. Nutrient Addition to Experimental Rocky Shore Communities Revisited: Delayed Responses, Rapid Recovery. Ecosystem, 9: 1076-1093.	Nutrient input	Reefs		0.818
Kraufelin, P.; Moy, F.E.; Christie, H.; Bokn, T.L. 2006. Nutrient Addition to Experimental Rocky Shore Communities Revisited: Delayed Responses, Rapid Recovery. Ecosystem, 9: 1076-1093.	Nutrient input	Reefs		1.200
Kraufelin, P.; Moy, F.E.; Christie, H.; Bokn, T.L. 2006. Nutrient Addition to Experimental Rocky Shore Communities Revisited: Delayed Responses, Rapid Recovery. Ecosystem, 9: 1076-1093.	Nutrient input	Reefs		0.703
Kraufelin, P.; Moy, F.E.; Christie, H.; Bokn, T.L. 2006. Nutrient Addition to Experimental Rocky Shore Communities Revisited: Delayed Responses, Rapid Recovery. Ecosystem, 9: 1076-1093.	Nutrient input	Reefs		0.960
Kraufelin, P.; Moy, F.E.; Christie, H.; Bokn, T.L. 2006. Nutrient Addition to Experimental Rocky Shore Communities Revisited: Delayed Responses, Rapid Recovery. Ecosystem, 9: 1076-1093.	Nutrient input	Reefs		0.568
Kraufelin, P.; Moy, F.E.; Christie, H.; Bokn, T.L. 2006. Nutrient Addition to Experimental Rocky Shore Communities Revisited: Delayed Responses, Rapid Recovery. Ecosystem, 9: 1076-1093.	Nutrient input	Reefs		0.273
Kraufelin, P.; Moy, F.E.; Christie, H.; Bokn, T.L. 2006. Nutrient Addition to Experimental Rocky Shore Communities Revisited: Delayed Responses, Rapid Recovery. Ecosystem, 9: 1076-1093.	Nutrient input	Reefs		0.844

Kraufelin, P.; Moy, F.E.; Christie, H.; Bokn, T.L. 2006. Nutrient Addition to Experimental Rocky Shore Communities Revisited: Delayed Responses, Rapid Recovery. Ecosystem, 9: 1076-1093.	Nutrient input	Reefs		1.152
Kraufelin, P.; Moy, F.E.; Christie, H.; Bokn, T.L. 2006. Nutrient Addition to Experimental Rocky Shore Communities Revisited: Delayed Responses, Rapid Recovery. Ecosystem, 9: 1076-1093.	Nutrient input	Reefs		0.541
Kraufelin, P.; Moy, F.E.; Christie, H.; Bokn, T.L. 2006. Nutrient Addition to Experimental Rocky Shore Communities Revisited: Delayed Responses, Rapid Recovery. Ecosystem, 9: 1076-1093.	Nutrient input	Reefs		0.284
Kraufelin, P.; Moy, F.E.; Christie, H.; Bokn, T.L. 2006. Nutrient Addition to Experimental Rocky Shore Communities Revisited: Delayed Responses, Rapid Recovery. Ecosystem, 9: 1076-1093.	Nutrient input	Reefs		0.296
Kraufelin, P.; Moy, F.E.; Christie, H.; Bokn, T.L. 2006. Nutrient Addition to Experimental Rocky Shore Communities Revisited: Delayed Responses, Rapid Recovery. Ecosystem, 9: 1076-1093.	Nutrient input	Reefs		0.500
Kraufelin, P.; Moy, F.E.; Christie, H.; Bokn, T.L. 2006. Nutrient Addition to Experimental Rocky Shore Communities Revisited: Delayed Responses, Rapid Recovery. Ecosystem, 9: 1076-1093.	Nutrient input	Reefs		0.597
Kraufelin, P.; Moy, F.E.; Christie, H.; Bokn, T.L. 2006. Nutrient Addition to Experimental Rocky Shore Communities Revisited: Delayed Responses, Rapid Recovery. Ecosystem, 9: 1076-1093.	Nutrient input	Reefs		1.145
Kraufelin, P.; Moy, F.E.; Christie, H.; Bokn, T.L. 2006. Nutrient Addition to Experimental Rocky Shore Communities Revisited: Delayed Responses, Rapid Recovery. Ecosystem, 9: 1076-1093.	Nutrient input	Reefs		0.622
Kraufelin, P.; Moy, F.E.; Christie, H.; Bokn, T.L. 2006. Nutrient Addition to Experimental Rocky Shore Communities Revisited: Delayed Responses, Rapid Recovery. Ecosystem, 9: 1076-1093.	Nutrient input	Reefs		1.000
Kraufelin, P.; Moy, F.E.; Christie, H.; Bokn, T.L. 2006. Nutrient Addition to Experimental Rocky Shore Communities Revisited: Delayed Responses, Rapid Recovery. Ecosystem, 9: 1076-1093.	Nutrient input	Reefs		0.355
Kraufelin, P.; Moy, F.E.; Christie, H.; Bokn, T.L. 2006. Nutrient Addition to Experimental Rocky Shore Communities Revisited: Delayed Responses, Rapid Recovery. Ecosystem, 9: 1076-1093.	Nutrient input	Reefs		0.591
Kraufelin, P.; Moy, F.E.; Christie, H.; Bokn, T.L. 2006. Nutrient Addition to Experimental Rocky Shore Communities Revisited: Delayed Responses, Rapid Recovery. Ecosystem, 9: 1076-1093.	Nutrient input	Reefs		0.637
Kraufelin, P.; Moy, F.E.; Christie, H.; Bokn, T.L. 2006. Nutrient Addition to Experimental Rocky Shore Communities Revisited: Delayed Responses, Rapid Recovery. Ecosystem, 9: 1076-1093.	Nutrient input	Reefs		0.588
Kraufelin, P.; Moy, F.E.; Christie, H.; Bokn, T.L. 2006. Nutrient Addition to Experimental Rocky Shore Communities Revisited: Delayed Responses, Rapid Recovery. Ecosystem, 9: 1076-1093.	Nutrient input	Reefs		0.561
Kraufelin, P.; Moy, F.E.; Christie, H.; Bokn, T.L. 2006. Nutrient Addition to Experimental Rocky Shore Communities Revisited: Delayed Responses, Rapid Recovery. Ecosystem, 9: 1076-1093.	Nutrient input	Reefs		1.187
Bokn, T.L.; Duarte, C.M.; Pedersen, M.F.; Marba, N.; Moy, F.E.; Barron, C. et al. 2003. The Response of Experimental Rocky Shore Communities to Nutrient Additions. Ecosystems, 6: 577-594.	Nutrient input	Reefs		0.905
Bokn, T.L.; Duarte, C.M.; Pedersen, M.F.; Marba, N.; Moy, F.E.; Barron, C. et al. 2003. The Response of Experimental Rocky Shore Communities to Nutrient Additions. Ecosystems, 6: 577-594.	Nutrient input	Reefs		0.887
Bokn, T.L.; Duarte, C.M.; Pedersen, M.F.; Marba, N.; Moy, F.E.; Barron, C. et al. 2003. The Response of Experimental Rocky Shore Communities to Nutrient Additions. Ecosystems, 6: 577-594.	Nutrient input	Reefs		1.062
Bokn, T.L.; Duarte, C.M.; Pedersen, M.F.; Marba, N.; Moy, F.E.; Barron, C. et al. 2003. The Response of Experimental Rocky Shore Communities to Nutrient Additions. Ecosystems, 6: 577-594.	Nutrient input	Reefs		0.583

Bokn, T.L.; Duarte, C.M.; Pedersen, M.F.; Marba, N.; Moy, F.E.; Barron, C. et al. 2003. The Response of Experimental Rocky Shore Communities to Nutrient Additions. Ecosystems, 6: 577-594.	Nutrient input	Reefs		1.405
Bokn, T.L.; Duarte, C.M.; Pedersen, M.F.; Marba, N.; Moy, F.E.; Barron, C. et al. 2003. The Response of Experimental Rocky Shore Communities to Nutrient Additions. Ecosystems, 6: 577-594.	Nutrient input	Reefs		1.174
Bokn, T.L.; Duarte, C.M.; Pedersen, M.F.; Marba, N.; Moy, F.E.; Barron, C. et al. 2003. The Response of Experimental Rocky Shore Communities to Nutrient Additions. Ecosystems, 6: 577-594.	Nutrient input	Reefs		1.010
Bokn, T.L.; Duarte, C.M.; Pedersen, M.F.; Marba, N.; Moy, F.E.; Barron, C. et al. 2003. The Response of Experimental Rocky Shore Communities to Nutrient Additions. Ecosystems, 6: 577-594.	Nutrient input	Reefs		0.391
Bokn, T.L.; Duarte, C.M.; Pedersen, M.F.; Marba, N.; Moy, F.E.; Barron, C. et al. 2003. The Response of Experimental Rocky Shore Communities to Nutrient Additions. Ecosystems, 6: 577-594.	Nutrient input	Reefs		1.030
Bokn, T.L.; Duarte, C.M.; Pedersen, M.F.; Marba, N.; Moy, F.E.; Barron, C. et al. 2003. The Response of Experimental Rocky Shore Communities to Nutrient Additions. Ecosystems, 6: 577-594.	Nutrient input	Reefs		1.010
Bokn, T.L.; Duarte, C.M.; Pedersen, M.F.; Marba, N.; Moy, F.E.; Barron, C. et al. 2003. The Response of Experimental Rocky Shore Communities to Nutrient Additions. Ecosystems, 6: 577-594.	Nutrient input	Reefs		1.062
Bokn, T.L.; Duarte, C.M.; Pedersen, M.F.; Marba, N.; Moy, F.E.; Barron, C. et al. 2003. The Response of Experimental Rocky Shore Communities to Nutrient Additions. Ecosystems, 6: 577-594.	Nutrient input	Reefs		0.340
Bokn, T.L.; Duarte, C.M.; Pedersen, M.F.; Marba, N.; Moy, F.E.; Barron, C. et al. 2003. The Response of Experimental Rocky Shore Communities to Nutrient Additions. Ecosystems, 6: 577-594.	Nutrient input	Reefs		0.980
Bokn, T.L.; Duarte, C.M.; Pedersen, M.F.; Marba, N.; Moy, F.E.; Barron, C. et al. 2003. The Response of Experimental Rocky Shore Communities to Nutrient Additions. Ecosystems, 6: 577-594.	Nutrient input	Reefs		0.980
Bokn, T.L.; Duarte, C.M.; Pedersen, M.F.; Marba, N.; Moy, F.E.; Barron, C. et al. 2003. The Response of Experimental Rocky Shore Communities to Nutrient Additions. Ecosystems, 6: 577-594.	Nutrient input	Reefs		0.684
Bokn, T.L.; Duarte, C.M.; Pedersen, M.F.; Marba, N.; Moy, F.E.; Barron, C. et al. 2003. The Response of Experimental Rocky Shore Communities to Nutrient Additions. Ecosystems, 6: 577-594.	Nutrient input	Reefs		0.353
Bokn, T.L.; Duarte, C.M.; Pedersen, M.F.; Marba, N.; Moy, F.E.; Barron, C. et al. 2003. The Response of Experimental Rocky Shore Communities to Nutrient Additions. Ecosystems, 6: 577-594.	Nutrient input	Reefs		1.197
Bokn, T.L.; Duarte, C.M.; Pedersen, M.F.; Marba, N.; Moy, F.E.; Barron, C. et al. 2003. The Response of Experimental Rocky Shore Communities to Nutrient Additions. Ecosystems, 6: 577-594.	Nutrient input	Reefs		1.209
Bokn, T.L.; Duarte, C.M.; Pedersen, M.F.; Marba, N.; Moy, F.E.; Barron, C. et al. 2003. The Response of Experimental Rocky Shore Communities to Nutrient Additions. Ecosystems, 6: 577-594.	Nutrient input	Reefs		1.010
Bokn, T.L.; Duarte, C.M.; Pedersen, M.F.; Marba, N.; Moy, F.E.; Barron, C. et al. 2003. The Response of Experimental Rocky Shore Communities to Nutrient Additions. Ecosystems, 6: 577-594.	Nutrient input	Reefs		0.803
Bokn, T.L.; Duarte, C.M.; Pedersen, M.F.; Marba, N.; Moy, F.E.; Barron, C. et al. 2003. The Response of Experimental Rocky Shore Communities to Nutrient Additions. Ecosystems, 6: 577-594.	Nutrient input	Reefs		1.010
Bokn, T.L.; Duarte, C.M.; Pedersen, M.F.; Marba, N.; Moy, F.E.; Barron, C. et al. 2003. The Response of Experimental Rocky Shore Communities to Nutrient Additions. Ecosystems, 6: 577-594.	Nutrient input	Reefs		0.942
Bokn, T.L.; Duarte, C.M.; Pedersen, M.F.; Marba, N.; Moy, F.E.; Barron, C. et al. 2003. The Response of Experimental Rocky Shore Communities to Nutrient Additions. Ecosystems, 6: 577-594.	Nutrient input	Reefs		1.020

Bokn, T.L.; Duarte, C.M.; Pedersen, M.F.; Marba, N.; Moy, F.E.; Barron, C. et al. 2003. The Response of Experimental Rocky Shore Communities to Nutrient Additions. Ecosystems, 6: 577-594.	Nutrient input	Reefs		0.492
Vogt, H. And Schramm, W. 1991. Conspicuous decline of Fucus in Kiel Bay (Western Baltic): what are the causes? MARINE ECOLOGY PROGRESS SERIES, 69: 189-194.	Nutrient input	Reefs		0.091
Kraufvelin, P. 2007. Responses to nutrient enrichment, wave action and disturbance in rocky shore communities. Aquatic Botany 87: 262–282	Nutrient input	Reefs		0.545
Kraufvelin, P. 2007. Responses to nutrient enrichment, wave action and disturbance in rocky shore communities. Aquatic Botany 87: 262–283	Nutrient input	Reefs		0.046
Kraufvelin, P. 2007. Responses to nutrient enrichment, wave action and disturbance in rocky shore communities. Aquatic Botany 87: 262–284	Nutrient input	Reefs		0.253
Kraufvelin, P. 2007. Responses to nutrient enrichment, wave action and disturbance in rocky shore communities. Aquatic Botany 87: 262–285	Nutrient input	Reefs		1.167
Kraufvelin, P. 2007. Responses to nutrient enrichment, wave action and disturbance in rocky shore communities. Aquatic Botany 87: 262–286	Nutrient input	Reefs		0.048
Kraufvelin, P. 2007. Responses to nutrient enrichment, wave action and disturbance in rocky shore communities. Aquatic Botany 87: 262–287	Nutrient input	Reefs		1.216
Kraufvelin, P. 2007. Responses to nutrient enrichment, wave action and disturbance in rocky shore communities. Aquatic Botany 87: 262–288	Nutrient input	Reefs		1.920
Kraufvelin, P. 2007. Responses to nutrient enrichment, wave action and disturbance in rocky shore communities. Aquatic Botany 87: 262–289	Nutrient input	Reefs		2.790
Kraufvelin, P. 2007. Responses to nutrient enrichment, wave action and disturbance in rocky shore communities. Aquatic Botany 87: 262–290	Nutrient input	Reefs		2.201
Kraufvelin, P. 2007. Responses to nutrient enrichment, wave action and disturbance in rocky shore communities. Aquatic Botany 87: 262–291	Nutrient input	Reefs		0.556
Kraufvelin, P. 2007. Responses to nutrient enrichment, wave action and disturbance in rocky shore communities. Aquatic Botany 87: 262–292	Nutrient input	Reefs		1.064
Kraufvelin, P. 2007. Responses to nutrient enrichment, wave action and disturbance in rocky shore communities. Aquatic Botany 87: 262–300	Nutrient input	Reefs		0.119
Kraufvelin, P. 2007. Responses to nutrient enrichment, wave action and disturbance in rocky shore communities. Aquatic Botany 87: 262–301	Nutrient input	Reefs		2.856
Kraufvelin, P. 2007. Responses to nutrient enrichment, wave action and disturbance in rocky shore communities. Aquatic Botany 87: 262–302	Nutrient input	Reefs		3.411
Kraufvelin, P. 2007. Responses to nutrient enrichment, wave action and disturbance in rocky shore communities. Aquatic Botany 87: 262–302	Nutrient input	Reefs		5.300
Kraufvelin, P. 2007. Responses to nutrient enrichment, wave action and disturbance in rocky shore communities. Aquatic Botany 87: 262–303	Nutrient input	Reefs		2.892
Kraufvelin, P. 2007. Responses to nutrient enrichment, wave action and disturbance in rocky shore communities. Aquatic Botany 87: 262–304	Nutrient input	Reefs		0.662
Jormalainen, V., Gagnon, K., Sjöroos, J., & Rothäusler, E. (2016). The invasive mud crab enforces a major shift in a rocky littoral invertebrate community of the Baltic Sea. Biological invasions, 18(5), 1409-1419.	Mud crab	Reefs	*Fucus*	0.268

Wilhelmsson, D., & Malm, T. (2008). Fouling assemblages on offshore wind power plants and adjacent substrata. Estuarine, Coastal and Shelf Science, 79(3), 459-466.	Wind park	Reefs	*Fucus*	0.620
Kraufelin, P.; Moy, F.E.; Christie, H.; Bokn, T.L. 2006. Nutrient Addition to Experimental Rocky Shore Communities Revisited: Delayed Responses, Rapid Recovery. Ecosystem, 9: 1076-1093.	Nutrient input	Reefs	*Fucus*	0.519
Kraufelin, P.; Moy, F.E.; Christie, H.; Bokn, T.L. 2006. Nutrient Addition to Experimental Rocky Shore Communities Revisited: Delayed Responses, Rapid Recovery. Ecosystem, 9: 1076-1093.	Nutrient input	Reefs	*Fucus*	0.688
Kraufelin, P.; Moy, F.E.; Christie, H.; Bokn, T.L. 2006. Nutrient Addition to Experimental Rocky Shore Communities Revisited: Delayed Responses, Rapid Recovery. Ecosystem, 9: 1076-1093.	Nutrient input	Reefs	*Fucus*	0.662
Kraufelin, P.; Moy, F.E.; Christie, H.; Bokn, T.L. 2006. Nutrient Addition to Experimental Rocky Shore Communities Revisited: Delayed Responses, Rapid Recovery. Ecosystem, 9: 1076-1093.	Nutrient input	Reefs	*Fucus*	0.818
Kraufelin, P.; Moy, F.E.; Christie, H.; Bokn, T.L. 2006. Nutrient Addition to Experimental Rocky Shore Communities Revisited: Delayed Responses, Rapid Recovery. Ecosystem, 9: 1076-1093.	Nutrient input	Reefs	*Fucus*	0.844
Kraufelin, P.; Moy, F.E.; Christie, H.; Bokn, T.L. 2006. Nutrient Addition to Experimental Rocky Shore Communities Revisited: Delayed Responses, Rapid Recovery. Ecosystem, 9: 1076-1093.	Nutrient input	Reefs	*Fucus*	0.597
Kraufelin, P.; Moy, F.E.; Christie, H.; Bokn, T.L. 2006. Nutrient Addition to Experimental Rocky Shore Communities Revisited: Delayed Responses, Rapid Recovery. Ecosystem, 9: 1076-1093.	Nutrient input	Reefs	*Fucus*	0.588
Kraufelin, P.; Moy, F.E.; Christie, H.; Bokn, T.L. 2006. Nutrient Addition to Experimental Rocky Shore Communities Revisited: Delayed Responses, Rapid Recovery. Ecosystem, 9: 1076-1093.	Nutrient input	Reefs	*Fucus*	0.561
Kraufvelin, P. 2007. Responses to nutrient enrichment, wave action and disturbance in rocky shore communities. Aquatic Botany 87: 262–282	Nutrient input	Reefs	*Fucus*	0.545
Kraufvelin, P. 2007. Responses to nutrient enrichment, wave action and disturbance in rocky shore communities. Aquatic Botany 87: 262–285	Nutrient input	Reefs	*Fucus*	1.167
Wilhelmsson, D., & Malm, T. (2008). Fouling assemblages on offshore wind power plants and adjacent substrata. Estuarine, Coastal and Shelf Science, 79(3), 459-466.	Wind park	Reefs	*Furcellaria*	0.000
Bokn, T.L.; Duarte, C.M.; Pedersen, M.F.; Marba, N.; Moy, F.E.; Barron, C. et al. 2003. The Response of Experimental Rocky Shore Communities to Nutrient Additions. Ecosystems, 6: 577-594.	Nutrient input	Reefs	*Furcellaria*	1.062
Bokn, T.L.; Duarte, C.M.; Pedersen, M.F.; Marba, N.; Moy, F.E.; Barron, C. et al. 2003. The Response of Experimental Rocky Shore Communities to Nutrient Additions. Ecosystems, 6: 577-594.	Nutrient input	Reefs	*Furcellaria*	1.010
Bokn, T.L.; Duarte, C.M.; Pedersen, M.F.; Marba, N.; Moy, F.E.; Barron, C. et al. 2003. The Response of Experimental Rocky Shore Communities to Nutrient Additions. Ecosystems, 6: 577-594.	Nutrient input	Reefs	*Furcellaria*	1.062
Bokn, T.L.; Duarte, C.M.; Pedersen, M.F.; Marba, N.; Moy, F.E.; Barron, C. et al. 2003. The Response of Experimental Rocky Shore Communities to Nutrient Additions. Ecosystems, 6: 577-594.	Nutrient input	Reefs	*Furcellaria*	0.684
Bokn, T.L.; Duarte, C.M.; Pedersen, M.F.; Marba, N.; Moy, F.E.; Barron, C. et al. 2003. The Response of Experimental Rocky Shore Communities to Nutrient Additions. Ecosystems, 6: 577-594.	Nutrient input	Reefs	*Furcellaria*	1.010
Bokn, T.L.; Duarte, C.M.; Pedersen, M.F.; Marba, N.; Moy, F.E.; Barron, C. et al. 2003. The Response of Experimental Rocky Shore Communities to Nutrient Additions. Ecosystems, 6: 577-594.	Nutrient input	Reefs	*Furcellaria*	1.020
Nurkse, K., Kotta, J., Orav-Kotta, H., Ojaveer, H. (2016) A successful non-native predator, round goby, in the Baltic Sea: generalist feeding strategy, diverse diet and high prey consumption. Hydrobiologia 777, 271–281.	Round goby	Reefs	*Mytilus*	0.341
Nurkse, K., Kotta, J., Orav-Kotta, H., Ojaveer, H. (2016) A successful non-native predator, round goby, in the Baltic Sea: generalist feeding strategy, diverse diet and high prey consumption. Hydrobiologia 777, 271–281.	Round goby	Reefs	*Mytilus*	0.223
European Invasive Species impact database, No 68; Skabeikis, A., Morkūnė, R., Bacevičius, E., Lesutienė, J., Morkūnas, J., Poškienė, A., & Siaulys, A. (2019). Effect of round goby (Neogobius melanostomus) invasion on blue mussel (Mytilus edulis trossulus) population and winter diet of the long-tailed duck (Clangula hyemalis). Biological Invasions, 21(3), 911-923.	Round goby	Reefs	*Mytilus*	0.000
European Invasive Species impact database, No 69; Skabeikis, A., Morkūnė, R., Bacevičius, E., Lesutienė, J., Morkūnas, J., Poškienė, A., & Siaulys, A. (2019). Effect of round goby (Neogobius melanostomus) invasion on blue mussel (Mytilus edulis trossulus) population and winter diet of the long-tailed duck (Clangula hyemalis). Biological Invasions, 21(3), 911-923.	Round goby	Reefs	*Mytilus*	0.013
European Invasive Species impact database, No 70; Skabeikis, A., Morkūnė, R., Bacevičius, E., Lesutienė, J., Morkūnas, J., Poškienė, A., & Siaulys, A. (2019). Effect of round goby (Neogobius melanostomus) invasion on blue mussel (Mytilus edulis trossulus) population and winter diet of the long-tailed duck (Clangula hyemalis). Biological Invasions, 21(3), 911-923.	Round goby	Reefs	*Mytilus*	0.286
European Invasive Species impact database, No 79; Wiegleb, J., Kotterba, P., Hammer, C., & Oesterwind, D. (2018). Predation of the round goby (Neogobius melanostomus Pallas, 1814) on Atlantic herring eggs in the Western Baltic Sea. Marine Biology Research, 14(9-10), 989-1003.	Round goby	Reefs	*Mytilus*	1.452
European Invasive Species impact database, No 80; Wiegleb, J., Kotterba, P., Hammer, C., & Oesterwind, D. (2018). Predation of the round goby (Neogobius melanostomus Pallas, 1814) on Atlantic herring eggs in the Western Baltic Sea. Marine Biology Research, 14(9-10), 989-1003.	Round goby	Reefs	*Mytilus*	5.000
European Invasive Species impact database, No 86; Wiegleb, J., Kotterba, P., Hammer, C., & Oesterwind, D. (2018). Predation of the round goby (Neogobius melanostomus Pallas, 1814) on Atlantic herring eggs in the Western Baltic Sea. Marine Biology Research, 14(9-10), 989-1003.	Round goby	Reefs	*Mytilus*	2.530

European Invasive Species impact database, No 87; Wiegleb, J., Kotterba, P., Hammer, C., & Oesterwind, D. (2018). Predation of the round goby (Neogobius melanostomus Pallas, 1814) on Atlantic herring eggs in the Western Baltic Sea. Marine Biology Research, 14(9-10), 989-1003.	Round goby	Reefs	*Mytilus*	4.000
Nurkse, K., Kotta, J., Orav-Kotta, H., Ojaveer, H. (2016) A successful non-native predator, round goby, in the Baltic Sea: generalist feeding strategy, diverse diet and high prey consumption. Hydrobiologia 777, 271–281.	Round goby	Reefs	*Mytilus*	0.341
Nurkse, K., Kotta, J., Orav-Kotta, H., Ojaveer, H. (2016) A successful non-native predator, round goby, in the Baltic Sea: generalist feeding strategy, diverse diet and high prey consumption. Hydrobiologia 777, 271–281.	Round goby	Reefs	*Mytilus*	0.223
European Invasive Species impact database, No 68; Skabeikis, A., Morkūnė, R., Bacevičius, E., Lesutienė, J., Morkūnas, J., Poškienė, A., & Siaulys, A. (2019). Effect of round goby (Neogobius melanostomus) invasion on blue mussel (Mytilus edulis trossulus) population and winter diet of the long-tailed duck (Clangula hyemalis). Biological Invasions, 21(3), 911-923.	Round goby	Reefs	*Mytilus*	0.000
European Invasive Species impact database, No 68; Skabeikis, A., Morkūnė, R., Bacevičius, E., Lesutienė, J., Morkūnas, J., Poškienė, A., & Siaulys, A. (2019). Effect of round goby (Neogobius melanostomus) invasion on blue mussel (Mytilus edulis trossulus) population and winter diet of the long-tailed duck (Clangula hyemalis). Biological Invasions, 21(3), 911-923.	Round goby	Reefs	*Mytilus*	0.000
European Invasive Species impact database, No 69; Skabeikis, A., Morkūnė, R., Bacevičius, E., Lesutienė, J., Morkūnas, J., Poškienė, A., & Siaulys, A. (2019). Effect of round goby (Neogobius melanostomus) invasion on blue mussel (Mytilus edulis trossulus) population and winter diet of the long-tailed duck (Clangula hyemalis). Biological Invasions, 21(3), 911-923.	Round goby	Reefs	*Mytilus*	0.013
European Invasive Species impact database, No 69; Skabeikis, A., Morkūnė, R., Bacevičius, E., Lesutienė, J., Morkūnas, J., Poškienė, A., & Siaulys, A. (2019). Effect of round goby (Neogobius melanostomus) invasion on blue mussel (Mytilus edulis trossulus) population and winter diet of the long-tailed duck (Clangula hyemalis). Biological Invasions, 21(3), 911-923.	Round goby	Reefs	*Mytilus*	0.013
European Invasive Species impact database, No 70; Skabeikis, A., Morkūnė, R., Bacevičius, E., Lesutienė, J., Morkūnas, J., Poškienė, A., & Siaulys, A. (2019). Effect of round goby (Neogobius melanostomus) invasion on blue mussel (Mytilus edulis trossulus) population and winter diet of the long-tailed duck (Clangula hyemalis). Biological Invasions, 21(3), 911-923.	Round goby	Reefs	*Mytilus*	0.286
European Invasive Species impact database, No 70; Skabeikis, A., Morkūnė, R., Bacevičius, E., Lesutienė, J., Morkūnas, J., Poškienė, A., & Siaulys, A. (2019). Effect of round goby (Neogobius melanostomus) invasion on blue mussel (Mytilus edulis trossulus) population and winter diet of the long-tailed duck (Clangula hyemalis). Biological Invasions, 21(3), 911-923.	Round goby	Reefs	*Mytilus*	0.286
European Invasive Species impact database, No 79; Wiegleb, J., Kotterba, P., Hammer, C., & Oesterwind, D. (2018). Predation of the round goby (Neogobius melanostomus Pallas, 1814) on Atlantic herring eggs in the Western Baltic Sea. Marine Biology Research, 14(9-10), 989-1003.	Round goby	Reefs	*Mytilus*	1.452
European Invasive Species impact database, No 86; Wiegleb, J., Kotterba, P., Hammer, C., & Oesterwind, D. (2018). Predation of the round goby (Neogobius melanostomus Pallas, 1814) on Atlantic herring eggs in the Western Baltic Sea. Marine Biology Research, 14(9-10), 989-1003.	Round goby	Reefs	*Mytilus*	2.530
Nurkse, K., Kotta, J., Rätsep, M., Kotta, I., Kreitsberg, R., 2018. Experimental evaluation of the effects of the novel predators, round goby and mud crab on benthic invertebrates in the Gulf of Riga, Baltic Sea. J. Mar. Biol. Assoc. U. K. 98, 25–31.	Mud crab	Reefs	*Mytilus*	0.980
Forsström, T., Fowler, A. E., Manninen, I., & Vesakoski, O. (2015). An introduced species meets the local fauna: predatory behavior of the crab Rhithropanopeus harrisii in the Northern Baltic Sea. Biological invasions, 17(9), 2729-2741.	Mud crab	Reefs	*Mytilus*	0.081
Jormalainen, V., Gagnon, K., Sjöroos, J., & Rothäusler, E. (2016). The invasive mud crab enforces a major shift in a rocky littoral invertebrate community of the Baltic Sea. Biological invasions, 18(5), 1409-1419.	Mud crab	Reefs	*Mytilus*	1.000
Nurkse, K., Kotta, J., Rätsep, M., Kotta, I., Kreitsberg, R., 2018. Experimental evaluation of the effects of the novel predators, round goby and mud crab on benthic invertebrates in the Gulf of Riga, Baltic Sea. J. Mar. Biol. Assoc. U. K. 98, 25–31.	Round goby and M	Reefs	*Mytilus*	0.881
Raoux et al. 2017, Benthic and fish aggregation inside an offshore wind farm, Ecological Indicators 72, 33-46	Wind park	Reefs	*Mytilus*	1.532
Nurkse, K., Kotta, J., Rätsep, M., Kotta, I., Kreitsberg, R., 2018. Experimental evaluation of the effects of the novel predators, round goby and mud crab on benthic invertebrates in the Gulf of Riga, Baltic Sea. J. Mar. Biol. Assoc. U. K. 98, 25–31.	Round goby	Sandbanks		0.954
Coates et al. 2016, Short-term effects of fishery exclusion in offshore wind farms, Fisheries Research, 179, 131-138	Round goby	Sandbanks		0.999
Coates et al. 2016, Short-term effects of fishery exclusion in offshore wind farms, Fisheries Research, 179, 131-138	Round goby	Sandbanks		0.876
Coates et al. 2016, Short-term effects of fishery exclusion in offshore wind farms, Fisheries Research, 179, 131-138	Round goby	Sandbanks		0.917
Coates et al. 2016, Short-term effects of fishery exclusion in offshore wind farms, Fisheries Research, 179, 131-138	Round goby	Sandbanks		1.003
Coates et al. 2016, Short-term effects of fishery exclusion in offshore wind farms, Fisheries Research, 179, 131-138	Round goby	Sandbanks		2.245
Coates et al. 2016, Short-term effects of fishery exclusion in offshore wind farms, Fisheries Research, 179, 131-138	Round goby	Sandbanks		0.226
Coates et al. 2016, Short-term effects of fishery exclusion in offshore wind farms, Fisheries Research, 179, 131-138	Round goby	Sandbanks		0.838
Coates et al. 2016, Short-term effects of fishery exclusion in offshore wind farms, Fisheries Research, 179, 131-138	Round goby	Sandbanks		1.090
Coates et al. 2016, Short-term effects of fishery exclusion in offshore wind farms, Fisheries Research, 179, 131-138	Round goby	Sandbanks		1.007
Coates et al. 2016, Short-term effects of fishery exclusion in offshore wind farms, Fisheries Research, 179, 131-138	Round goby	Sandbanks		0.744
Coates et al. 2016, Short-term effects of fishery exclusion in offshore wind farms, Fisheries Research, 179, 131-138	Round goby	Sandbanks		1.221
Coates et al. 2016, Short-term effects of fishery exclusion in offshore wind farms, Fisheries Research, 179, 131-138	Round goby	Sandbanks		1.012
Coates et al. 2016, Short-term effects of fishery exclusion in offshore wind farms, Fisheries Research, 179, 131-138	Round goby	Sandbanks		0.957
Coates et al. 2016, Short-term effects of fishery exclusion in offshore wind farms, Fisheries Research, 179, 131-138	Round goby	Sandbanks		1.000

Coates et al. 2016, Short-term effects of fishery exclusion in offshore wind farms, Fisheries Research, 179, 131-138	Round goby	Sandbanks		0.896
Coates et al. 2016, Short-term effects of fishery exclusion in offshore wind farms, Fisheries Research, 179, 131-138	Round goby	Sandbanks		0.938
Coates et al. 2016, Short-term effects of fishery exclusion in offshore wind farms, Fisheries Research, 179, 131-138	Round goby	Sandbanks		0.886
Coates et al. 2016, Short-term effects of fishery exclusion in offshore wind farms, Fisheries Research, 179, 131-138	Round goby	Sandbanks		0.964
Coates et al. 2016, Short-term effects of fishery exclusion in offshore wind farms, Fisheries Research, 179, 131-138	Round goby	Sandbanks		0.976
Coates et al. 2016, Short-term effects of fishery exclusion in offshore wind farms, Fisheries Research, 179, 131-138	Round goby	Sandbanks		1.111
Coates et al. 2016, Short-term effects of fishery exclusion in offshore wind farms, Fisheries Research, 179, 131-138	Round goby	Sandbanks		1.033
Coates et al. 2016, Short-term effects of fishery exclusion in offshore wind farms, Fisheries Research, 179, 131-138	Round goby	Sandbanks		0.962
Coates et al. 2016, Short-term effects of fishery exclusion in offshore wind farms, Fisheries Research, 179, 131-138	Round goby	Sandbanks		0.982
Coates et al. 2016, Short-term effects of fishery exclusion in offshore wind farms, Fisheries Research, 179, 131-138	Round goby	Sandbanks		0.970
Coates et al. 2016, Short-term effects of fishery exclusion in offshore wind farms, Fisheries Research, 179, 131-138	Round goby	Sandbanks		0.981
Coates et al. 2016, Short-term effects of fishery exclusion in offshore wind farms, Fisheries Research, 179, 131-138	Round goby	Sandbanks		0.987
Coates et al. 2016, Short-term effects of fishery exclusion in offshore wind farms, Fisheries Research, 179, 131-138	Round goby	Sandbanks		1.001
Coates et al. 2016, Short-term effects of fishery exclusion in offshore wind farms, Fisheries Research, 179, 131-138	Round goby	Sandbanks		0.992
Coates et al. 2016, Short-term effects of fishery exclusion in offshore wind farms, Fisheries Research, 179, 131-138	Round goby	Sandbanks		0.994
Coates et al. 2016, Short-term effects of fishery exclusion in offshore wind farms, Fisheries Research, 179, 131-138	Round goby	Sandbanks		5.000
Coates et al. 2016, Short-term effects of fishery exclusion in offshore wind farms, Fisheries Research, 179, 131-138	Round goby	Sandbanks		0.000
Coates et al. 2016, Short-term effects of fishery exclusion in offshore wind farms, Fisheries Research, 179, 131-138	Round goby	Sandbanks		0.001
Coates et al. 2016, Short-term effects of fishery exclusion in offshore wind farms, Fisheries Research, 179, 131-138	Round goby	Sandbanks		0.000
Coates et al. 2016, Short-term effects of fishery exclusion in offshore wind farms, Fisheries Research, 179, 131-138	Round goby	Sandbanks		0.000
Coates et al. 2016, Short-term effects of fishery exclusion in offshore wind farms, Fisheries Research, 179, 131-138	Round goby	Sandbanks		0.000
Coates et al. 2016, Short-term effects of fishery exclusion in offshore wind farms, Fisheries Research, 179, 131-138	Round goby	Sandbanks		0.000
Coates et al. 2016, Short-term effects of fishery exclusion in offshore wind farms, Fisheries Research, 179, 131-138	Round goby	Sandbanks		0.794
Coates et al. 2016, Short-term effects of fishery exclusion in offshore wind farms, Fisheries Research, 179, 131-138	Round goby	Sandbanks		0.731
Coates et al. 2016, Short-term effects of fishery exclusion in offshore wind farms, Fisheries Research, 179, 131-138	Round goby	Sandbanks		0.767
Coates et al. 2016, Short-term effects of fishery exclusion in offshore wind farms, Fisheries Research, 179, 131-138	Round goby	Sandbanks		0.633
Coates et al. 2016, Short-term effects of fishery exclusion in offshore wind farms, Fisheries Research, 179, 131-138	Round goby	Sandbanks		0.710
Coates et al. 2016, Short-term effects of fishery exclusion in offshore wind farms, Fisheries Research, 179, 131-138	Round goby	Sandbanks		0.480
Coates et al. 2016, Short-term effects of fishery exclusion in offshore wind farms, Fisheries Research, 179, 131-138	Round goby	Sandbanks		0.877
Coates et al. 2016, Short-term effects of fishery exclusion in offshore wind farms, Fisheries Research, 179, 131-138	Round goby	Sandbanks		0.909
Coates et al. 2016, Short-term effects of fishery exclusion in offshore wind farms, Fisheries Research, 179, 131-138	Round goby	Sandbanks		0.248
Coates et al. 2016, Short-term effects of fishery exclusion in offshore wind farms, Fisheries Research, 179, 131-138	Round goby	Sandbanks		0.354
Coates et al. 2016, Short-term effects of fishery exclusion in offshore wind farms, Fisheries Research, 179, 131-138	Round goby	Sandbanks		0.919
Coates et al. 2016, Short-term effects of fishery exclusion in offshore wind farms, Fisheries Research, 179, 131-138	Round goby	Sandbanks		0.110
Coates et al. 2016, Short-term effects of fishery exclusion in offshore wind farms, Fisheries Research, 179, 131-138	Round goby	Sandbanks		0.973
Coates et al. 2016, Short-term effects of fishery exclusion in offshore wind farms, Fisheries Research, 179, 131-138	Round goby	Sandbanks		0.059
Coates et al. 2016, Short-term effects of fishery exclusion in offshore wind farms, Fisheries Research, 179, 131-138	Round goby	Sandbanks		0.909
Coates et al. 2016, Short-term effects of fishery exclusion in offshore wind farms, Fisheries Research, 179, 131-138	Round goby	Sandbanks		0.551
Coates et al. 2016, Short-term effects of fishery exclusion in offshore wind farms, Fisheries Research, 179, 131-138	Round goby	Sandbanks		0.711
European Invasive Species impact database, No 62; Nurkse, K., Kotta, J., Orav-Kotta, H., Ojaveer, H. (2016) A successful non-native predator, round goby, in the Baltic Sea: generalist feeding strategy, diverse diet and high prey consumption. Hydrobiologia 777, 271–281.	Round goby	Sandbanks		0.361
European Invasive Species impact database, No 63; Nurkse, K., Kotta, J., Orav-Kotta, H., Ojaveer, H. (2016) A successful non-native predator, round goby, in the Baltic Sea: generalist feeding strategy, diverse diet and high prey consumption. Hydrobiologia 777, 271–281.	Round goby	Sandbanks		0.514
Nurkse, K., Kotta, J., Orav-Kotta, H., Ojaveer, H. (2016) A successful non-native predator, round goby, in the Baltic Sea: generalist feeding strategy, diverse diet and high prey consumption. Hydrobiologia 777, 271–281.	Round goby	Sandbanks		0.281
Nurkse, K., Kotta, J., Orav-Kotta, H., Ojaveer, H. (2016) A successful non-native predator, round goby, in the Baltic Sea: generalist feeding strategy, diverse diet and high prey consumption. Hydrobiologia 777, 271–281.	Round goby	Sandbanks		0.324
Nurkse, K., Kotta, J., Rätsep, M., Kotta, I., Kreitsberg, R., 2018. Experimental evaluation of the effects of the novel predators, round goby and mud crab on benthic invertebrates in the Gulf of Riga, Baltic Sea. J. Mar. Biol. Assoc. U. K. 98, 25–31.	Round goby and Mud crab	Sandbanks		0.948
Kautsky, H. 1992. The Impact of Pulp-Mill Effluents on Phytobenthic Communities in the Baltic Sea. Ambio, 21 (4): 308-313.	Nutrient input	Sandbanks		0.417
Kautsky, H. 1992. The Impact of Pulp-Mill Effluents on Phytobenthic Communities in the Baltic Sea. Ambio, 21 (4): 308-313.	Nutrient input	Sandbanks		0.412

Anger, K. 1977. Benthic Invertebrates as Indicators of Organic Pollution in the Western Baltic Sea. Internationale Revue der gesamten Hydrobiologie, 62(2): 245-254.	Nutrient input	Sandbanks		0.221
Anger, K. 1977. Benthic Invertebrates as Indicators of Organic Pollution in the Western Baltic Sea. Internationale Revue der gesamten Hydrobiologie, 62(2): 245-254.	Nutrient input	Sandbanks		0.124
Anger, K. 1977. Benthic Invertebrates as Indicators of Organic Pollution in the Western Baltic Sea. Internationale Revue der gesamten Hydrobiologie, 62(2): 245-254.	Nutrient input	Sandbanks		0.158
Anger, K. 1977. Benthic Invertebrates as Indicators of Organic Pollution in the Western Baltic Sea. Internationale Revue der gesamten Hydrobiologie, 62(2): 245-254.	Nutrient input	Sandbanks		0.080
Anger, K. 1977. Benthic Invertebrates as Indicators of Organic Pollution in the Western Baltic Sea. Internationale Revue der gesamten Hydrobiologie, 62(2): 245-254.	Nutrient input	Sandbanks		5.000
Anger, K. 1977. Benthic Invertebrates as Indicators of Organic Pollution in the Western Baltic Sea. Internationale Revue der gesamten Hydrobiologie, 62(2): 245-254.	Nutrient input	Sandbanks		3.791
Anger, K. 1977. Benthic Invertebrates as Indicators of Organic Pollution in the Western Baltic Sea. Internationale Revue der gesamten Hydrobiologie, 62(2): 245-254.	Nutrient input	Sandbanks		5.000
Anger, K. 1977. Benthic Invertebrates as Indicators of Organic Pollution in the Western Baltic Sea. Internationale Revue der gesamten Hydrobiologie, 62(2): 245-254.	Nutrient input	Sandbanks		3.658
Anger, K. 1977. Benthic Invertebrates as Indicators of Organic Pollution in the Western Baltic Sea. Internationale Revue der gesamten Hydrobiologie, 62(2): 245-254.	Nutrient input	Sandbanks		0.022
Anger, K. 1977. Benthic Invertebrates as Indicators of Organic Pollution in the Western Baltic Sea. Internationale Revue der gesamten Hydrobiologie, 62(2): 245-254.	Nutrient input	Sandbanks		0.061
Anger, K. 1977. Benthic Invertebrates as Indicators of Organic Pollution in the Western Baltic Sea. Internationale Revue der gesamten Hydrobiologie, 62(2): 245-254.	Nutrient input	Sandbanks		0.036
Anger, K. 1977. Benthic Invertebrates as Indicators of Organic Pollution in the Western Baltic Sea. Internationale Revue der gesamten Hydrobiologie, 62(2): 245-254.	Nutrient input	Sandbanks		0.025
Anger, K. 1977. Benthic Invertebrates as Indicators of Organic Pollution in the Western Baltic Sea. Internationale Revue der gesamten Hydrobiologie, 62(2): 245-254.	Nutrient input	Sandbanks		0.025
Anger, K. 1977. Benthic Invertebrates as Indicators of Organic Pollution in the Western Baltic Sea. Internationale Revue der gesamten Hydrobiologie, 62(2): 245-254.	Nutrient input	Sandbanks		0.629
Anger, K. 1977. Benthic Invertebrates as Indicators of Organic Pollution in the Western Baltic Sea. Internationale Revue der gesamten Hydrobiologie, 62(2): 245-254.	Nutrient input	Sandbanks		0.857
Anger, K. 1977. Benthic Invertebrates as Indicators of Organic Pollution in the Western Baltic Sea. Internationale Revue der gesamten Hydrobiologie, 62(2): 245-254.	Nutrient input	Sandbanks		0.192
Anger, K. 1977. Benthic Invertebrates as Indicators of Organic Pollution in the Western Baltic Sea. Internationale Revue der gesamten Hydrobiologie, 62(2): 245-254.	Nutrient input	Sandbanks		0.404
Anger, K. 1977. Benthic Invertebrates as Indicators of Organic Pollution in the Western Baltic Sea. Internationale Revue der gesamten Hydrobiologie, 62(2): 245-254.	Nutrient input	Sandbanks		0.101
Anger, K. 1977. Benthic Invertebrates as Indicators of Organic Pollution in the Western Baltic Sea. Internationale Revue der gesamten Hydrobiologie, 62(2): 245-254.	Nutrient input	Sandbanks		0.044
Anger, K. 1977. Benthic Invertebrates as Indicators of Organic Pollution in the Western Baltic Sea. Internationale Revue der gesamten Hydrobiologie, 62(2): 245-254.	Nutrient input	Sandbanks		0.053
Anger, K. 1977. Benthic Invertebrates as Indicators of Organic Pollution in the Western Baltic Sea. Internationale Revue der gesamten Hydrobiologie, 62(2): 245-254.	Nutrient input	Sandbanks		0.158
Anger, K. 1977. Benthic Invertebrates as Indicators of Organic Pollution in the Western Baltic Sea. Internationale Revue der gesamten Hydrobiologie, 62(2): 245-254.	Nutrient input	Sandbanks		0.162
Anger, K. 1977. Benthic Invertebrates as Indicators of Organic Pollution in the Western Baltic Sea. Internationale Revue der gesamten Hydrobiologie, 62(2): 245-254.	Nutrient input	Sandbanks		5.000
Anger, K. 1977. Benthic Invertebrates as Indicators of Organic Pollution in the Western Baltic Sea. Internationale Revue der gesamten Hydrobiologie, 62(2): 245-254.	Nutrient input	Sandbanks		5.000
Anger, K. 1977. Benthic Invertebrates as Indicators of Organic Pollution in the Western Baltic Sea. Internationale Revue der gesamten Hydrobiologie, 62(2): 245-254.	Nutrient input	Sandbanks		0.088
Anger, K. 1977. Benthic Invertebrates as Indicators of Organic Pollution in the Western Baltic Sea. Internationale Revue der gesamten Hydrobiologie, 62(2): 245-254.	Nutrient input	Sandbanks		0.012
Anger, K. 1977. Benthic Invertebrates as Indicators of Organic Pollution in the Western Baltic Sea. Internationale Revue der gesamten Hydrobiologie, 62(2): 245-254.	Nutrient input	Sandbanks		0.084
Anger, K. 1977. Benthic Invertebrates as Indicators of Organic Pollution in the Western Baltic Sea. Internationale Revue der gesamten Hydrobiologie, 62(2): 245-254.	Nutrient input	Sandbanks		0.087
Anger, K. 1977. Benthic Invertebrates as Indicators of Organic Pollution in the Western Baltic Sea. Internationale Revue der gesamten Hydrobiologie, 62(2): 245-254.	Nutrient input	Sandbanks		0.086

Anger, K. 1977. Benthic Invertebrates as Indicators of Organic Pollution in the Western Baltic Sea. Internationale Revue der gesamten Hydrobiologie, 62(2): 245-254.	Nutrient input	Sandbanks		0.088
Anger, K. 1977. Benthic Invertebrates as Indicators of Organic Pollution in the Western Baltic Sea. Internationale Revue der gesamten Hydrobiologie, 62(2): 245-254.	Nutrient input	Sandbanks		0.233
Anger, K. 1977. Benthic Invertebrates as Indicators of Organic Pollution in the Western Baltic Sea. Internationale Revue der gesamten Hydrobiologie, 62(2): 245-254.	Nutrient input	Sandbanks		0.039
Anger, K. 1977. Benthic Invertebrates as Indicators of Organic Pollution in the Western Baltic Sea. Internationale Revue der gesamten Hydrobiologie, 62(2): 245-254.	Nutrient input	Sandbanks		0.143
Anger, K. 1977. Benthic Invertebrates as Indicators of Organic Pollution in the Western Baltic Sea. Internationale Revue der gesamten Hydrobiologie, 62(2): 245-254.	Nutrient input	Sandbanks		0.039
Anger, K. 1977. Benthic Invertebrates as Indicators of Organic Pollution in the Western Baltic Sea. Internationale Revue der gesamten Hydrobiologie, 62(2): 245-254.	Nutrient input	Sandbanks		0.087
Anger, K. 1977. Benthic Invertebrates as Indicators of Organic Pollution in the Western Baltic Sea. Internationale Revue der gesamten Hydrobiologie, 62(2): 245-254.	Nutrient input	Sandbanks		0.123
Anger, K. 1977. Benthic Invertebrates as Indicators of Organic Pollution in the Western Baltic Sea. Internationale Revue der gesamten Hydrobiologie, 62(2): 245-254.	Nutrient input	Sandbanks		0.081
Anger, K. 1977. Benthic Invertebrates as Indicators of Organic Pollution in the Western Baltic Sea. Internationale Revue der gesamten Hydrobiologie, 62(2): 245-254.	Nutrient input	Sandbanks		0.118
Anger, K. 1977. Benthic Invertebrates as Indicators of Organic Pollution in the Western Baltic Sea. Internationale Revue der gesamten Hydrobiologie, 62(2): 245-254.	Nutrient input	Sandbanks		0.007
Anger, K. 1977. Benthic Invertebrates as Indicators of Organic Pollution in the Western Baltic Sea. Internationale Revue der gesamten Hydrobiologie, 62(2): 245-254.	Nutrient input	Sandbanks		0.005
Anger, K. 1977. Benthic Invertebrates as Indicators of Organic Pollution in the Western Baltic Sea. Internationale Revue der gesamten Hydrobiologie, 62(2): 245-254.	Nutrient input	Sandbanks		0.056
Anger, K. 1977. Benthic Invertebrates as Indicators of Organic Pollution in the Western Baltic Sea. Internationale Revue der gesamten Hydrobiologie, 62(2): 245-254.	Nutrient input	Sandbanks		0.053
Anger, K. 1977. Benthic Invertebrates as Indicators of Organic Pollution in the Western Baltic Sea. Internationale Revue der gesamten Hydrobiologie, 62(2): 245-254.	Nutrient input	Sandbanks		0.009
Anger, K. 1977. Benthic Invertebrates as Indicators of Organic Pollution in the Western Baltic Sea. Internationale Revue der gesamten Hydrobiologie, 62(2): 245-254.	Nutrient input	Sandbanks		0.045
Anger, K. 1977. Benthic Invertebrates as Indicators of Organic Pollution in the Western Baltic Sea. Internationale Revue der gesamten Hydrobiologie, 62(2): 245-254.	Nutrient input	Sandbanks		0.010
Anger, K. 1977. Benthic Invertebrates as Indicators of Organic Pollution in the Western Baltic Sea. Internationale Revue der gesamten Hydrobiologie, 62(2): 245-254.	Nutrient input	Sandbanks		0.441
Anger, K. 1977. Benthic Invertebrates as Indicators of Organic Pollution in the Western Baltic Sea. Internationale Revue der gesamten Hydrobiologie, 62(2): 245-254.	Nutrient input	Sandbanks		1.002
Anger, K. 1977. Benthic Invertebrates as Indicators of Organic Pollution in the Western Baltic Sea. Internationale Revue der gesamten Hydrobiologie, 62(2): 245-254.	Nutrient input	Sandbanks		0.569
Anger, K. 1977. Benthic Invertebrates as Indicators of Organic Pollution in the Western Baltic Sea. Internationale Revue der gesamten Hydrobiologie, 62(2): 245-254.	Nutrient input	Sandbanks		3.812
Anger, K. 1977. Benthic Invertebrates as Indicators of Organic Pollution in the Western Baltic Sea. Internationale Revue der gesamten Hydrobiologie, 62(2): 245-254.	Nutrient input	Sandbanks		0.715
Anger, K. 1977. Benthic Invertebrates as Indicators of Organic Pollution in the Western Baltic Sea. Internationale Revue der gesamten Hydrobiologie, 62(2): 245-254.	Nutrient input	Sandbanks		0.057
Anger, K. 1977. Benthic Invertebrates as Indicators of Organic Pollution in the Western Baltic Sea. Internationale Revue der gesamten Hydrobiologie, 62(2): 245-254.	Nutrient input	Sandbanks		0.039
Anger, K. 1977. Benthic Invertebrates as Indicators of Organic Pollution in the Western Baltic Sea. Internationale Revue der gesamten Hydrobiologie, 62(2): 245-254.	Nutrient input	Sandbanks		0.277
Anger, K. 1977. Benthic Invertebrates as Indicators of Organic Pollution in the Western Baltic Sea. Internationale Revue der gesamten Hydrobiologie, 62(2): 245-254.	Nutrient input	Sandbanks		0.254
Anger, K. 1977. Benthic Invertebrates as Indicators of Organic Pollution in the Western Baltic Sea. Internationale Revue der gesamten Hydrobiologie, 62(2): 245-254.	Nutrient input	Sandbanks		4.930
Anger, K. 1977. Benthic Invertebrates as Indicators of Organic Pollution in the Western Baltic Sea. Internationale Revue der gesamten Hydrobiologie, 62(2): 245-254.	Nutrient input	Sandbanks		5.000
Anger, K. 1977. Benthic Invertebrates as Indicators of Organic Pollution in the Western Baltic Sea. Internationale Revue der gesamten Hydrobiologie, 62(2): 245-254.	Nutrient input	Sandbanks		5.000
Anger, K. 1977. Benthic Invertebrates as Indicators of Organic Pollution in the Western Baltic Sea. Internationale Revue der gesamten Hydrobiologie, 62(2): 245-254.	Nutrient input	Sandbanks		5.000

Anger, K. 1977. Benthic Invertebrates as Indicators of Organic Pollution in the Western Baltic Sea. Internationale Revue der gesamten Hydrobiologie, 62(2): 245-254.	Nutrient input	Sandbanks		0.047
Anger, K. 1977. Benthic Invertebrates as Indicators of Organic Pollution in the Western Baltic Sea. Internationale Revue der gesamten Hydrobiologie, 62(2): 245-254.	Nutrient input	Sandbanks		0.050
Anger, K. 1977. Benthic Invertebrates as Indicators of Organic Pollution in the Western Baltic Sea. Internationale Revue der gesamten Hydrobiologie, 62(2): 245-254.	Nutrient input	Sandbanks		0.036
Anger, K. 1977. Benthic Invertebrates as Indicators of Organic Pollution in the Western Baltic Sea. Internationale Revue der gesamten Hydrobiologie, 62(2): 245-254.	Nutrient input	Sandbanks		0.032
Anger, K. 1977. Benthic Invertebrates as Indicators of Organic Pollution in the Western Baltic Sea. Internationale Revue der gesamten Hydrobiologie, 62(2): 245-254.	Nutrient input	Sandbanks		3.597
Anger, K. 1977. Benthic Invertebrates as Indicators of Organic Pollution in the Western Baltic Sea. Internationale Revue der gesamten Hydrobiologie, 62(2): 245-254.	Nutrient input	Sandbanks		1.860
Anger, K. 1977. Benthic Invertebrates as Indicators of Organic Pollution in the Western Baltic Sea. Internationale Revue der gesamten Hydrobiologie, 62(2): 245-254.	Nutrient input	Sandbanks		3.182
Anger, K. 1977. Benthic Invertebrates as Indicators of Organic Pollution in the Western Baltic Sea. Internationale Revue der gesamten Hydrobiologie, 62(2): 245-254.	Nutrient input	Sandbanks		2.150
Coates et al. 2016, Short-term effects of fishery exclusion in offshore wind farms, Fisheries Research, 179, 131-138	Nutrient input	Sandbanks		1.441
Coates et al. 2016, Short-term effects of fishery exclusion in offshore wind farms, Fisheries Research, 179, 131-138	Nutrient input	Sandbanks		0.859
Coates et al. 2016, Short-term effects of fishery exclusion in offshore wind farms, Fisheries Research, 179, 131-138	Nutrient input	Sandbanks		0.909
Coates et al. 2016, Short-term effects of fishery exclusion in offshore wind farms, Fisheries Research, 179, 131-138	Nutrient input	Sandbanks		0.996
Coates et al. 2016, Short-term effects of fishery exclusion in offshore wind farms, Fisheries Research, 179, 131-138	Nutrient input	Sandbanks		1.001
Coates et al. 2016, Short-term effects of fishery exclusion in offshore wind farms, Fisheries Research, 179, 131-138	Nutrient input	Sandbanks		0.999
Coates et al. 2016, Short-term effects of fishery exclusion in offshore wind farms, Fisheries Research, 179, 131-138	Nutrient input	Sandbanks		0.324
Coates et al. 2016, Short-term effects of fishery exclusion in offshore wind farms, Fisheries Research, 179, 131-138	Nutrient input	Sandbanks		0.685
Coates et al. 2016, Short-term effects of fishery exclusion in offshore wind farms, Fisheries Research, 179, 131-138	Nutrient input	Sandbanks		1.007
Coates et al. 2016, Short-term effects of fishery exclusion in offshore wind farms, Fisheries Research, 179, 131-138	Nutrient input	Sandbanks		5.000
Nurkse, K., Kotta, J., Rätsep, M., Kotta, I., Kreitsberg, R., 2018. Experimental evaluation of the effects of the novel predators, round goby and mud crab on benthic invertebrates in the Gulf of Riga, Baltic Sea. J. Mar. Biol. Assoc. U. K. 98, 25–31.	Mud crab	Sandbanks		0.923
Kotta, J., Wernberg, T., Jänes, H., Kotta, I., Nurkse, K., Pärnoja, M., & Orav-Kotta, H. (2018). Novel crab predator causes marine ecosystem regime shift. Scientific reports, 8(1), 4956.	Mud crab	Sandbanks		0.319
Lokko, K.; Kotta, J.; Orav-Kotta, H.; Nurkse, K.; Pärnoja, M. (2018). Introduction of a functionally novel consumer to a low diversity system: effects of the mud crab Rhithropanopeus harrisii on meiobenthos. Estuarine Coastal and Shelf Science, x−x. DOI: 10.1016/j.ecss.2015.11.017.	Mud crab	Sandbanks		1.244
Lokko, K.; Kotta, J.; Orav-Kotta, H.; Nurkse, K.; Pärnoja, M. (2018). Introduction of a functionally novel consumer to a low diversity system: effects of the mud crab Rhithropanopeus harrisii on meiobenthos. Estuarine Coastal and Shelf Science, x−x. DOI: 10.1016/j.ecss.2015.11.017.	Mud crab	Sandbanks		1.003
Lokko, K.; Kotta, J.; Orav-Kotta, H.; Nurkse, K.; Pärnoja, M. (2018). Introduction of a functionally novel consumer to a low diversity system: effects of the mud crab Rhithropanopeus harrisii on meiobenthos. Estuarine Coastal and Shelf Science, x−x. DOI: 10.1016/j.ecss.2015.11.017.	Mud crab	Sandbanks		1.184
Lokko, K.; Kotta, J.; Orav-Kotta, H.; Nurkse, K.; Pärnoja, M. (2018). Introduction of a functionally novel consumer to a low diversity system: effects of the mud crab Rhithropanopeus harrisii on meiobenthos. Estuarine Coastal and Shelf Science, x−x. DOI: 10.1016/j.ecss.2015.11.017.	Mud crab	Sandbanks		1.116
Lokko, K.; Kotta, J.; Orav-Kotta, H.; Nurkse, K.; Pärnoja, M. (2018). Introduction of a functionally novel consumer to a low diversity system: effects of the mud crab Rhithropanopeus harrisii on meiobenthos. Estuarine Coastal and Shelf Science, x−x. DOI: 10.1016/j.ecss.2015.11.017.	Mud crab	Sandbanks		0.491
Lokko, K.; Kotta, J.; Orav-Kotta, H.; Nurkse, K.; Pärnoja, M. (2018). Introduction of a functionally novel consumer to a low diversity system: effects of the mud crab Rhithropanopeus harrisii on meiobenthos. Estuarine Coastal and Shelf Science, x−x. DOI: 10.1016/j.ecss.2015.11.017.	Mud crab	Sandbanks		1.083
Lokko, K.; Kotta, J.; Orav-Kotta, H.; Nurkse, K.; Pärnoja, M. (2018). Introduction of a functionally novel consumer to a low diversity system: effects of the mud crab Rhithropanopeus harrisii on meiobenthos. Estuarine Coastal and Shelf Science, x−x. DOI: 10.1016/j.ecss.2015.11.017.	Mud crab	Sandbanks		0.736
Lokko, K.; Kotta, J.; Orav-Kotta, H.; Nurkse, K.; Pärnoja, M. (2018). Introduction of a functionally novel consumer to a low diversity system: effects of the mud crab Rhithropanopeus harrisii on meiobenthos. Estuarine Coastal and Shelf Science, x−x. DOI: 10.1016/j.ecss.2015.11.017.	Mud crab	Sandbanks		0.720
Lokko, K.; Kotta, J.; Orav-Kotta, H.; Nurkse, K.; Pärnoja, M. (2018). Introduction of a functionally novel consumer to a low diversity system: effects of the mud crab Rhithropanopeus harrisii on meiobenthos. Estuarine Coastal and Shelf Science, x−x. DOI: 10.1016/j.ecss.2015.11.017.	Mud crab	Sandbanks		5.000

Lokko, K.; Kotta, J.; Orav-Kotta, H.; Nurkse, K.; Pärnoja, M. (2018). Introduction of a functionally novel consumer to a low diversity system: effects of the mud crab Rhithropanopeus harrisii on meiobenthos. Estuarine Coastal and Shelf Science, x−x. DOI: 10.1016/j.ecss.2015.11.017.	Mud crab	Sandbanks		0.846
Lokko, K.; Kotta, J.; Orav-Kotta, H.; Nurkse, K.; Pärnoja, M. (2018). Introduction of a functionally novel consumer to a low diversity system: effects of the mud crab Rhithropanopeus harrisii on meiobenthos. Estuarine Coastal and Shelf Science, x−x. DOI: 10.1016/j.ecss.2015.11.017.	Mud crab	Sandbanks		0.226
Lokko, K.; Kotta, J.; Orav-Kotta, H.; Nurkse, K.; Pärnoja, M. (2018). Introduction of a functionally novel consumer to a low diversity system: effects of the mud crab Rhithropanopeus harrisii on meiobenthos. Estuarine Coastal and Shelf Science, x−x. DOI: 10.1016/j.ecss.2015.11.017.	Mud crab	Sandbanks		0.632
Bergström, L., Sundqvist, F., & Bergström, U. (2013). Effects of an offshore wind farm on temporal and spatial patterns in the demersal fish community. Marine Ecology Progress Series, 485, 199-210.	Wind park	Sandbanks		2.370
Bergström, L., Sundqvist, F., & Bergström, U. (2013). Effects of an offshore wind farm on temporal and spatial patterns in the demersal fish community. Marine Ecology Progress Series, 485, 199-210.	Wind park	Sandbanks		0.248
Bergström, L., Sundqvist, F., & Bergström, U. (2013). Effects of an offshore wind farm on temporal and spatial patterns in the demersal fish community. Marine Ecology Progress Series, 485, 199-210.	Wind park	Sandbanks		1.798
Andersson & Ohman, 2010, Fish and sessile assemblages associated with wind-turbine constructions in the Baltic Sea, Marine and Freshwater Research, 2010, 61, 642–651	Wind park	Sandbanks		5.000
Andersson & Ohman, 2010, Fish and sessile assemblages associated with wind-turbine constructions in the Baltic Sea, Marine and Freshwater Research, 2010, 61, 642–651	Wind park	Sandbanks		2.291
Andersson & Ohman, 2010, Fish and sessile assemblages associated with wind-turbine constructions in the Baltic Sea, Marine and Freshwater Research, 2010, 61, 642–651	Wind park	Sandbanks		4.230
Andersson & Ohman, 2010, Fish and sessile assemblages associated with wind-turbine constructions in the Baltic Sea, Marine and Freshwater Research, 2010, 61, 642–653	Wind park	Sandbanks		5.000
Andersson & Ohman, 2010, Fish and sessile assemblages associated with wind-turbine constructions in the Baltic Sea, Marine and Freshwater Research, 2010, 61, 642–653	Wind park	Sandbanks		2.977
Andersson & Ohman, 2010, Fish and sessile assemblages associated with wind-turbine constructions in the Baltic Sea, Marine and Freshwater Research, 2010, 61, 642–653	Wind park	Sandbanks		0.845
Andersson & Ohman, 2010, Fish and sessile assemblages associated with wind-turbine constructions in the Baltic Sea, Marine and Freshwater Research, 2010, 61, 642–653	Wind park	Sandbanks		5.000
Andersson & Ohman, 2010, Fish and sessile assemblages associated with wind-turbine constructions in the Baltic Sea, Marine and Freshwater Research, 2010, 61, 642–653	Wind park	Sandbanks		2.868
Andersson & Ohman, 2010, Fish and sessile assemblages associated with wind-turbine constructions in the Baltic Sea, Marine and Freshwater Research, 2010, 61, 642–653	Wind park	Sandbanks		0.991
Andersson & Ohman, 2010, Fish and sessile assemblages associated with wind-turbine constructions in the Baltic Sea, Marine and Freshwater Research, 2010, 61, 642–653	Wind park	Sandbanks		5.000
Andersson & Ohman, 2010, Fish and sessile assemblages associated with wind-turbine constructions in the Baltic Sea, Marine and Freshwater Research, 2010, 61, 642–653	Wind park	Sandbanks		3.279
Andersson & Ohman, 2010, Fish and sessile assemblages associated with wind-turbine constructions in the Baltic Sea, Marine and Freshwater Research, 2010, 61, 642–653	Wind park	Sandbanks		5.000
Andersson & Ohman, 2010, Fish and sessile assemblages associated with wind-turbine constructions in the Baltic Sea, Marine and Freshwater Research, 2010, 61, 642–653	Wind park	Sandbanks		0.169
Andersson & Ohman, 2010, Fish and sessile assemblages associated with wind-turbine constructions in the Baltic Sea, Marine and Freshwater Research, 2010, 61, 642–653	Wind park	Sandbanks		1.498
Andersson & Ohman, 2010, Fish and sessile assemblages associated with wind-turbine constructions in the Baltic Sea, Marine and Freshwater Research, 2010, 61, 642–653	Wind park	Sandbanks		5.000
Andersson & Ohman, 2010, Fish and sessile assemblages associated with wind-turbine constructions in the Baltic Sea, Marine and Freshwater Research, 2010, 61, 642–653	Wind park	Sandbanks		5.000
Andersson & Ohman, 2010, Fish and sessile assemblages associated with wind-turbine constructions in the Baltic Sea, Marine and Freshwater Research, 2010, 61, 642–653	Wind park	Sandbanks		3.757
Andersson & Ohman, 2010, Fish and sessile assemblages associated with wind-turbine constructions in the Baltic Sea, Marine and Freshwater Research, 2010, 61, 642–653	Wind park	Sandbanks		5.000
Andersson & Ohman, 2010, Fish and sessile assemblages associated with wind-turbine constructions in the Baltic Sea, Marine and Freshwater Research, 2010, 61, 642–653	Wind park	Sandbanks		5.000
Andersson & Ohman, 2010, Fish and sessile assemblages associated with wind-turbine constructions in the Baltic Sea, Marine and Freshwater Research, 2010, 61, 642–653	Wind park	Sandbanks		5.000
Andersson & Ohman, 2010, Fish and sessile assemblages associated with wind-turbine constructions in the Baltic Sea, Marine and Freshwater Research, 2010, 61, 642–653	Wind park	Sandbanks		1.000

Andersson & Ohman, 2010, Fish and sessile assemblages associated with wind-turbine constructions in the Baltic Sea, Marine and Freshwater Research, 2010, 61, 642–653	Wind park	Sandbanks		3.820
Andersson & Ohman, 2010, Fish and sessile assemblages associated with wind-turbine constructions in the Baltic Sea, Marine and Freshwater Research, 2010, 61, 642–653	Wind park	Sandbanks		1.788
Andersson & Ohman, 2010, Fish and sessile assemblages associated with wind-turbine constructions in the Baltic Sea, Marine and Freshwater Research, 2010, 61, 642–653	Wind park	Sandbanks		1.000
Andersson & Ohman, 2010, Fish and sessile assemblages associated with wind-turbine constructions in the Baltic Sea, Marine and Freshwater Research, 2010, 61, 642–653	Wind park	Sandbanks		0.888
Andersson & Ohman, 2010, Fish and sessile assemblages associated with wind-turbine constructions in the Baltic Sea, Marine and Freshwater Research, 2010, 61, 642–653	Wind park	Sandbanks		0.646
Andersson & Ohman, 2010, Fish and sessile assemblages associated with wind-turbine constructions in the Baltic Sea, Marine and Freshwater Research, 2010, 61, 642–653	Wind park	Sandbanks		1.000
Andersson & Ohman, 2010, Fish and sessile assemblages associated with wind-turbine constructions in the Baltic Sea, Marine and Freshwater Research, 2010, 61, 642–653	Wind park	Sandbanks		1.059
Andersson & Ohman, 2010, Fish and sessile assemblages associated with wind-turbine constructions in the Baltic Sea, Marine and Freshwater Research, 2010, 61, 642–653	Wind park	Sandbanks		0.727
Atalah et al., 2013, Diversity of demersal and megafaunal assemblages inhabiting sandbanks of the Irish Sea, Marine Biodiversity, 43, 121–132.	Wind park	Sandbanks		1.829
Atalah et al., 2013, Diversity of demersal and megafaunal assemblages inhabiting sandbanks of the Irish Sea, Marine Biodiversity, 43, 121–132.	Wind park	Sandbanks		4.953
Atalah et al., 2013, Diversity of demersal and megafaunal assemblages inhabiting sandbanks of the Irish Sea, Marine Biodiversity, 43, 121–132.	Wind park	Sandbanks		1.419
Bergström et al., 2013, Effects of an offshore wind farm on temporal and spatial patterns in the demersal fish community, Marine Ecology Progress Series, 485, 199–210	Wind park	Sandbanks		1.462
Bergström et al., 2013, Effects of an offshore wind farm on temporal and spatial patterns in the demersal fish community, Marine Ecology Progress Series, 485, 199–210	Wind park	Sandbanks		1.209
Bergström et al., 2013, Effects of an offshore wind farm on temporal and spatial patterns in the demersal fish community, Marine Ecology Progress Series, 485, 199–210	Wind park	Sandbanks		1.045
Bergström et al., 2013, Effects of an offshore wind farm on temporal and spatial patterns in the demersal fish community, Marine Ecology Progress Series, 485, 199–210	Wind park	Sandbanks		1.061
Bergström et al., 2013, Effects of an offshore wind farm on temporal and spatial patterns in the demersal fish community, Marine Ecology Progress Series, 485, 199–210	Wind park	Sandbanks		1.681
Bergström et al., 2013, Effects of an offshore wind farm on temporal and spatial patterns in the demersal fish community, Marine Ecology Progress Series, 485, 199–210	Wind park	Sandbanks		0.962
Bergström et al., 2013, Effects of an offshore wind farm on temporal and spatial patterns in the demersal fish community, Marine Ecology Progress Series, 485, 199–210	Wind park	Sandbanks		1.275
Bergström et al., 2013, Effects of an offshore wind farm on temporal and spatial patterns in the demersal fish community, Marine Ecology Progress Series, 485, 199–210	Wind park	Sandbanks		0.839
Bergström et al., 2013, Effects of an offshore wind farm on temporal and spatial patterns in the demersal fish community, Marine Ecology Progress Series, 485, 199–210	Wind park	Sandbanks		5.000
Bergström et al., 2013, Effects of an offshore wind farm on temporal and spatial patterns in the demersal fish community, Marine Ecology Progress Series, 485, 199–210	Wind park	Sandbanks		4.354
Bergström et al., 2013, Effects of an offshore wind farm on temporal and spatial patterns in the demersal fish community, Marine Ecology Progress Series, 485, 199–210	Wind park	Sandbanks		1.472
Bergström et al., 2013, Effects of an offshore wind farm on temporal and spatial patterns in the demersal fish community, Marine Ecology Progress Series, 485, 199–210	Wind park	Sandbanks		2.130
Bergström et al., 2013, Effects of an offshore wind farm on temporal and spatial patterns in the demersal fish community, Marine Ecology Progress Series, 485, 199–210	Wind park	Sandbanks		1.486
Bergström et al., 2013, Effects of an offshore wind farm on temporal and spatial patterns in the demersal fish community, Marine Ecology Progress Series, 485, 199–210	Wind park	Sandbanks		1.287
Bergström et al., 2013, Effects of an offshore wind farm on temporal and spatial patterns in the demersal fish community, Marine Ecology Progress Series, 485, 199–210	Wind park	Sandbanks		4.399
Bergström et al., 2013, Effects of an offshore wind farm on temporal and spatial patterns in the demersal fish community, Marine Ecology Progress Series, 485, 199–210	Wind park	Sandbanks		1.511
Bergström et al., 2013, Effects of an offshore wind farm on temporal and spatial patterns in the demersal fish community, Marine Ecology Progress Series, 485, 199–210	Wind park	Sandbanks		0.621
Bergström et al., 2013, Effects of an offshore wind farm on temporal and spatial patterns in the demersal fish community, Marine Ecology Progress Series, 485, 199–210	Wind park	Sandbanks		0.209

Bergström et al., 2013, Effects of an offshore wind farm on temporal and spatial patterns in the demersal fish community, Marine Ecology Progress Series, 485, 199–210	Wind park	Sandbanks		0.307
Bergström et al., 2013, Effects of an offshore wind farm on temporal and spatial patterns in the demersal fish community, Marine Ecology Progress Series, 485, 199–210	Wind park	Sandbanks		0.270
Bergström et al., 2013, Effects of an offshore wind farm on temporal and spatial patterns in the demersal fish community, Marine Ecology Progress Series, 485, 199–210	Wind park	Sandbanks		1.647
Bergström et al., 2013, Effects of an offshore wind farm on temporal and spatial patterns in the demersal fish community, Marine Ecology Progress Series, 485, 199–210	Wind park	Sandbanks		1.770
Raoux et al. 2017, Benthic and fish aggregation inside an offshore wind farm, Ecological Indicators 72, 33-46	Wind park	Sandbanks		1.024
Raoux et al. 2017, Benthic and fish aggregation inside an offshore wind farm, Ecological Indicators 72, 33-46	Wind park	Sandbanks		1.532
Raoux et al. 2017, Benthic and fish aggregation inside an offshore wind farm, Ecological Indicators 72, 33-46	Wind park	Sandbanks		2.200
Raoux et al. 2017, Benthic and fish aggregation inside an offshore wind farm, Ecological Indicators 72, 33-46	Wind park	Sandbanks		0.965
Raoux et al. 2017, Benthic and fish aggregation inside an offshore wind farm, Ecological Indicators 72, 33-46	Wind park	Sandbanks		0.835
Raoux et al. 2017, Benthic and fish aggregation inside an offshore wind farm, Ecological Indicators 72, 33-46	Wind park	Sandbanks		0.855
Raoux et al. 2017, Benthic and fish aggregation inside an offshore wind farm, Ecological Indicators 72, 33-46	Wind park	Sandbanks		1.093
Coates et al. 2016, Short-term effects of fishery exclusion in offshore wind farms, Fisheries Research, 179, 131-138	Wind park	Sandbanks		0.866
Coates et al. 2016, Short-term effects of fishery exclusion in offshore wind farms, Fisheries Research, 179, 131-138	Wind park	Sandbanks		0.986
Coates et al. 2016, Short-term effects of fishery exclusion in offshore wind farms, Fisheries Research, 179, 131-138	Wind park	Sandbanks		1.134
Coates et al. 2016, Short-term effects of fishery exclusion in offshore wind farms, Fisheries Research, 179, 131-138	Wind park	Sandbanks		0.479
Coates et al. 2016, Short-term effects of fishery exclusion in offshore wind farms, Fisheries Research, 179, 131-138	Wind park	Sandbanks		1.183
Coates et al. 2016, Short-term effects of fishery exclusion in offshore wind farms, Fisheries Research, 179, 131-138	Wind park	Sandbanks		0.717
Coates et al. 2016, Short-term effects of fishery exclusion in offshore wind farms, Fisheries Research, 179, 131-138	Wind park	Sandbanks		2.448
Coates et al. 2016, Short-term effects of fishery exclusion in offshore wind farms, Fisheries Research, 179, 131-138	Wind park	Sandbanks		1.692
Coates et al. 2016, Short-term effects of fishery exclusion in offshore wind farms, Fisheries Research, 179, 131-138	Wind park	Sandbanks		0.767
Coates et al. 2016, Short-term effects of fishery exclusion in offshore wind farms, Fisheries Research, 179, 131-138	Wind park	Sandbanks		0.945
Coates et al. 2016, Short-term effects of fishery exclusion in offshore wind farms, Fisheries Research, 179, 131-138	Wind park	Sandbanks		0.499
Coates et al. 2016, Short-term effects of fishery exclusion in offshore wind farms, Fisheries Research, 179, 131-138	Wind park	Sandbanks		0.469
Coates et al. 2016, Short-term effects of fishery exclusion in offshore wind farms, Fisheries Research, 179, 131-138	Wind park	Sandbanks		0.456
Coates et al. 2016, Short-term effects of fishery exclusion in offshore wind farms, Fisheries Research, 179, 131-138	Wind park	Sandbanks		0.647
Coates et al. 2016, Short-term effects of fishery exclusion in offshore wind farms, Fisheries Research, 179, 131-138	Wind park	Sandbanks		0.537
Coates et al. 2016, Short-term effects of fishery exclusion in offshore wind farms, Fisheries Research, 179, 131-138	Wind park	Sandbanks		0.535
Coates et al. 2016, Short-term effects of fishery exclusion in offshore wind farms, Fisheries Research, 179, 131-138	Wind park	Sandbanks		0.797
Coates et al. 2016, Short-term effects of fishery exclusion in offshore wind farms, Fisheries Research, 179, 131-138	Wind park	Sandbanks		2.458
Coates et al. 2016, Short-term effects of fishery exclusion in offshore wind farms, Fisheries Research, 179, 131-138	Wind park	Sandbanks		2.093
Coates et al. 2016, Short-term effects of fishery exclusion in offshore wind farms, Fisheries Research, 179, 131-138	Wind park	Sandbanks		2.966
Coates et al. 2016, Short-term effects of fishery exclusion in offshore wind farms, Fisheries Research, 179, 131-138	Wind park	Sandbanks		0.901
Coates et al. 2016, Short-term effects of fishery exclusion in offshore wind farms, Fisheries Research, 179, 131-138	Wind park	Sandbanks		0.249
Coates et al. 2016, Short-term effects of fishery exclusion in offshore wind farms, Fisheries Research, 179, 131-138	Wind park	Sandbanks		0.863
Coates et al. 2016, Short-term effects of fishery exclusion in offshore wind farms, Fisheries Research, 179, 131-138	Wind park	Sandbanks		0.115
Coates et al. 2016, Short-term effects of fishery exclusion in offshore wind farms, Fisheries Research, 179, 131-138	Wind park	Sandbanks		0.620
Coates et al. 2016, Short-term effects of fishery exclusion in offshore wind farms, Fisheries Research, 179, 131-138	Wind park	Sandbanks		5.000
Coates et al. 2016, Short-term effects of fishery exclusion in offshore wind farms, Fisheries Research, 179, 131-138	Wind park	Sandbanks		0.058
Coates et al. 2016, Short-term effects of fishery exclusion in offshore wind farms, Fisheries Research, 179, 131-138	Wind park	Sandbanks		5.000
Coates et al. 2016, Short-term effects of fishery exclusion in offshore wind farms, Fisheries Research, 179, 131-138	Wind park	Sandbanks		1.526
Coates et al. 2016, Short-term effects of fishery exclusion in offshore wind farms, Fisheries Research, 179, 131-138	Wind park	Sandbanks		1.604
Coates et al. 2016, Short-term effects of fishery exclusion in offshore wind farms, Fisheries Research, 179, 131-138	Wind park	Sandbanks		1.469
Coates et al. 2016, Short-term effects of fishery exclusion in offshore wind farms, Fisheries Research, 179, 131-138	Wind park	Sandbanks		3.104
