# Supplementary material for: Assessing cumulative impacts of human-induced pressures on reef and sandbank habitats and associated biotopes in the northeastern Baltic Sea
Source: Mar Pollut Bull. 2022 Oct;183:114042. doi: 10.1016/j.marpolbul.2022.114042 (PMC9551195; doi:10.1016/j.marpolbul.2022.114042)
Supplement: Supplementary file 2 — Supplementary material 2: Prediction maps [file mmc2.pdf]

Supplementary material 2

1. Current nutrient load

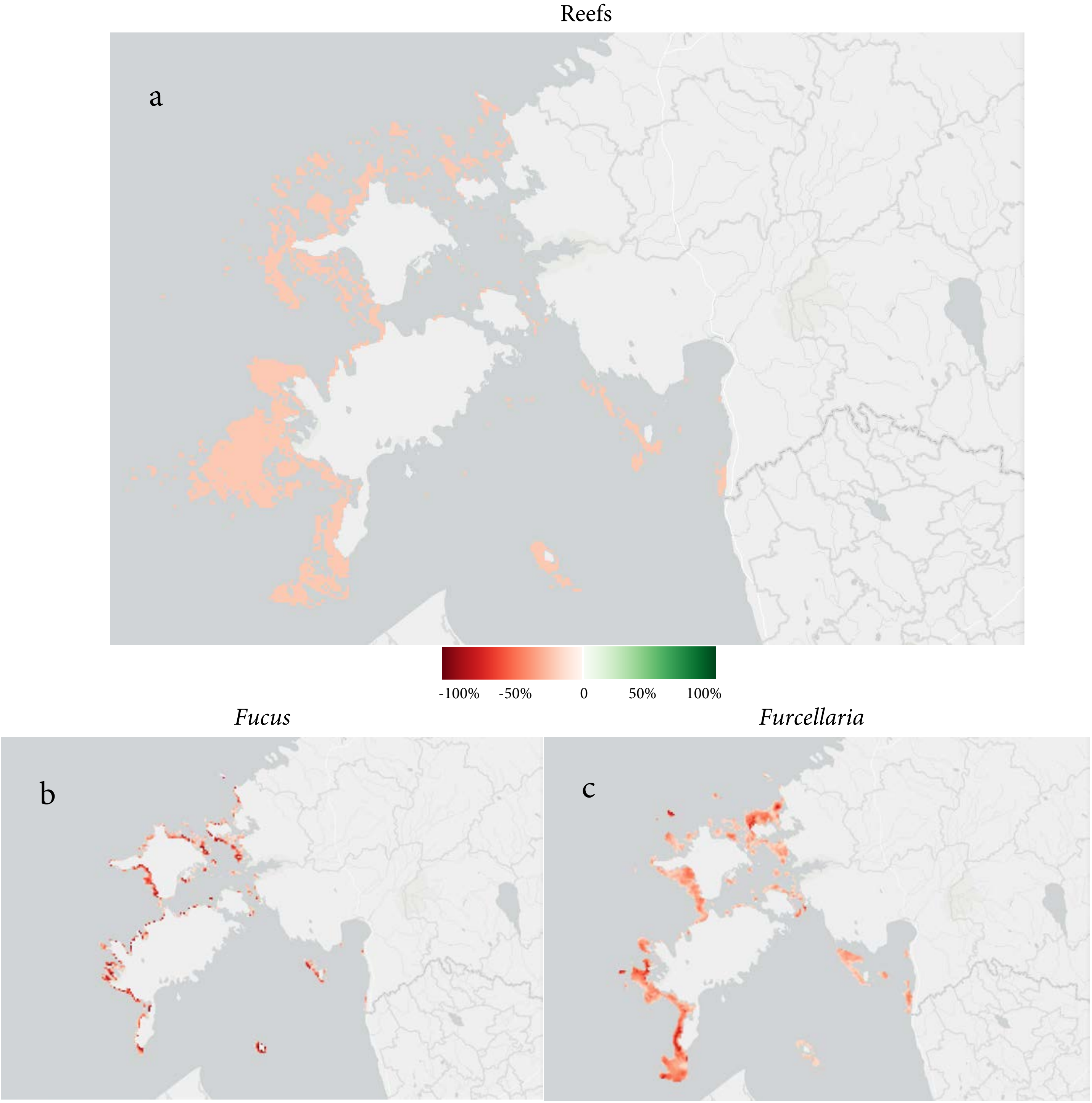

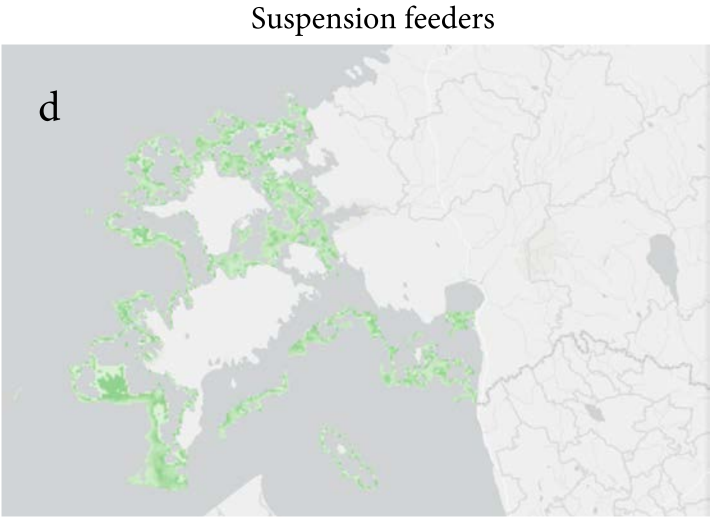

Figure 1: Predicted areal change in the current nutrient load scenario in (a) reefs, (b) *Fucus*, (c) *Furcellaria*, and (d) suspension feeders habitats (change in km^2 in a 1 km^2 cell).

2. Future nutrient reduction (HELCOM MAI target of -25% reduction)

Fucus

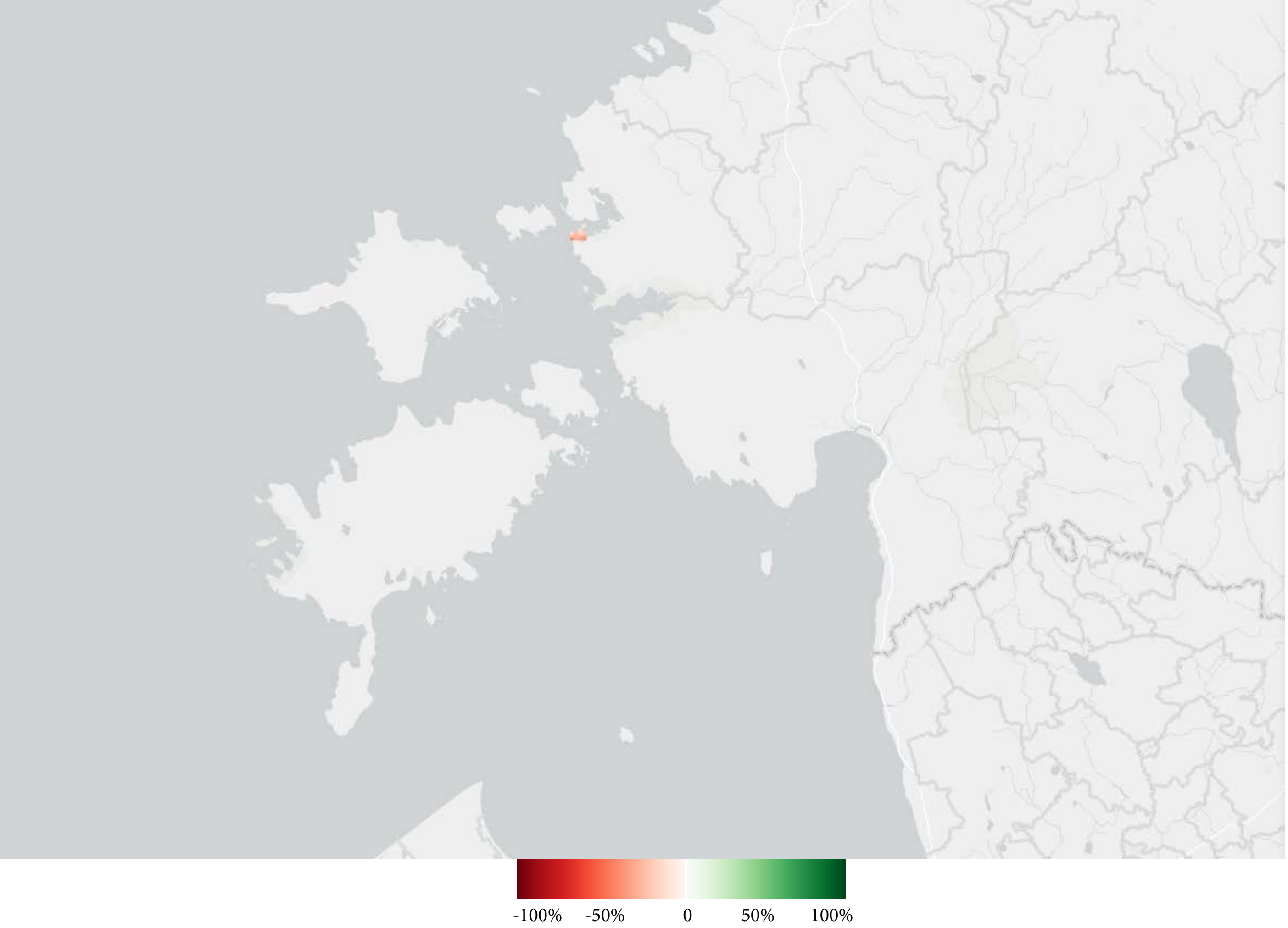

Figure 2: Predicted areal change in the future nutrient reduction scenario according to HELCOM MAI target in *Fucus* habitat (change in km^2 in a 1 km^2 cell).

3. Presence of nonnative species (round goby and mud crab)

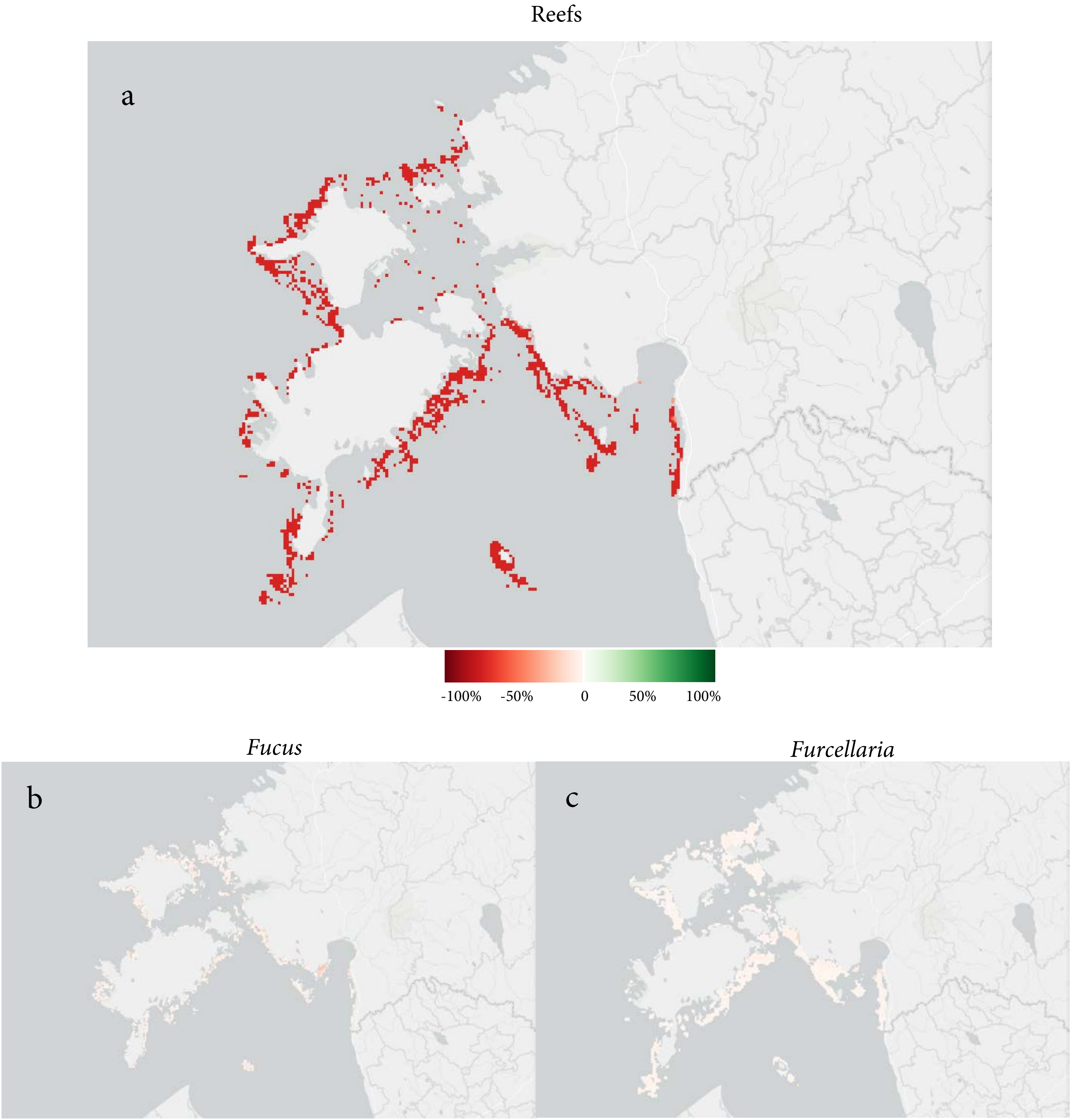

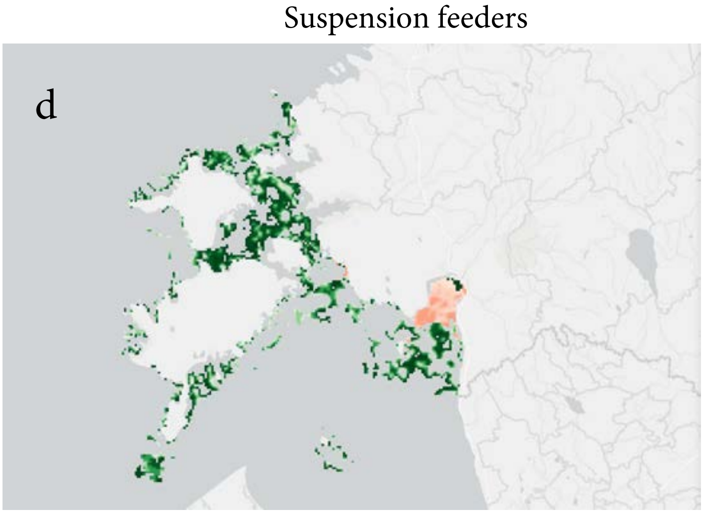

Figure 3: Predicted areal change in the presence of nonnative round goby and mud crab scenario in (a) reefs, (b) Fucus, (c) Furcellaria, and (d) suspension feeders habitats (change in km^2 in a 1 km^2 cell).

4. Projected wind parks (according to the Estonian maritime spatial plan)

Suspension feeders

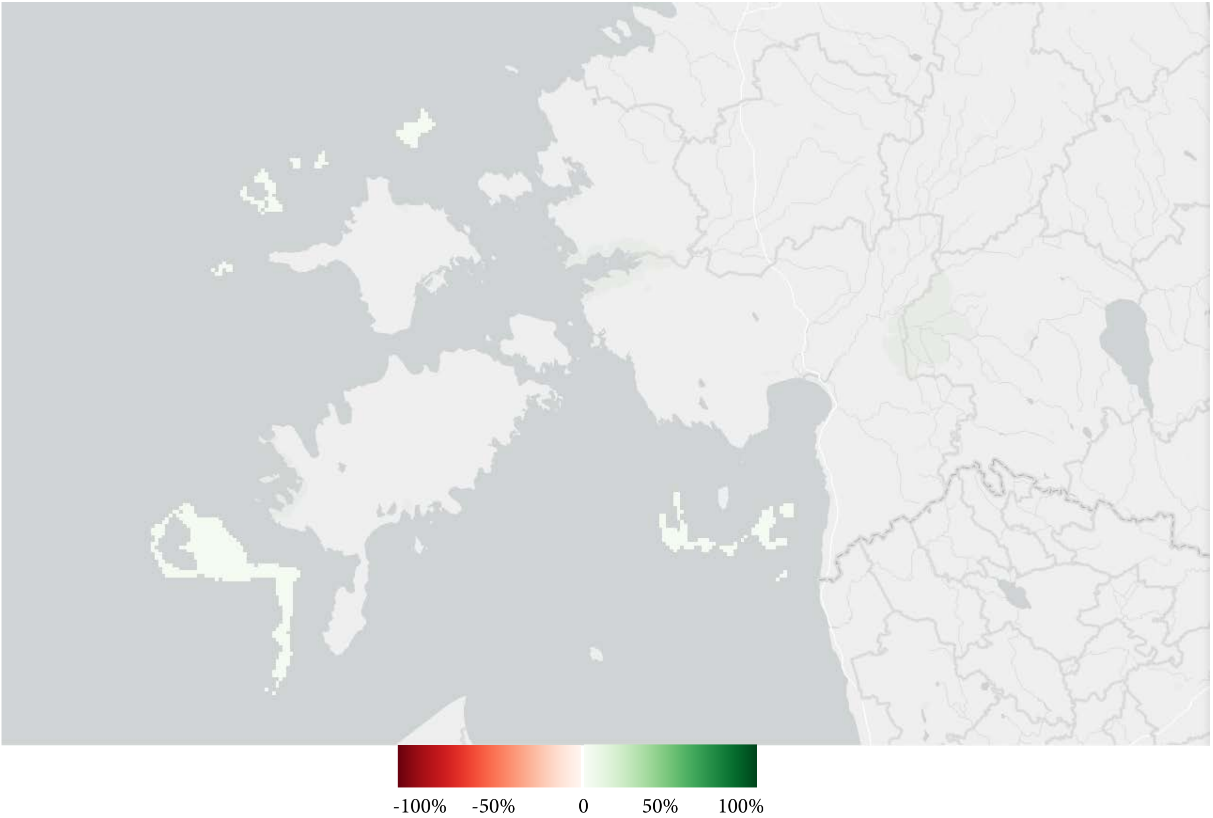

Figure 4: Predicted areal change in projected wind park sites, according to Estonian maritime spatial plan, in suspension feeders habitat (change in km^2 in a 1 km^2 cell).

5. Current nutrient load + nonnative species

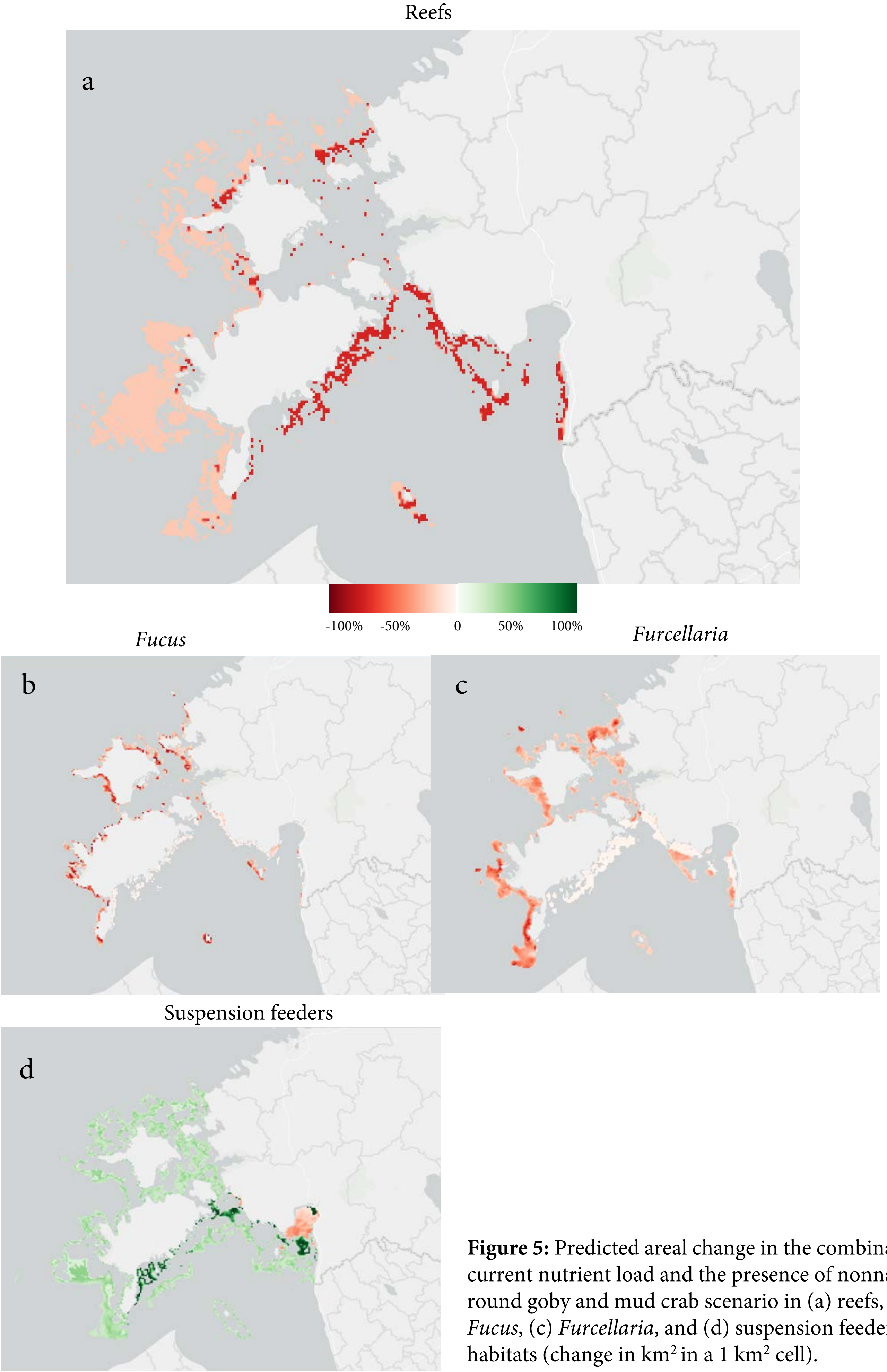

Figure 5: Predicted areal change in the combination of current nutrient load and the presence of nonnative round goby and mud crab scenario in (a) reefs, (b) *Fucus*, (c) *Furcellaria*, and (d) suspension feeders habitats (change in km^2 in a 1 km^2 cell).

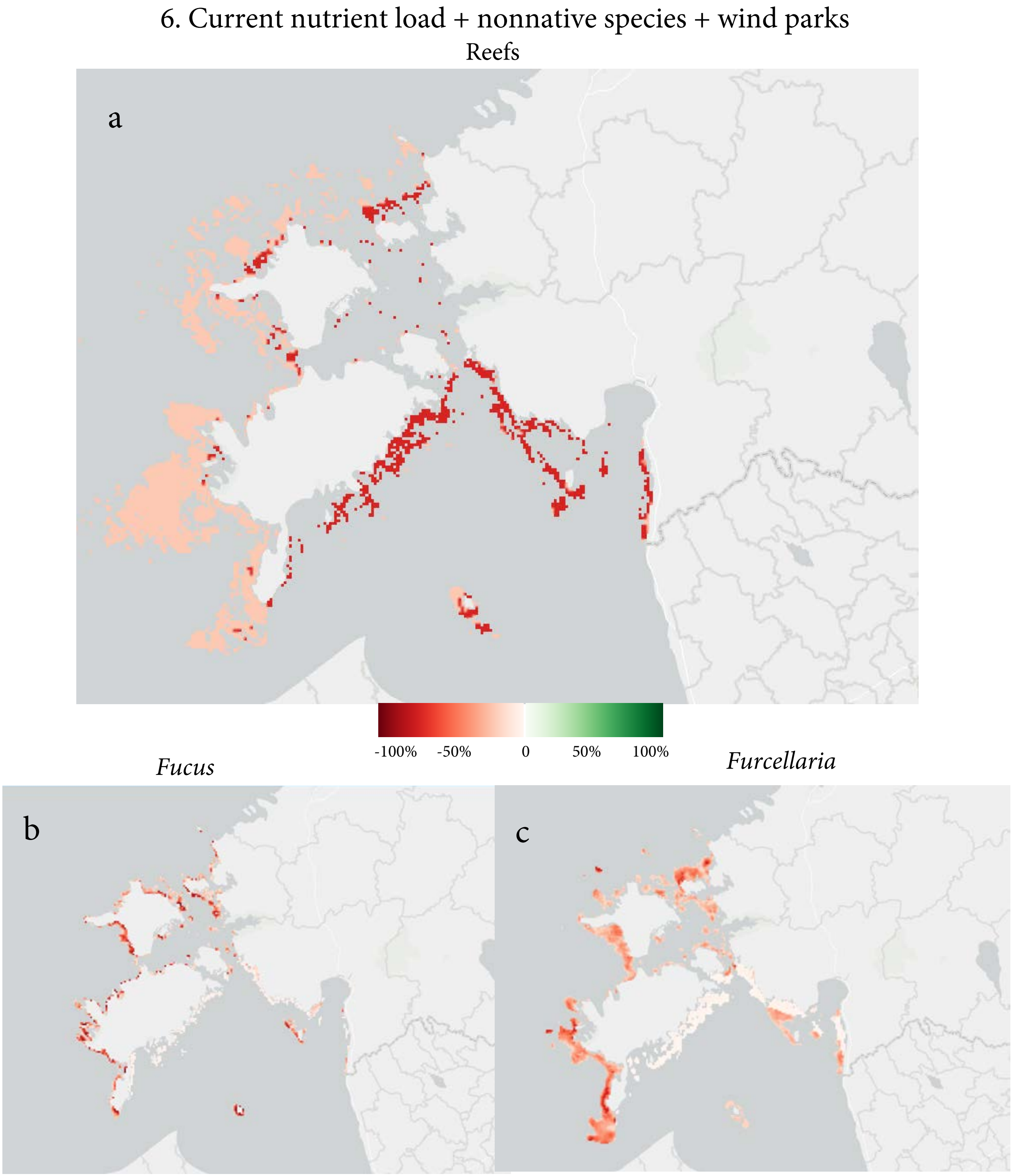

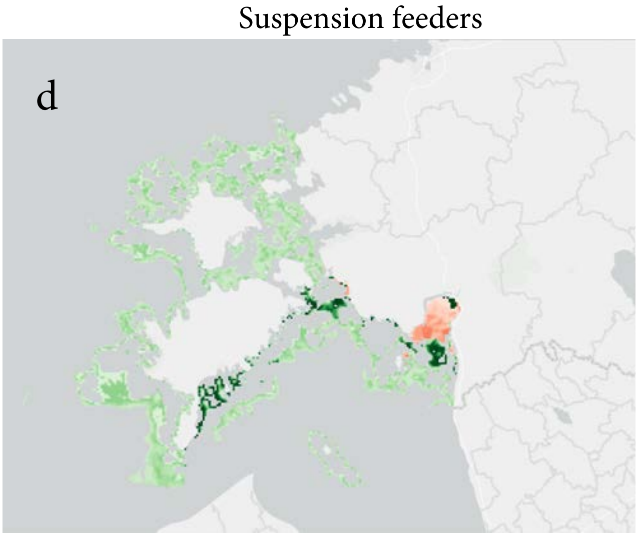

Figure 6: Predicted areal change in the combination of current nutrient load, the presence of nonnative round goby and mud crab, and projected wind park sites scenario in (a) reefs, (b) *Fucus*, (c) *Furcellaria*, and (d) suspension feeders habitats (change in km² in a 1 km² cell).

7. Future nutrient reduction + nonnative species + wind parks

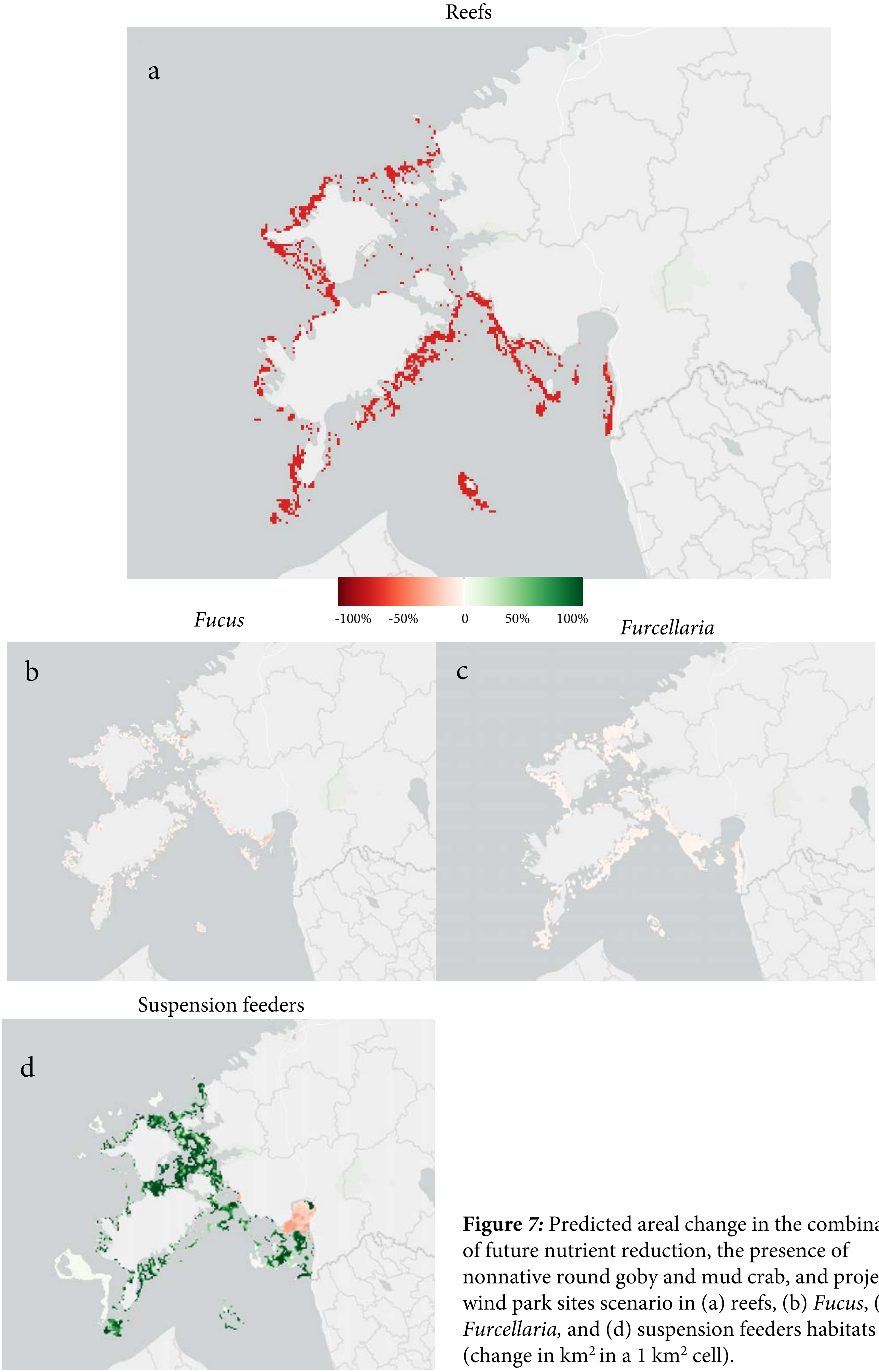

Figure 7: Predicted areal change in the combination of future nutrient reduction, the presence of nonnative round goby and mud crab, and projected wind park sites scenario in (a) reefs, (b) *Fucus*, (c) *Furcellaria*, and (d) suspension feeders habitats (change in km^2 in a 1 km^2 cell).

1. Current nutrient load

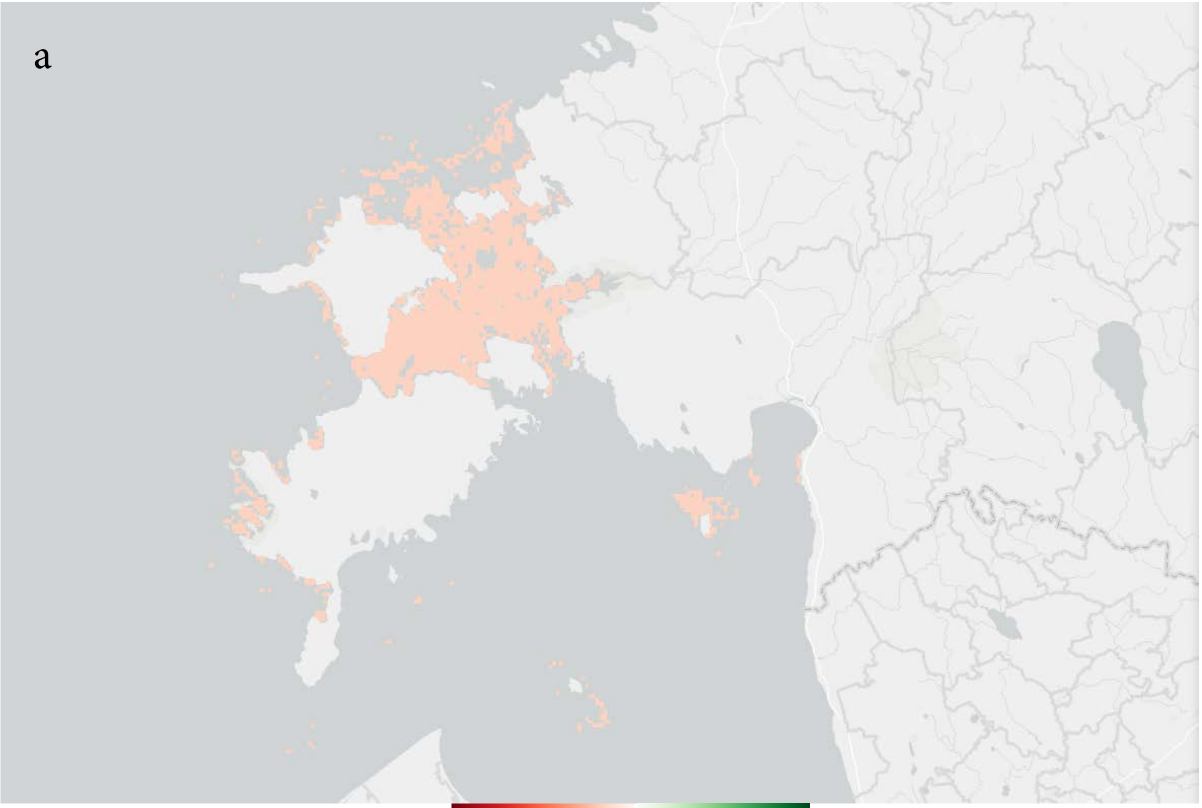

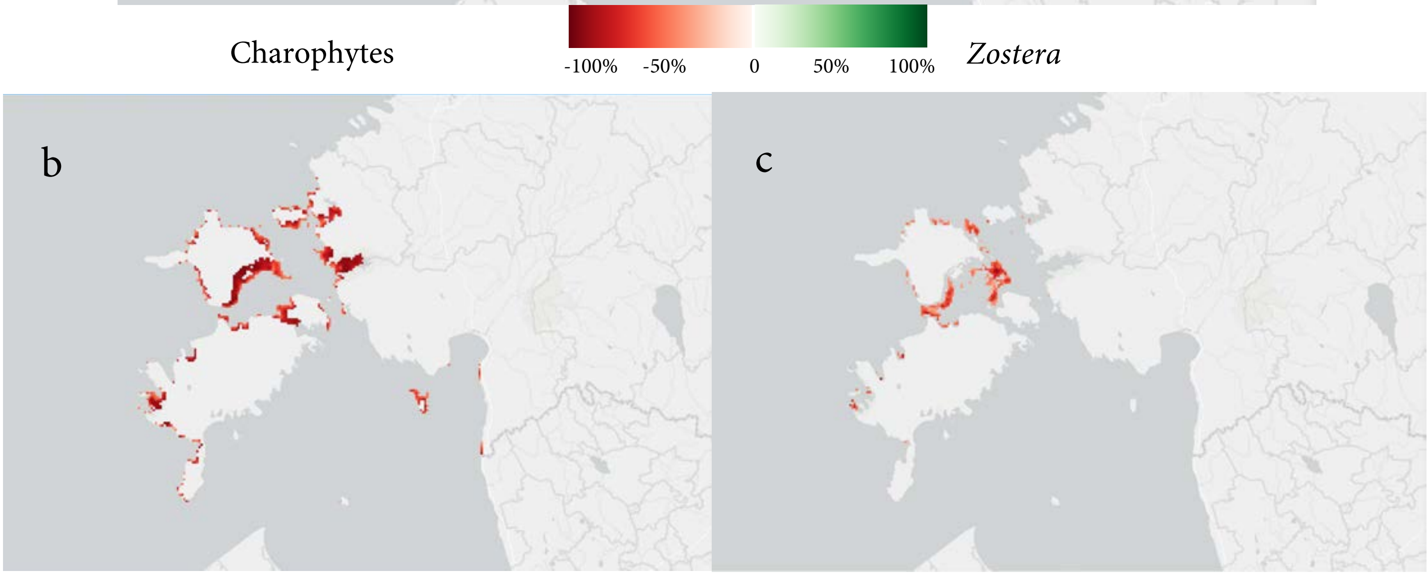

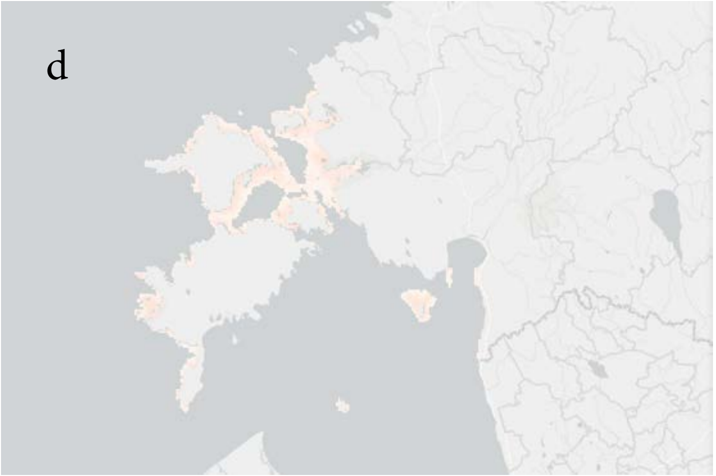

Figure 8: Predicted areal change in the current nutrient load scenario in (a) sandbanks, (b) Charophyes (c) *Zostera*, and (d) higher plants habitats (change in km^2 in a 1 km^2 cell).

2. Future nutrient load (HELCOM MAI target of -25% reduction)

Sandbanks

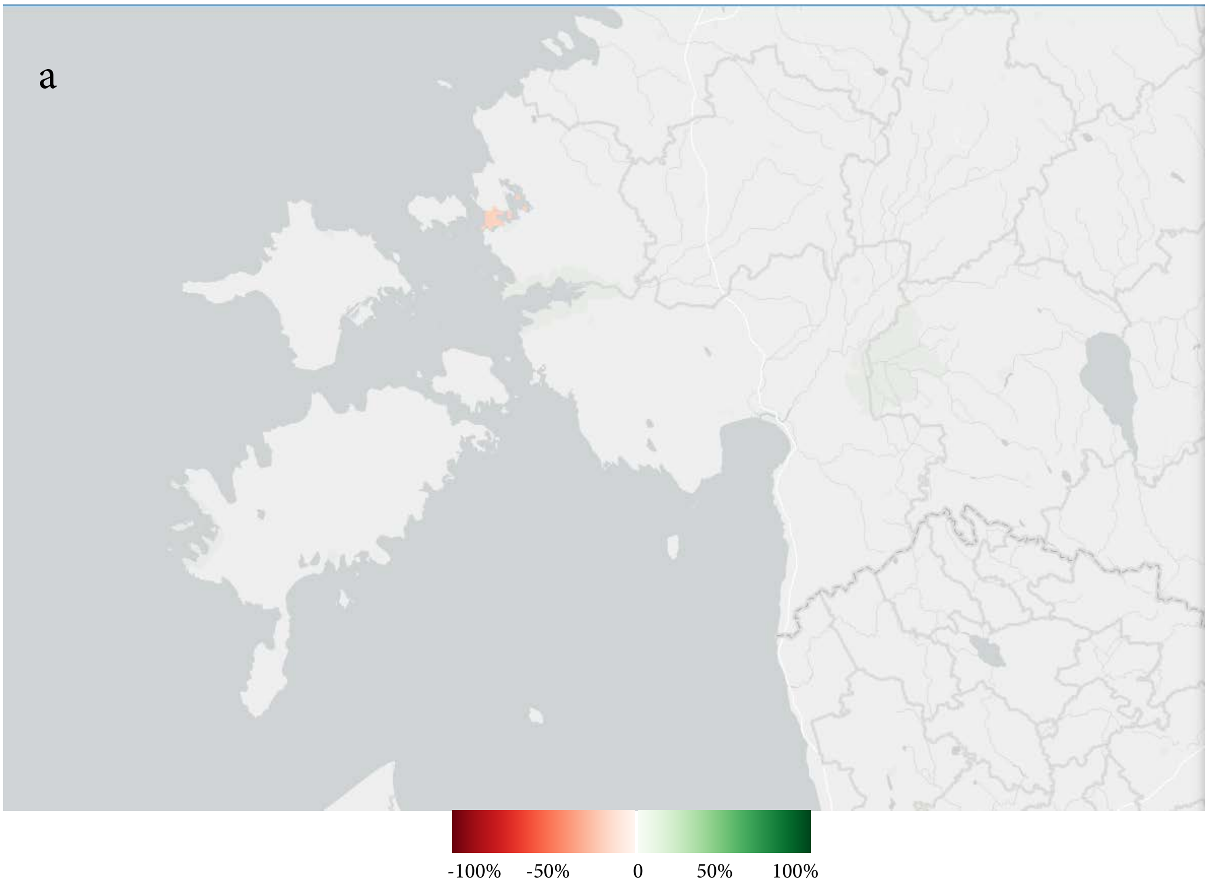

Charophytes | *Zostera*

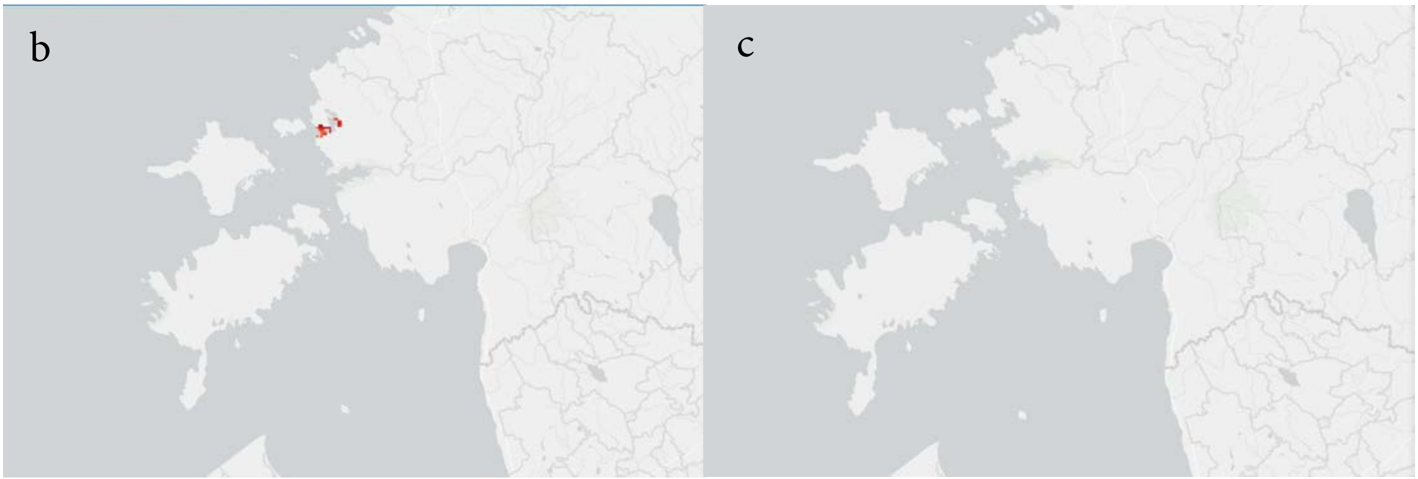

Higher plants

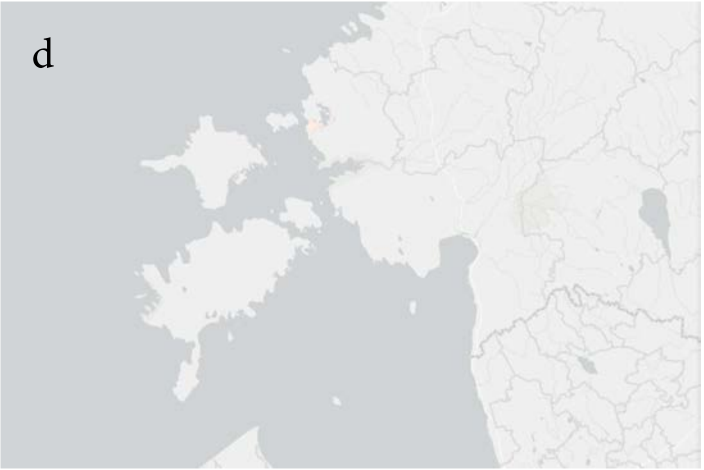

Figure 9: Predicted areal change in the future nutrient reduction scenario in (a) sandbanks, (b) Charophyes (c) *Zostera*, and (d) higher plants habitats (change in km^2 in a 1 km^2 cell).

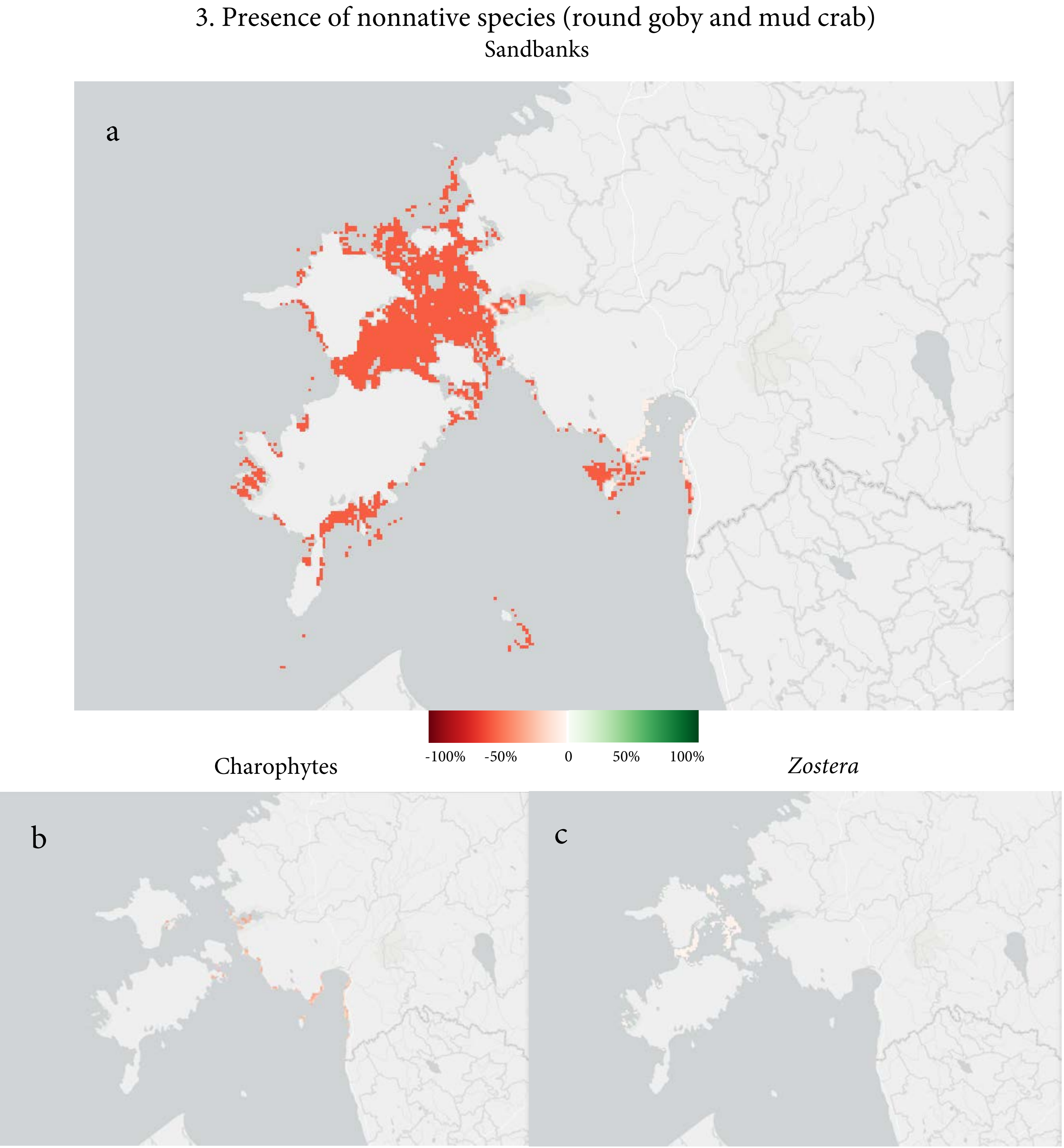

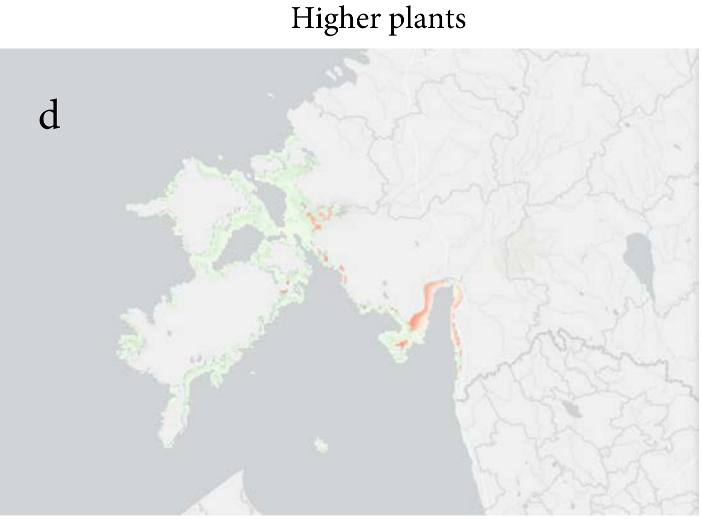

Figure 10: Predicted areal change in the presence of nonnative round goby and mud crab scenario in (a) sandbanks, (b) Charophyes (c) *Zostera*, and (d) higher plants habitats (change in km^2 in a 1 km^2 cell).

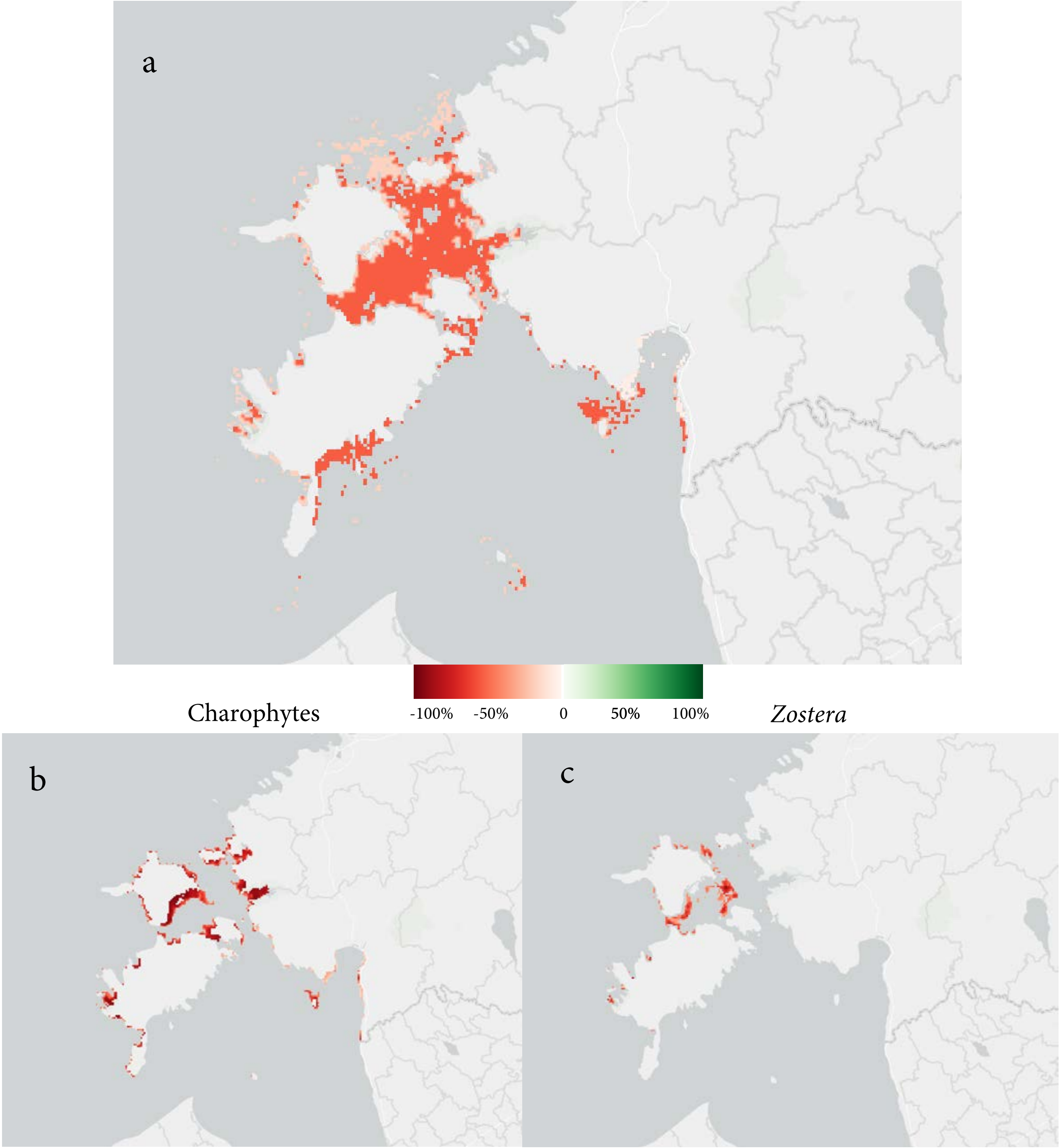

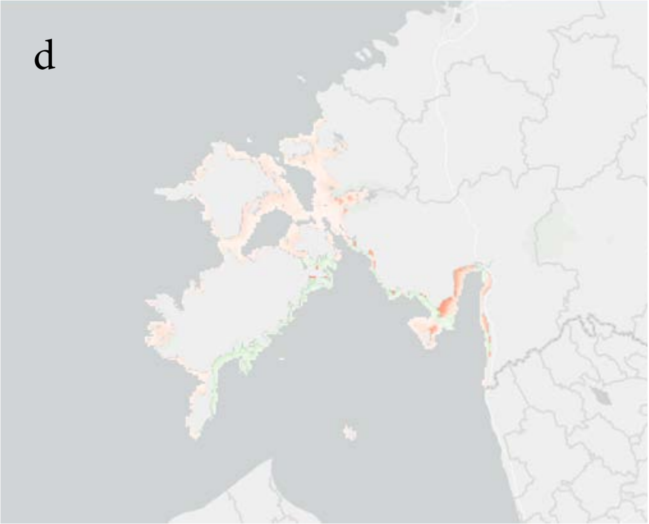

Figure 11: Predicted areal change in the combination of current nutrient load, the presence of nonnative round goby and mud crab scenario in (a) sandbanks, (b) Charophyes (c) *Zostera*, and (d) higher plants habitats (change in km^2 in a 1 km^2 cell).

6. Current nutrient load + nonnative species + wind parks

Sandbanks

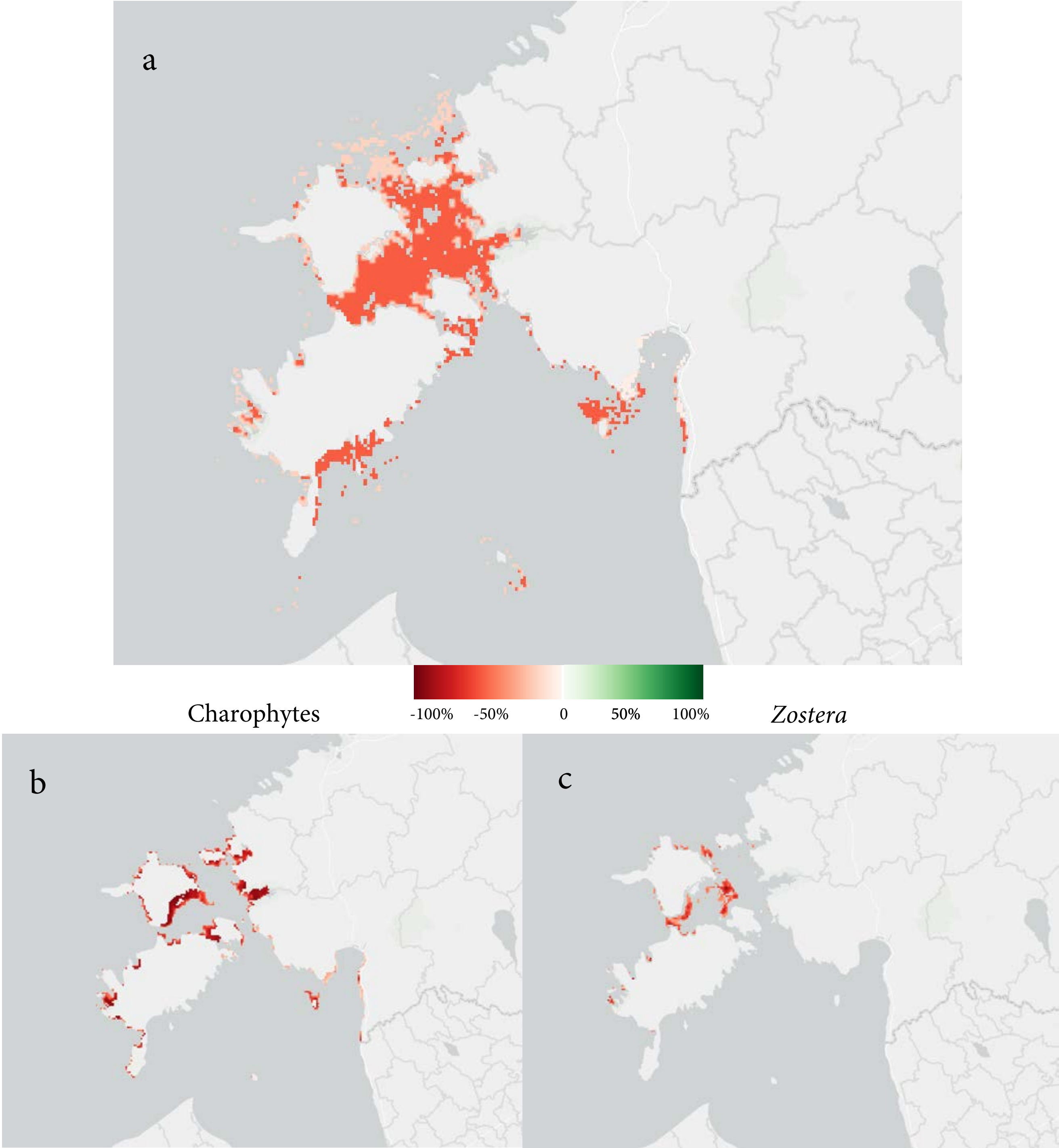

Higher plants

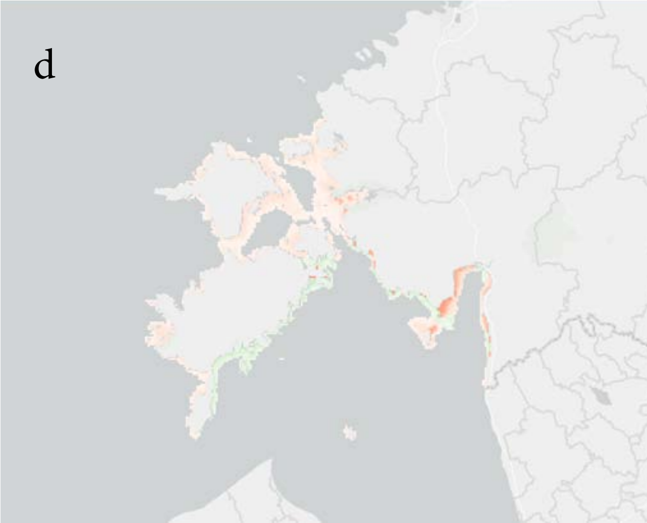

Figure 12: Predicted areal change in the combination of current nutrient load, the presence of nonnative round goby and mud crab, and projected wind park sites scenario in (a) sandbanks, (b) Charophyes (c) *Zostera*, and (d) higher plants habitats (change in km^2 in a 1 km^2 cell).

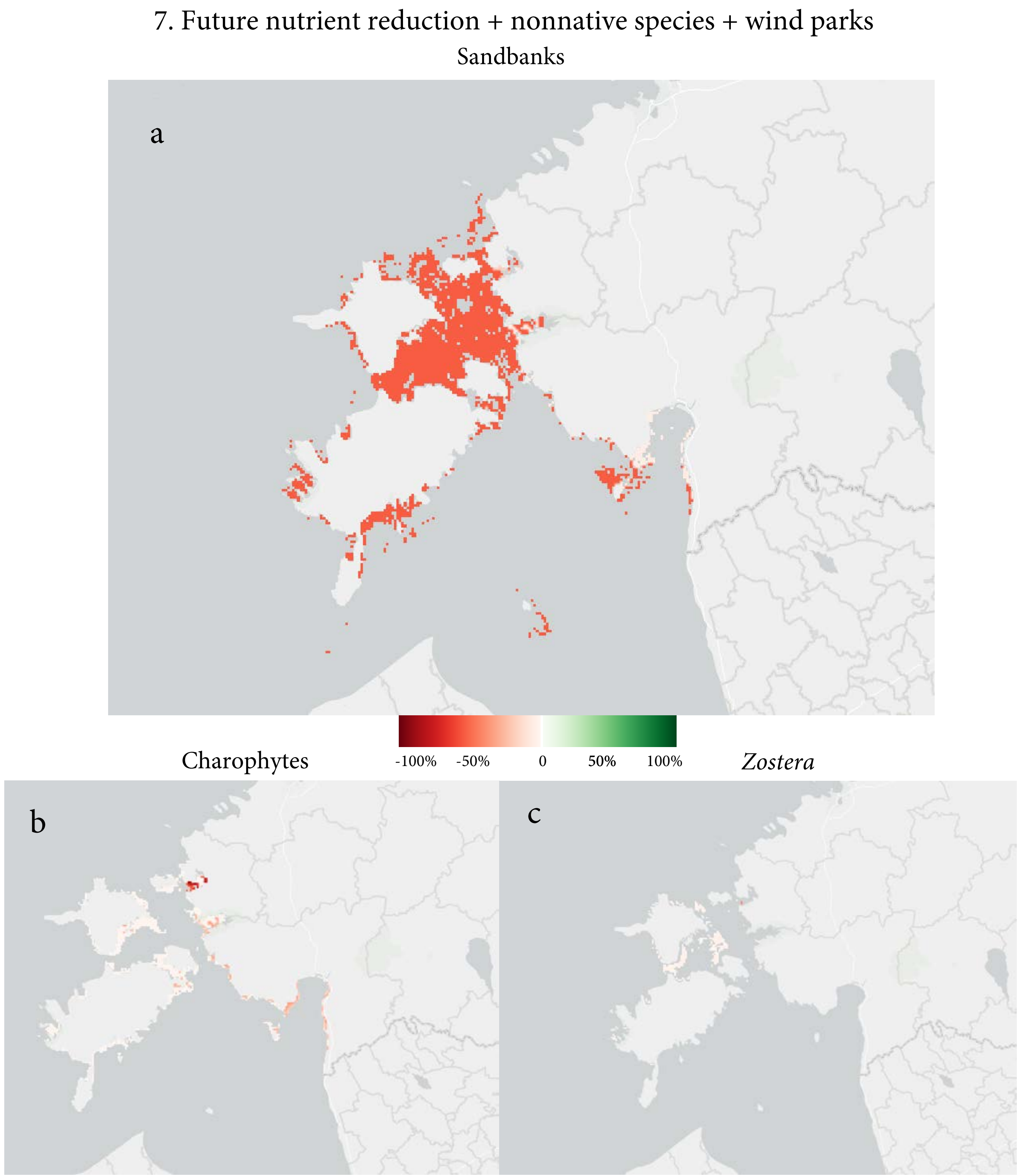

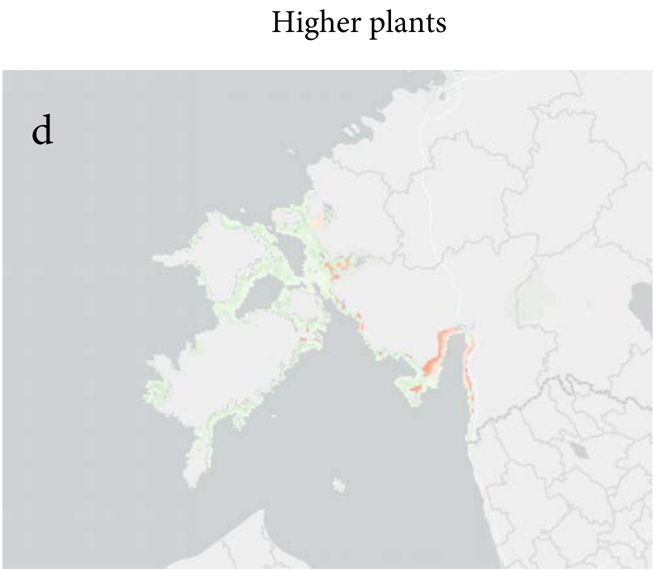

Figure 13: Predicted areal change in the combination of future nutrient reduction, the presence of nonnative round goby and mud crab, and projected wind park sites scenario in (a) sandbanks, (b) Charophyes (c) *Zostera*, and (d) higher plants habitats (change in km^2 in a 1 km^2 cell).

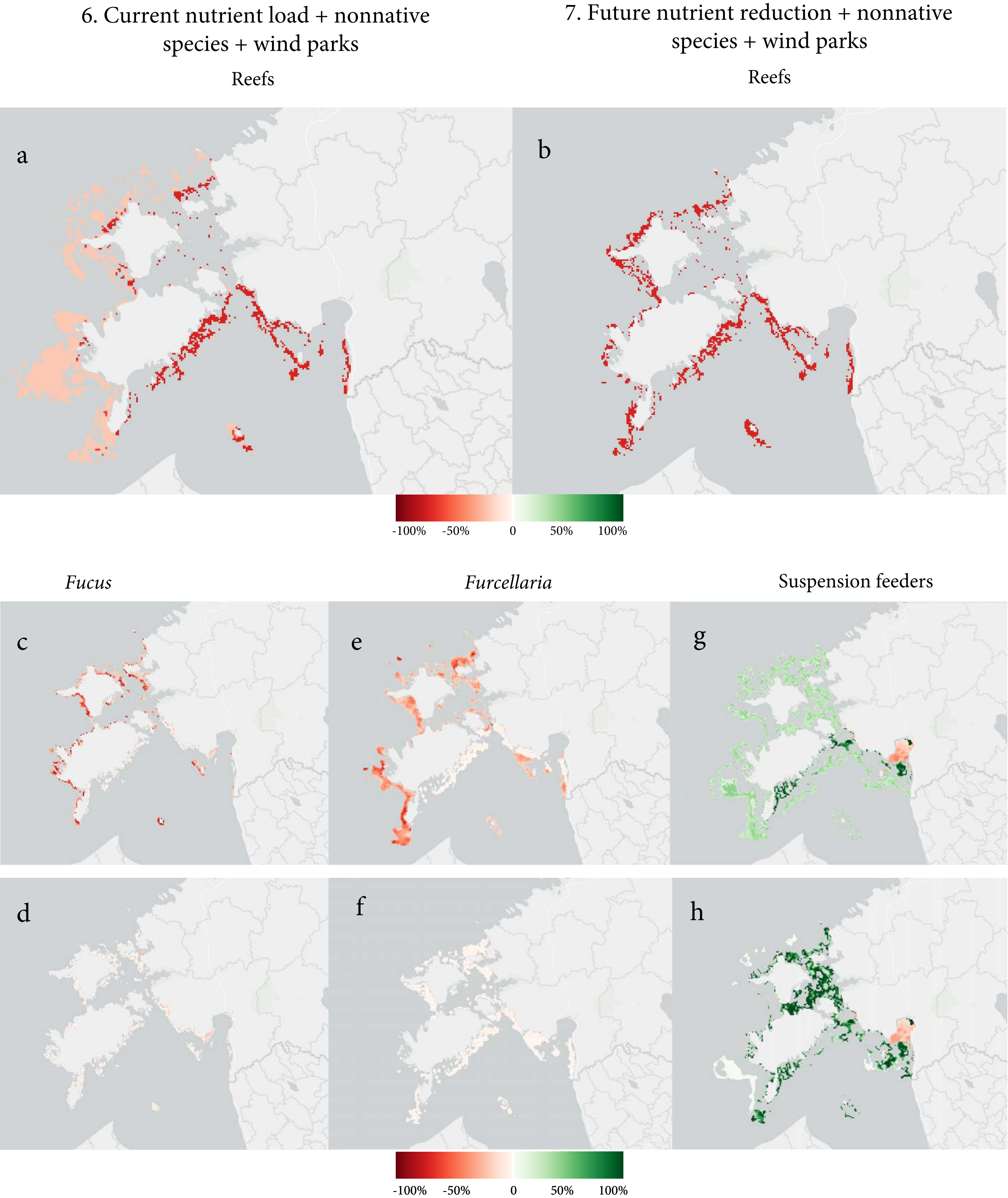

Figure 14: Habitat change comparing the combinations of current nutrient load + nonnative species + wind park development *versus* 25% nutrient reduction + nonnative species + wind park development scenarios. (a, b) show the difference in larger-scale reef environment. Differences in habitat change in associated habitat types are (c, d) in *Fucus* habitat, (e, f) in *Furcellaria* habitat, and (g, h) in suspension feeders habitat (change in km^2 in a 1 km^2 cell).

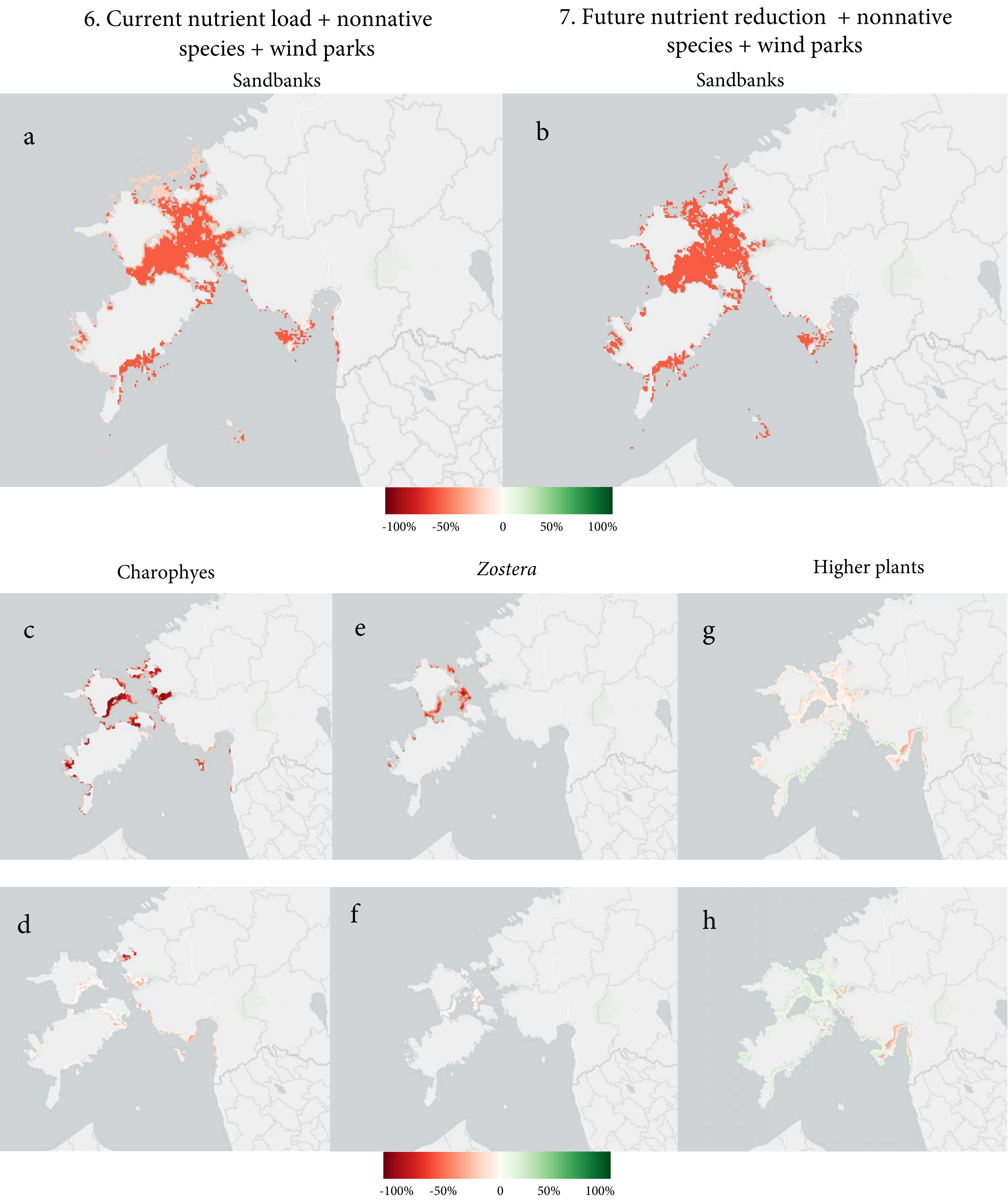

Figure 15: Habitat change comparing the combinations of current nutrient load + nonnative species + wind park development *versus* 25% nutrient reduction + nonnative species + wind park development scenarios. (a, b) show the difference in a larger-scale sandbank environment. Differences in habitat change in associated habitat types are (c, d) in Charophytes habitat, (e, f) in *Zostera* habitat, and (g, h) in higher plants habitat (change in km^2 in a 1 km^2 cell).
